# Methylprednisolone Pulses Plus Tacrolimus in Addition to Standard of Care vs. Standard of Care Alone in Patients With Severe COVID-19. A Randomized Controlled Trial

**DOI:** 10.3389/fmed.2021.691712

**Published:** 2021-06-14

**Authors:** Xavier Solanich, Arnau Antolí, Gemma Rocamora-Blanch, Núria Padullés, Marta Fanlo-Maresma, Adriana Iriarte, Francesca Mitjavila, Olga Capdevila, Antoni Riera-Mestre, Jordi Bas, Vanesa Vicens-Zygmunt, Jordi Niubó, Nahum Calvo, Santiago Bolivar, Raúl Rigo-Bonnin, Anna Mensa-Vilaró, Laura Arregui, Cristian Tebe, Sebastià Videla, Pilar Hereu, Xavier Corbella

**Affiliations:** ^1^Department of Internal Medicine, Hospital Universitari de Bellvitge, Bellvitge Biomedical Research Institute (IDIBELL), Barcelona, Spain; ^2^Department of Pharmacy, Hospital Universitari de Bellvitge, Bellvitge Biomedical Research Institute (IDIBELL), Barcelona, Spain; ^3^Faculty of Medicine and Health Sciences, Universitat de Barcelona, Barcelona, Spain; ^4^Department of Immunology, Hospital Universitari de Bellvitge, Bellvitge Biomedical Research Institute (IDIBELL), Barcelona, Spain; ^5^Department of Pneumology, Hospital Universitari de Bellvitge, Bellvitge Biomedical Research Institute (IDIBELL), Barcelona, Spain; ^6^Department of Microbiology, Hospital Universitari de Bellvitge, Bellvitge Biomedical Research Institute (IDIBELL), Barcelona, Spain; ^7^Department of Diagnostic Imaging, Hospital Universitari de Bellvitge, Bellvitge Biomedical Research Institute (IDIBELL), Barcelona, Spain; ^8^Department of Clinical Laboratory, Hospital Universitari de Bellvitge, Bellvitge Biomedical Research Institute (IDIBELL), Barcelona, Spain; ^9^Department of Immunology, Hospital Clínic de Barcelona, Institut d'Investigacions Biomèdiques August Pi i Sunyer, Barcelona, Spain; ^10^HUB-ICO-IDIBELL Biobank, Spanish Clinical Research Network, Bellvitge Biomedical Research Institute (IDIBELL), Barcelona, Spain; ^11^Department of Biostatistics, Bellvitge Biomedical Research Institute (IDIBELL), Barcelona, Spain; ^12^Department of Clinical Pharmacology, Hospital Universitari de Bellvitge, Bellvitge Biomedical Research Institute (IDIBELL), Barcelona, Spain; ^13^Clinical Research and Clinical Trial Unit (UICEC-IDIBELL), Spanish Clinical Research Network, Bellvitge Biomedical Research Institute (IDIBELL), Barcelona, Spain; ^14^School of Medicine, Universitat Internacional de Catalunya, Barcelona, Spain

**Keywords:** COVID-19, SARS-CoV-2, methylprednisolone, tacrolimus, inflammation, lung injury

## Abstract

**Introduction:** Severe lung injury is triggered by both the SARS-CoV-2 infection and the subsequent host-immune response in some COVID-19 patients.

**Methods:** We conducted a randomized, single-center, open-label, phase II trial with the aim to evaluate the efficacy and safety of methylprednisolone pulses and tacrolimus plus standard of care (SoC) vs. SoC alone, in hospitalized patients with severe COVID-19. The primary outcome was time to clinical stability within 56 days after randomization.

**Results:** From April 1 to May 2, 2020, 55 patients were prospectively included for subsequent randomization; 27 were assigned to the experimental group and 28 to the control group. The experimental treatment was not associated with a difference in time to clinical stability (hazard ratio 0.73 [95% CI 0.39–1.37]) nor most secondary outcomes. Median methylprednisolone cumulative doses were significantly lower (360 mg [IQR 360–842] vs. 870 mg [IQR 364–1451]; p = 0.007), and administered for a shorter time (median of 4.00 days [3.00–17.5] vs. 18.5 days [3.00–53.2]; p = 0.011) in the experimental group than in the control group. Although not statistically significant, those receiving the experimental therapy showed a numerically lower all-cause mortality than those receiving SoC, especially at day 10 [2 (7.41%) vs. 5 (17.9%); OR 0.39 (95% CI 0.05–2.1); *p* = 0.282]. The total number of non-serious adverse events was 42 in each the two groups. Those receiving experimental treatment had a numerically higher rate of non-serious infectious adverse events [16 (38%) vs. 10 (24%)] and serious infectious adverse events [7 (35%) vs. 3 (23%)] than those receiving SoC.

**Conclusions:** The combined use of methylprednisolone pulses plus tacrolimus, in addition to the SoC, did not significantly improve the time to clinical stability or other secondary outcomes compared with the SoC alone in severe COVID-19. Although not statistically significant, patients receiving the experimental therapy had numerically lower all-cause mortality than those receiving SoC, supporting recent non-randomized studies with calcineurin inhibitors. It is noteworthy that the present trial had a limited sample size and several other limitations. Therefore, further RCTs should be done to assess the efficacy and safety of tacrolimus to tackle the inflammatory stages of COVID-19.

**Clinical Trial Registration:** Identifier [NCT04341038/EudraCT: 2020-001445-39].

## Introduction

In December 2019, a new type of human coronavirus (SARS-CoV-2), causing an emerging diseases (COVID-19), was first recognized in China and spread globally (1, 2). The COVID-19 was declared a pandemic by the WHO on March 12, 2020 (3), and it continues to spread worldwide, causing considerable morbimortality and economic damage.

SARS-CoV-2 has evolved some mechanisms to disturb host-immune response. In fact, impaired interferon (IFN) signature in early stages leads to a persistent blood viral load and a later hyper-inflammatory response that has been related with a worse COVID-19 outcome (4, 5). Accordingly, antiviral followed by anti-inflammatory drugs have been recommended (6). While some immunosuppressive treatments could be potentially harmful, others have been suggested for treating the disproportionate inflammation triggered by the SARS-CoV-2 infection (7).

When the TACROVID trial was designed in March 2020, data from the main COVID-19 randomized controlled trials (RCT) (8–10) were still not available and there were no therapies for treating the COVID-19 illness other than supportive care. Due to the lack of evidence-based treatments, a large number of patients received off-label and compassionate therapies, based on their *in vitro* antiviral or immunomodulatory properties. The repurposing of older drugs was the initial main strategy given their proven safety profile (11). Today, RCTs are still needed in order to provide evidence-based effective and safe therapies for COVID-19 management (12).

Our hypothesis was that methylprednisolone pulses plus tacrolimus could be an effective and safe drug combination for severe COVID-19 patients. Accordingly, given the health emergency due to the rapid spread of SARS-CoV-2 worldwide, we conducted a proof-of-concept study in a randomized, single-center, open-label clinical trial with the aim to evaluate the efficacy and safety of methylprednisolone pulses and tacrolimus plus standard of care (SoC), vs. SoC alone, in severe COVID-19 patients with lung injury and systemic hyperinflammatory syndrome.

The rationale for the current RCT was based on the fact that corticosteroids, such as methylprednisolone, are a pillar in the treatment of multiple inflammatory diseases, with several mechanisms of action impacting both the innate and adaptive arms of immunity. Regarding tacrolimus, the reason for its use was based on both the anti-inflammatory and anti-viral actions of calcineurin inhibitors (CNIs). As an immunomodulatory agent, tacrolimus impairs lymphocyte function and consequently decreases in pro-inflammatory cytokines (7, 13). In this respect, severe COVID-19 disease presents a similar clinical and cytokine profile to other disorders like secondary hemophagocytic lymphohistiocytosis (14), where CNIs play a central role in its treatment (15). Additionally, several human coronavirus replication depends on immunophilin pathways, which can be inhibited by CNIs in cell culture (16, 17). Based on these two mechanisms, it has been suggested that CNIs could be used to treat COVID-19 (18). In fact, recent non-randomized studies suggest that cyclosporine could reduce mortality, mainly in patients with moderate to severe COVID-19 (19, 20). Our study is the first RCT assessing the effect of corticosteroids plus a CNI (tacrolimus) in hospitalized patients with severe COVID-19. They are low-cost drugs with a well-known safety profile that could be produced on a large scale if they were effective at treating COVID-19.

## Methods

### Study Design

TACROVID was a pragmatic, randomized (1:1) with parallel-groups, open-label, single-center, phase II clinical trial to evaluate the efficacy and safety of methylprednisolone pulses and tacrolimus plus SoC, vs. SoC alone, in severe COVID-19 patients with lung injury and systemic hyperinflammatory syndrome.

The TACROVID trial was conducted at Hospital Universitari de Bellvitge (HUB), a 750-bed tertiary care public hospital for adults in Barcelona (Catalonia, Spain). HUB is the reference hospital for high complexity patients from the southern area of Catalonia, a region of ~2 million inhabitants.

In March 2020, the HUB's Ethical Committee for Drug Research and the Spanish Agency for Drugs and Health Products approved the protocol and informed consent form (ICF). This trial complies with the Declaration of Helsinki and Good Clinical Practice guidelines, and personal data protection as required by Spanish law (LOPD 3/2018). The trial registration numbers are NCT04341038 and EudraCT 2020-001445-39. All patients (or a legal representative if patients were unable) had to provide ICF prior to initiation of the trial procedures. The protocol is available online (21).

### Population

Patients were included in the trial if they met all the inclusion criteria and none of the exclusion criteria. *Inclusion criteria:* (1) COVID-19 infection confirmed by nasopharyngeal RT-PCR; (2) New pulmonary infiltrates (either by chest X-ray or computerized axial tomography); (3) Respiratory failure defined by PaO_2_/FiO_2_ < 300 or SpO_2_/FiO_2_ < 220; (4) High analytical inflammatory parameters: CRP > 100 mg/L, and/or D-Dimer > 1,000 μg/L, and/or Ferritin > 1,000 μg/L. *Exclusion criteria:* (1) Critically ill patients with life expectancy ≤ 24 h; (2) Glomerular filtration ≤ 30 ml/min/1.73m^2^; (3) Leukopenia ≤ 4,000 cells/μl or other conditions that cause immunosuppression; (4) Concomitant potentially serious infections; (5) Contraindication for the use of corticosteroids or tacrolimus according to the Summary of Product Characteristics; (6) Known hypersensitivity to any of the study drugs, their metabolites, or formulation excipient; (7) Previous participation in a RCT in the last 3 months.

### Randomization

After obtaining the ICF, patients were randomized using the RedCap, a secure web application for building and managing electronic case report forms (eCRF). Patients were randomly (1:1) assigned to one of the following arms with no baseline stratification:

1. Experimental arm: methylprednisolone pulses of 120 mg/day had to be administered on 3 consecutive days after randomization (if not previously administered). The administration of higher doses or longer duration of corticosteroids was allowed if their treating physicians considered it appropriate. Tacrolimus starting dose was 0.05 mg/kg (Adoport^®^) twice daily. Patients using lopinavir-ritonavir received 0.2 mg (Modigraf^®^) every 48 h. Thereafter, tacrolimus dosing was individualized through therapeutic drug monitoring to achieve blood trough levels of 8–10 ng/ml. In addition, patients in the experimental arm could receive standard of care (SoC) for their management in accordance with treating physicians.
2. Control arm (SoC): SoC included measures of supplemental oxygen and respiratory support, fluid therapy, antipyretic treatment, postural measures, low molecular weight heparins, and could also include treatments with unproved antiviral (lopinavir-ritonavir, hydroxichloroquine, etc.) or immunosuppressive (any regimen of corticosteroids, tocilizumab, anakinra, etc.) drugs, or those necessary at the discretion of the treating physician, except for cyclosporine and/or tacrolimus.

The experimental drugs were started immediately after the participants were randomly assigned to that group. The experimental treatment was discontinued after patients achieved *clinical stability*, which was defined in the *outcomes* section. Experimental treatment was also discontinued if the included patient presented a severe or potentially severe infection, required invasive mechanical ventilation, extracorporeal membrane oxygenation (ECMO), or had any serious medication-related adverse event (of special interest refractory high blood pressure, decrease of more than 50% in the GFR compared with the baseline, or ventricular tachycardia).

### Procedures

All patients were followed from day 0 through day 56 or death. The planned visits and procedures are detailed in the TACROVID trial protocol (Supplementary Table 1) (21). Follow up visits were face-to-face to evaluate disease outcomes, and data was collected using an eCRF. The Bellvitge Biomedical Research Institute (IDIBELL) Clinical Research and Clinical Trials Unit (UICEC-IDIBELL) carried out the monitoring of the trial. Regular monitoring was performed by the UICEC-IDIBELL according to the International Conference on Harmonization (ICH) good clinical practice (GCP) requirements. The UICEC-IDIBELL carried out pharmacovigilance of the trial.

### Outcomes

The primary outcome was time (days) to *clinical stability* within 56 days after randomization. *Clinical stability* was defined as fulfilling all of the following criteria for 48 consecutive hours: body temperature≤37.5°C; PaO_2_/FiO_2_ >400 and/or SpO_2_/FiO_2_ > 300; and respiratory rate ≤ 24 rpm. Treatment failures were defined as: (1) patients that did not meet criteria for clinical stability 56 days after starting treatment; (2) patients presenting a serious adverse event attributed to the experimental treatment; or (3) patients dying after being included in the clinical trial.

The secondary outcomes included the number of days until normalization of each of the clinical and analytic parameters from day 0 through day 56; the clinical status according to the eight-point ordinal scale (22) from day 0 through day 56; patients who achieved a clinical status ≤ 3 after 10 days or hospital discharge (whichever was first), and on days 28 and 56; patients who achieved *clinical stability* after 10 days or hospital discharge (whichever was first), and on days 28 and 56; value of each of the analytical parameters after 10 days or hospital discharge (whichever was first), and on days 28 and 56; number of days receiving trial experimental treatment; days until hospital discharge; number of patients and days requiring non-invasive and invasive ventilatory support devices during hospitalization; changes in blood quantitative viral load by RT-PCR before start of treatment and during follow up; changes in expanded cytokine profile before starting treatment and on days 14, 28, and 56; pulmonary parenchyma involvement using chest x-ray (CXR) pulmonary severity score (23) at baseline, and at day 56; adverse events according to their seriousness and relationship to trial experimental treatment; COVID-19 related mortality at day 28 and 56 after randomization; all-cause mortality at day 28 and 56 after randomization.

### Statistical Analysis

The intention-to-treat (ITT) population consisted of all randomized patients. We estimated that the assignment of 42 patients with 1:1 randomization would provide at least 80% power to reject the null hypothesis that the experimental and control survival curves are equal. The hazard ratio of clinical stability of control patients in relation to the patients in the experimental group was 0.52, and a median survival time in the control group of 16 days was assumed. The probability of Type I error associated with this hypothesis test was 0.05 and 5% withdrawal was anticipated.

A descriptive analysis of the baseline profile of patients included in the ITT population was carried out. The primary efficacy outcome was time (days) to *clinical stability* within 56 days after randomization, and was estimated using the Kaplan–Meier method, and cumulative incidence curves were compared between the two groups with the log-rank test. The stratified Cox proportional-hazards model was used to estimate the hazard ratio (for experimental group as compared with control) and 95% confidence interval. The main analysis was repeated on each *clinical stability* criterion. Moreover, odds of clinical stability were compared at 10, 28, and 56 days using a logistic regression.

Time to WHO eight-point ordinal scale ≤ 3 and time to death (secondary outcomes) were compared between the two groups using the Kaplan–Meier approach, and using a logistic regression at 10, 28, and 56 days. A sensitivity analysis of the time to hospital discharge was performed. Safety was assessed in all patients; patients were grouped according to the study group. A safety review was performed by the UICEC-IDIBELL.

The IDIBELL Biostatistical Unit performed the analysis and analysts were blinded to the treatment received by patients (intervention vs. usual care). R version 4.0.3 for Windows (R Foundation for Statistical Computing, http://www.r-project.org) was used for data management and analysis.

## Results

**Figure 1:**
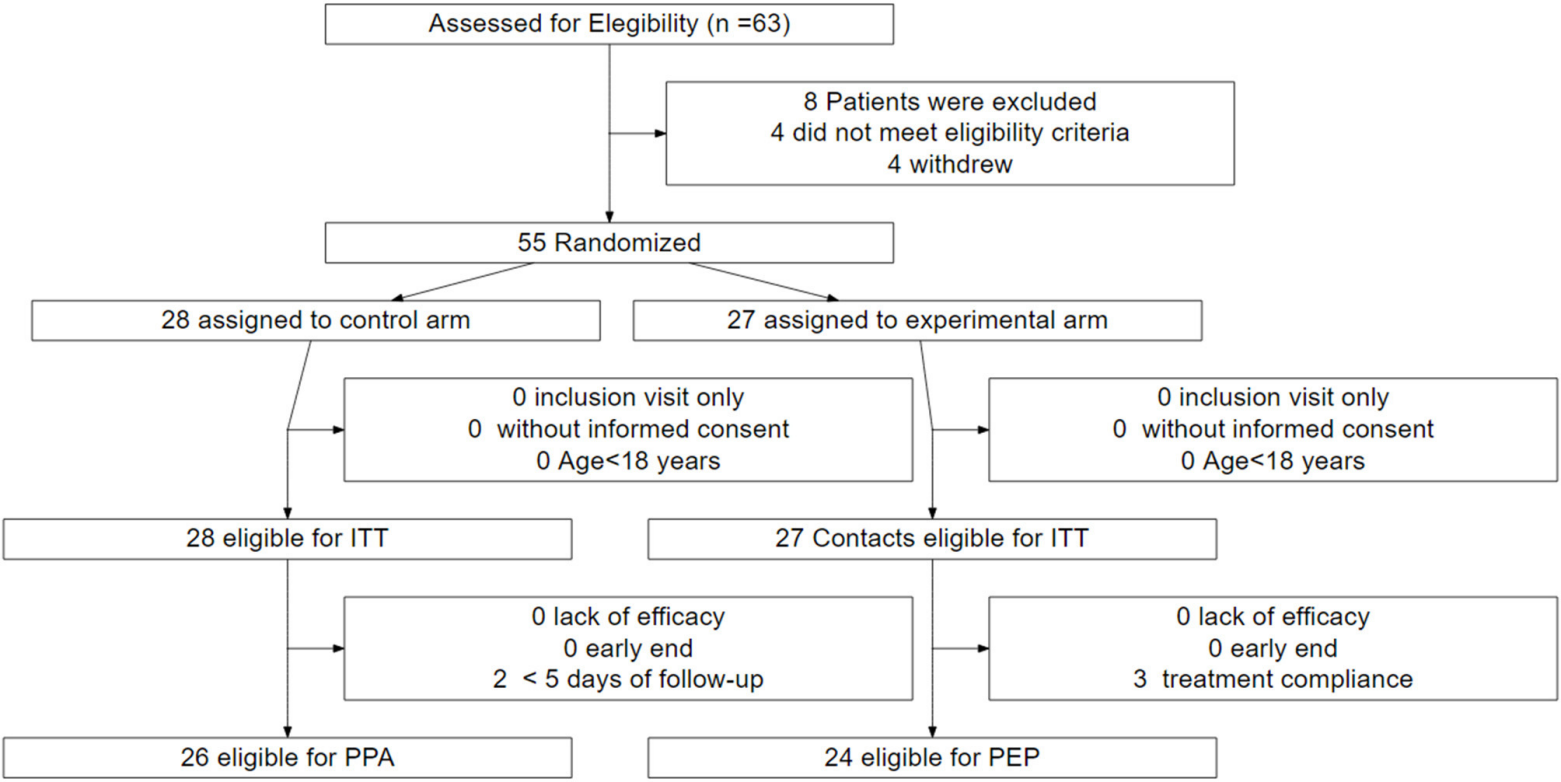
Trial profile.

Fifty-five patients were prospectively included from April 1, 2020 to May 2, 2020 in the trial for subsequent randomization. Twenty-seven were assigned to the experimental group and 28 to the control group (ITT and safety population) (Figure 1). Of those assigned to the experimental group, 24 (88.9%) patients received the treatment as assigned. Three patients discontinued the treatment during the first 5 days and were excluded from the per-protocol analysis population. Of those assigned to the control group, 26 (92.9%) were eligible for the per-protocol analysis. Two deceased patients were excluded owing to a short follow-up (<5 days).

The mean age of the 55 patients included in the ITT analysis was 63.2 (SD 13) years; 44 (80%) were male (Table 1). Overall, 39 (70.9%) of the patients were Caucasian and 16 (29.1%) were Latino. The most common pre-existing comorbidities were hypertension (43.6%), obesity (43.6%), and diabetes (27.3%). Thirty-eight (69.1%) patients had no smoking history. The median Charlson index was 3 in both groups. Except for one patient in the experimental group, all patients showed independence in tasks of daily living without cognitive impairment. There were no patients admitted from long-term care facilities or nursing homes.

**Table 1:** Baseline characteristics.

	**Experimental** **(*N* = 27)**	**Control** **(*N* = 28)**
Age (years)	61.5 (13.9)	64.8 (12.1)
Sex (male)	23 (85.2%)	21 (75%)
**Race or ethnic group**		
Caucasian	20 (74.1%)	7 (25%)
Hispanic or Latino	7 (25.9%)	9 (32.1%)
Other	0 (0%)	1 (3.6%)
**Coexisting conditions**		
Smoking history	7 (25.9%)	10 (35.7%)
Hypertension	10 (43.5%)	14 (53.8%)
Diabetes mellitus	6 (22.2%)	9 (32.1%)
Obesity	11 (40.7%)	13 (46.4%)
Coronary heart disease	3 (11.1%)	1 (3.6%)
Charlson Index	3 (1–3)	3 (2–4)
Barthel Index	100 (100–100)	100 (100–100)
Time from symptom onset to randomization, days	11 (9–17)	14 (9.75–19.3)
Early (≤10 days from symptom onset)	13 (48.1%)	10 (35.7%)
Late (>10 days from symptom onset)	14 (51.9%)	18 (64.3%)
Body temperature, °C	36.3 (0.5)	36.31 (0.53)
Respiratory rate, breaths per min	25.7 (7.8)	25.0 (4.4)
PaO_2_/FiO_2_	236.7 (220.6–261.2)	217.9 (124.2–237.5)
SpO_2_/FiO_2_	178 (160–193.7)	157.5 (106.1–165.4)
FiO_2_	0.5 (0.5–0.6)	0.6 (0.6–0.9)
Score on eight-point ordinal scale	5 (5–5)	5 (5–6)
5.Hospitalized, requiring supplemental oxygen	23 (85.2%)	16 (57.1%)
6.Hospitalized, receiving non-invasive ventilation or high-flow oxygen devices	4 (14.8%)	12 (42.9%)
PSI	77.0 (27.3)	79.4 (23.3)
NEWS	7.5 (2.1)	7.71 (1.88)
SOFA score	1 (0–1)	1 (0–1)
**Laboratory**		
Lymphocyte count, ×10^9^/L	0.72 (0.58–0.91)	0.56 (0.44–0.78)
Platelets count, ×10^9^/L	311 (115)	288 (123)
Serum creatinine, mg/dl	0.84 (0.23)	0.79 (0.24)
ALT, U/L	47.0 (32.0–70.5)	51.5 (35.8–92.2)
AST, U/L	39.0 (30.5–56.0)	41.0 (24.8–67.0)
LDH, U/L	435 (114)	450 (183)
CRP, mg/L	139.5 (24.1–195.75)	39.2 (17.3–109.9)
Ferritin, μg/L	1735.3 (1420.15–2346.15)	1695.2 (1212.1–1894.6)
IL-6, ng/L	86.1 (37.6–785)	80.4 (41.4–667)
Creatinine kinase, U/L	75.0 (43.5–198)	47.5 (31.5–65.0)
D-dimer, μg/L	612 (250–2672.5)	741 (352–2195.5)
Baseline viral load of nasopharyngeal and oropharyngeal swabs, log_10_ copies per ml	244,954 (8,566–7,458,132)	388,329 (22,551–980,683)
**Treatments at baseline**		
Lopinavir-ritonavir	23 (85.2%)	22 (78.6%)
Hydroxychloroquine	27 (100%)	28 (100%)
Antibiotic	13 (48.1%)	8 (28.6%)
Corticosteroids	10 (37.0%)	17 (60.7%)
Tocilizumab	24 (88.9%)	22 (78.6%)

*Data are median (IQR) or mean (SE), and n (%). ALT, alanine aminotransferase; AST, aspartate aminotransferase; CRP, C-reactive protein; FiO_2_, fractional inspired oxygen; IL-6, interleukin-6; LDH, lactate dehydrogenase; NEWS, National Early Warning Score; PaO_2_, arterial oxygen partial pressure; PSI, Pneumonia Severity Index; SOFA, Sequential Organ Failure Assessment; SpO_2_, oxygen saturation*.

Some imbalances existed at enrollment between the two groups. The time between symptom onset and randomization was 11 days (IQR 9–17) in the experimental group compared to 14 days (IQR 9.75–19.2) in the control group. A higher proportion of the control group had required high-flow nasal cannula or non-invasive mechanical ventilation and corticosteroids. Conversely, the experimental group showed higher CRP and creatinine kinase. No other major differences in symptoms, signs, laboratory results, disease severity, or treatments were observed between the groups at baseline (Table 1).

Patients in the experimental group received a median of 9 (IQR 7–11) days of tacrolimus, with a median time from symptom onset to tacrolimus administration of 11 days (IQR 9–17). Median tacrolimus dose per kg bodyweight was 0.0375 mg/kg twice daily (IQR 0.0276-0.05), and it was 0.0025 mg/kg every other day (IQR 0.0024-0.0029) when receiving concomitant lopinavir-ritonavir. Tacrolimus median trough levels were 8.4 ng/ml (IQR 4.6-15.1). The need for high flow devices and mechanical ventilation (invasive or not) during the follow up was similar in the two arms of the trial (Table 2). All patients received corticosteroids, with a median time from symptom onset to corticosteroid therapy of 10 days (IQR 8.00–14.0) in the experimental group and 10 days (IQR 8.75–15.0) in the control group. The dose of any type of corticosteroid received from admission to day 56 of the trial was converted to methylprednisolone, and the cumulative doses were significantly lower in the experimental group than in the control group (median methylprednisolone cumulative doses were 360 mg [IQR 360–842] vs. 870 mg [IQR 364–1451]; *p* = 0.007), as was the duration of corticosteroid treatment (median of 4.00 days [3.00–17.5] vs. 18.5 days [3.00–53.2]; *p* = 0.011). Most of the patients included also received tocilizumab (25 [92.6%] in the experimental group vs. 24 [85.7%] in the control group); *p* = 0.669). Two patients (7.14%) in the control group received anakinra. After tacrolimus initiation no patients in the experimental group received any additional immunosuppressant drug other than steroids. No significant differences were observed among the two groups in the number of patients who received lopinavir-ritonavir, hydroxychloroquine, or antibiotics. Length of oxygen support, as well as the rate and duration of ventilation support were not significantly different between the two groups (Table 2).

**Table 2:** Treatments received during hospitalization and the trial period.

	**Experimental (*N* = 27)**	**Control (*N* = 28)**	***P*-value**
Duration of oxygen support from randomization, days	11.0 (8.00–19.5)	13.0 (7.75–23.0)	0.953
High-flow or ventilatory support therapies	14 (51.9%)	18 (64.3%)	0.509
HFNC and/or non-invasive mechanical ventilation	13 (48.1%)	18 (64.3%)	0.350
Invasive mechanical ventilation	5 (18.5%)	4 (14.3%)	0.729
ECMO	0 (0%)	1 (%)	··
Duration of high-flow or ventilatory support from randomization, days	8.00 (5.00–27.2)	6.50 (4.25–14.2)	0.303
HFNC and/or non-invasive mechanical ventilation	5.00 (4.00–9.00)	5.00 (3.25–9.00)	0.532
Invasive mechanical ventilation	22.0 (11.0–29.0)	10.00 (4.25–21.5)	0.327
Renal replacement therapy	2 (7.4%)	0 (0%)	··
Corticosteroid therapy	27 (100%)	28 (100%)	··
Duration of corticosteroid therapy, days	4.00 (3.00–17.5)	18.5 (3.00–53.2)	0.011
Methylprednisolone cumulative dose	360 (360–842)	870 (364–1,451)	0.007
Tocilizumab	25 (92.6%)	24 (85.7%)	0.669
Anakinra	0 (0%)	2 (7.14%)	0.491
Lopinavir-ritonavir	23 (85.2%)	22 (78.6%)	0.729
Hydroxychloroquine	27 (100%)	28 (100%)	.
Heparin	27 (100%)	28 (100%)	.
Antibiotics	23 (85.2%)	26 (92.9%)	0.422

*Data are n (%) compared using Pearson's chi-squared test, and median (interquartile range) compared using the Wilcoxon test. ECMO, extracorporeal membrane oxygenation; HFNC, high-flow nasal cannula*.

**Figure 2:**
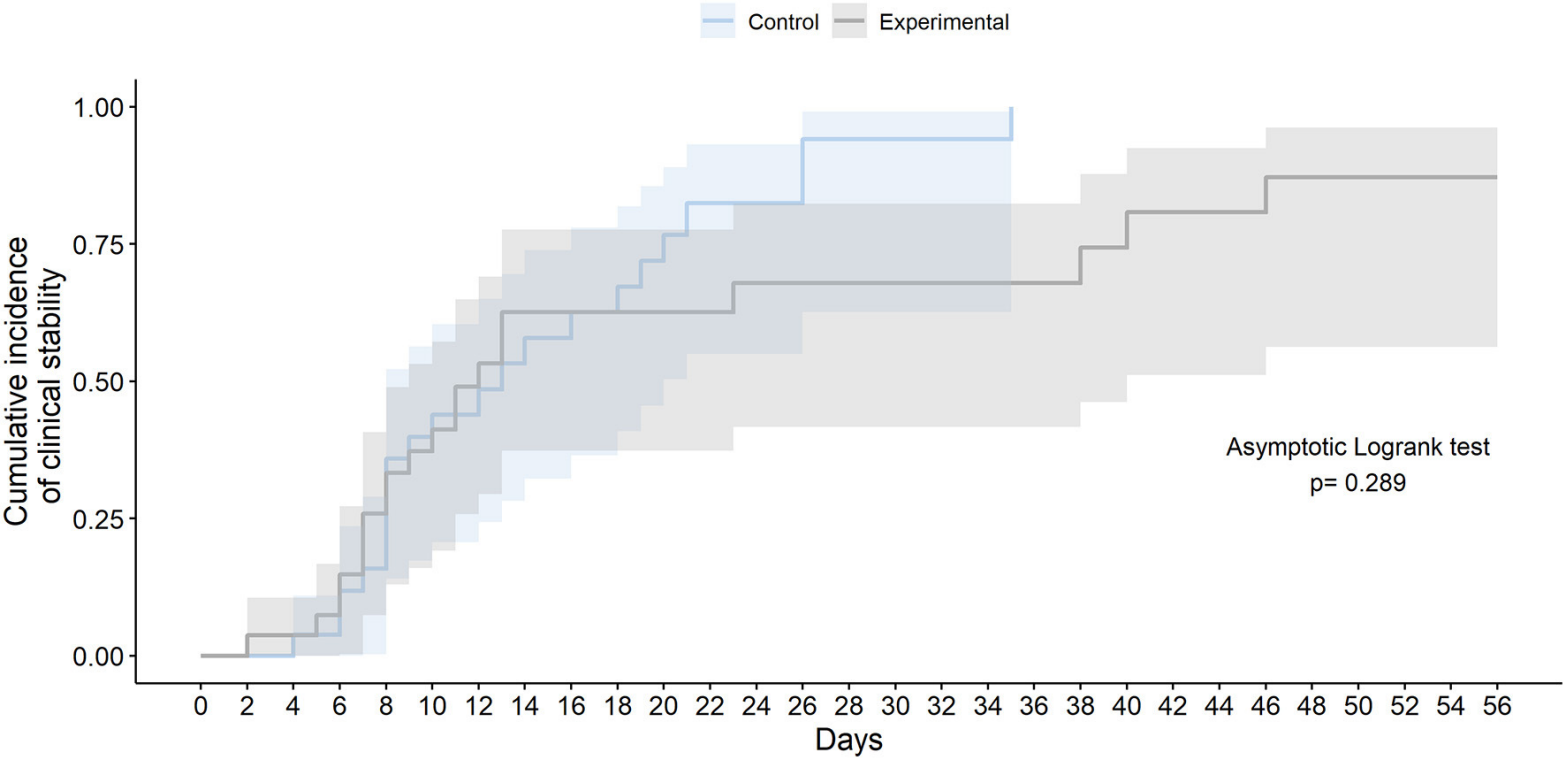
Time to clinical stability in the intention-to-treat population.

The final study follow-up was on June 27, 2020. In the ITT population, no statistically significant differences were observed in time to *clinical stability* within 56 days after randomization between the two groups (median 10.0 days [IQR 7.0–13.0] in the experimental group vs. 11.0 days [8.0–18.0] in the SoC group; HR 0.73 [95% CI 0.39–1.37]; Asymptotic Logrank test *p*-value = 0.325) (Table 3 and Figure 2). The times to normalization of each of the variables that compound *clinical stability* (body temperature, PaO_2_/FiO_2_ or SpO_2_/FiO_2_; and respiratory rate) did not differ significantly between arms. Results for time to *clinical stability* were similar in the per-protocol population (median 10.0 days [IQR 7.00–12.5] in the experimental group vs. 11.0 days [IQR 8.0–18.8] in the SoC group; HR 0.77 [95% CI 0.40–1.47]; Asymptotic Logrank test *p*-value = 0.473). At 56 days after randomization, the number of patients who had achieved *clinical stability*, those with an eight-point ordinal scale ≤ 3, and patients discharged did not differ significantly between the groups (Table 3).

**Table 3:** Effect of allocation to experimental group on key study outcomes.

	**Experimental (*N* = 27)**	**Control (*N* = 28)**	**HR/OR [CI 95%]**	***P*-value**
Time to clinical stability, days	10.0 (7.00–13.0)	11.0 (8.00–18.8)	0.73 [0.39–1.37]	0.327^∧^
Time to body temperature normalization, days	1.00 (1.00–2.00)	1.00 (1.00–1.00)	0.8 [0.47–1.36]	0.415^∧^
Time to PaO_2_/FiO_2_ > 400 and/or SpO_2_/FiO_2_ > 300	9.00 (7.00–11.0)	11.0 (8.00–18.8)	0.81 [0.43–1.53]	0.525^∧^
Time to respiratory rate < 24 bpm	5.00 (2.00–9.00)	5.00 (3.00–7.00)	1.03 (0.59–1.81)	0.909^∧^
**Patients who achieved clinical stability**				
at day 10	11 (40.7%)	11 (39.3%)	1.06 [0.35–3.19]	0.915^*^
at day 28	18 (66.7%)	21 (75.0%)	0.67 [0.20–2.21]	0.515^*^
at day 56	21 (77.8%)	22 (78.6%)	0.96 [0.25–3.61]	0.946^*^
Time to an eight-point ordinal scale ≤ 3	12.5 (8.00–15.2)	15.0 (9.00–24.0)	0.92 [0.49–1.71]	0.787^∧^
**Patients who reach an eight-point ordinal scale** **≤** **3**				
at day 10	9 (33.3%)	7 (25.0%)	1.48 [0.45–5.03]	0.515^*^
at day 28	18 (66.7%)	20 (71.4%)	0.80 [0.25–2.59]	0.714^*^
at day 56	20 (74.1%)	21 (75.0%)	0.95 [0.27–3.33]	0.940^*^
Discharge at day 56	21 (77.8%)	21 (75.0%)	1.16 [0.32–4.28]	0.819^*^
Duration of hospital stay, days	13.0 (8.50–21.0)	14.0 (9.00–22.5)	.	0.933^**^
**COVID-19-related mortality**				
at day 10	2 (7.41%)	3 (10.7%)	0.69 [0.07–4.88]	0.705^*^
at day 28	3 (11.1%)	4 (14.3%)	0.76 [0.13–4.02]	0.747^*^
at day 56	4 (14.8%)	4 (14.3%)	1.04 [0.21–5.13]	0.958^*^
Time from randomization to COVID-19-related death, days	18.0 (9.50–27.8)	7.00 (3.50–12.5)	0.96 [0.24–3.84]	0.953^∧^
**All-cause mortality**				
at day 10	2 (7.41%)	5 (17.9%)	0.39 [0.05–2.10]	0.282^*^
at day 28	4 (14.8%)	6 (21.4%)	0.65 [0.14–2.67]	0.551^*^
at day 56	5 (18.5%)	6 (21.4%)	0.84 [0.21–3.28]	0.800^*^
Time from randomization to all-cause death, days	13.0 (10.0–26.0)	7.00 (3.25–10.0)	0.80 [0.24–2.61]	0.707^∧^

*Data are median (IQR) or n (%). COVID-19, Coronavirus disease 2019; FiO2, fractional inspired oxygen; OS, ordinal scale; PaO_2_, arterial oxygen partial pressure; SpO_2_, oxygen saturation. Clinical stability (the event) was defined as fulfilling all of the following criteria for 48 consecutive hours: body temperature ≤ 37.5°C; PaO_2_/FiO_2_ > 400 and/or SpO_2_/FiO_2_ > 300; and respiratory rate ≤ 24 breaths per minute (bpm). Treatment failure was defined as: (1) patient who does not meet the criteria for clinical stability 56 days after starting treatment; (2) patient presenting a serious adverse event attributed to trial treatment; or (3) patient who dies after being included in the clinical trial. Differences are expressed as*

∧ *Hazard Ratio [CI 95%] and p-value*;

* *Odds ratio [CI 95%] and p-value*;

** *Wilcoxon test and p-value*.

**Figure 3:**
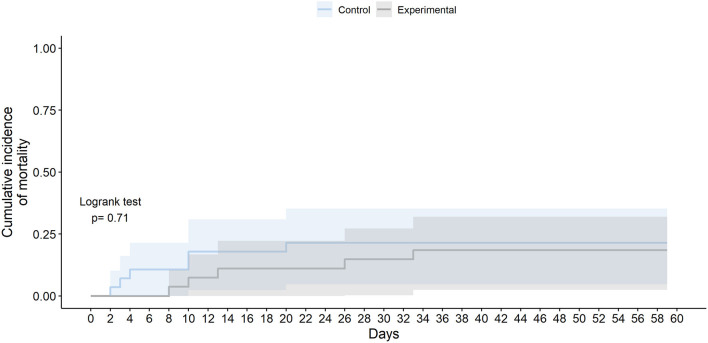
All cause mortality in the ITT population.

Although not statistically significant, those receiving the experimental therapy showed a numerically lower all-cause mortality than those receiving SoC, especially at day 10 (2 [7.41%] vs. 5 [17.9%]; OR 0.39 [95% CI 0.05–2.1]; *p* = 0.282) and at day 28 (4 [14.8%] vs. 6 [21.4%]; OR 0.65 [95% CI 0.14–2.67]; *p* = 0.551) (Figure 3). Patients in the experimental group died later at a median of 13 days from randomization (IQR 10.0–26.0), while in the control group the median was 7 days (3.25–10.0); but these differences were not statistically significant (logrank test *p*-value 0.710). The number of available events by group (four deaths per study arm) was not enough to get reliable estimators to analyze the effect of experimental therapy on all-cause mortality adjusting by age or sex. Similar results were obtained for COVID-19-related mortality (Table 3) (Supplementary Figure 1).

There were no significant differences in the evolution of analytic parameters (lymphocytes, CRP, ferritin, LDH, IL-6, D-dimer) between the two arms (Supplementary Table 2), or in the expanded cytokine profile (Supplementary Table 3). Serum cytokine levels at different time points during the trial showed increased levels of pro-inflammatory cytokines like CXCL10 (IP-10), IL-6, IL-18, TNF-alpha, and soluble IL-2 receptor alpha; and regulatory cytokines such as IL-10 and IL-1RA at day 0 and day 14. In both groups, serum cytokine levels tended to have decreased by day 28 and day 56 (Supplementary Figure 2). In the same way, there were no significant differences between groups in pulmonary parenchyma involvement according to the CXR pulmonary severity score at inclusion or at day 56 (Supplementary Table 4).

All 55 patients were nasopharyngeal and oropharyngeal RT-PCR positive at diagnosis, but viral load data was available in 24 (88.8%) patients in the experimental group and 20 (71.4%) in the control group. The median baseline viral load of upper respiratory tract swabs was 244,954 (IQR 8,566–7,458,132) log_10_ copies per ml in the experimental group and 388,329 (IQR 22,551–980,683) log_10_ copies per ml in the control group. During follow-up, upper respiratory tract viral load decreased over time similarly in both arms, becoming undetectable at day 28 and 56 in most patients. Blood RT-PCR at baseline was available in 24 (88.8%) patients in the experimental group and 21 (75%) in the control group. Almost all of them showed undetectable viral RNA in blood samples at baseline and during follow-up (Supplementary Table 5 and Supplementary Figure 3).

Adverse events (AE) occurred in 46 (83.6%) patients. Twenty-two (44%) patients had one AE, five (9.1%) had two AE, 11 (20%) had three AE, and eight (14.5%) had four or more AE. Sixty-two AE were reported in 23 (85.2%) patients in the experimental group, 20 of them met the seriousness criteria (corresponding to nine patients), and nine were assessed as related to the experimental treatment. In the control group, 55 AE were reported in 23 (82.1%) patients, of which 13 were considered as serious AE (corresponding to 10 patients) (Supplementary Table 6).

The total number of non-serious AE was 42 in each of the two groups. Those receiving experimental treatment had a numerically higher rate of non-serious infectious AE (16 [38%] vs. 10 [24%]), and serious infectious AE (7 [35%] vs. 3 [23%]) than those receiving SoC. In contrast, the control group showed poorer glucose metabolism and a higher overall bleeding rate. Five (18.5%) patients in the experimental group developed special interest AE, these being hypertension in three (60%) of them and renal impairment in two (40%). Four deaths in each group were judged by the site investigators to be related to COVID-19 acute respiratory distress syndrome. One death reported in the experimental group was attributed to hemorrhagic stroke, and regarding the two deaths in the control group, one was attributed to *Staphylococcus aureus* septicaemia and the other to hemopericardium (Supplementary Table 6).

## Discussion

The TACROVID trial found that methylprednisolone bolus plus tacrolimus did not significantly improve the time to *clinical stability* (primary outcome), mortality or other secondary outcomes compared with the SoC in hospitalized patients with severe COVID-19. Furthermore, no differences were observed in the clearance of the virus or in the rate of adverse events between the two groups.

The TACROVID trial was initiated in March 29, 2020 when there were no medical reports supporting the use of immunosuppressive therapy in severe COVID-19. Nonetheless, all of the trial's patients received corticosteroids. Methylprednisolone and tacrolimus lead to impaired lymphocyte function (7, 13) and therefore it could facilitate SARS-CoV-2 replication and also promote the development of other infections. On the other hand, CNIs have been shown to inhibit the growth of human coronaviruses at low micromolar, non-cytotoxic concentrations in cell cultures by immunophilin pathway inhibition (16, 17). Based on this finding, it has been suggested that CNIs could be used as an antiviral agent to treat COVID-19. However, we would like to highlight that the concentrations used in cell culture are not clinically achievable, as they correspond to highly toxic blood levels in humans (24). Accordingly, the proposed use of tacrolimus should be restricted to the inflammatory stages of COVID-19. In this trial, tacrolimus had no significant effect on SARS-CoV-2 RNA loads either in the upper respiratory tract or in blood specimens in our patients.

The ratio of most treatments (antibiotics, lopinavir-ritonavir, hydroxychloquine, heparin, and tocilizumab) used was similar in the two groups. Interestingly, the largest and longest corticosteroids doses were used in the control group, although we do not know the exact reasons. During follow up both groups had similar laboratory test results, needed similar rates of high-flow and ventilation devices, and developed similar CXR parenchymal involvement. Notably, those patients receiving the experimental therapy had a numerically shorter time to achieve an eight-point ordinal scale ≤ 3 than those receiving SoC within 10 days of randomization, although this trend was later reversed. Finally, this was an open-label trial and the control group patients who could not receive tacrolimus may have received more corticosteroids or other immunosuppressants (anakinra).

Although not statistically significant, patients receiving the experimental therapy had numerically lower all-cause mortality than those receiving SoC, especially during the first 28 days. This data supports non-randomized studies that showed that cyclosporine could reduce mortality, mainly in patients with moderate to severe COVID-19 (19, 20). Interestingly, tacrolimus use had a positive independent effect on survival vs. all other immunosuppressant (cyclosporine, mycophenolate and/or mTOR inhibitors) according to a multi-center European study carried out on 243 adult liver transplant recipients with symptomatic COVID-19, suggesting that it could be even more beneficial than cyclosporine (25). The mortality data from days 28 to 56 of the trial are less valuable because only five patients were still admitted to the hospital in the experimental group (Supplementary Table 7), and experimental therapy was withdrawn previously in all of them due to mechanical invasive ventilation or serious AE.

Likewise, there was no difference in adverse events overall between groups. Patients in the experimental group seemed to have a slightly higher number of non-serious and serious AE infections. We also have to consider that corticosteroids have been associated with gastrointestinal bleeding, hyperglycaemia, and neuromuscular weakness (26). In fact, the group treated with tacrolimus received a significantly lower dose of corticosteroids, having better control of glucose metabolism and a lower rate of bleeding.

However, the trial had some limitations. First, the current trial was not conducted as a double-blind trial. This was considered unrealistic given the intense workload experienced at the beginning of the pandemic in our local setting. To minimize the impact of an open-label design, the statistician performing the analysis was blinded to the trial arm. Second, the TACROVID trial had a limited sample size and clearly was not sufficiently powered to detect a difference in time to *clinical stability* and mortality between the two groups after the early termination that occurred with 29 (34.1%) patients fewer than expected. Furthermore, the limited sample size caused certain imbalances in the baseline characteristics between the two groups after randomization. Third, all included patients received corticosteroids, heparins, and hydroxychloroquine; and most (89.1%) of them also received tocilizumab as part of the SoC. In this respect, the additional use of any other medication regimens (except for cyclosporine) in both arms, as part of the SoC, limits the assessment of which was the real effect of each drug on clinical outcomes, laboratory data and the occurrence of AE. Moreover, tacrolimus strongly interacts with some treatments (especially lopinavir) used at that time in COVID-19. Most (85.2%) patients in the experimental group received concomitant treatment with lopinavir-ritonavir, as it was extremely difficult to achieve the recommended plasma levels of tacrolimus. Finally, the lack of medical evidence supporting immunosuppressive therapies in COVID-19, when the trial was conducted, made us more cautious, withdrawing experimental therapy when mechanical invasive ventilation was implemented. Therefore, its efficacy and safety cannot be assessed by this trial in this subset of patients with life-threatening COVID-19.

In summary, the combined use of methylprednisolone pulses and tacrolimus, in addition to the SoC did not significantly improve the time to *clinical stability* or other secondary outcomes compared with SoC alone in hospitalized patients with severe COVID-19. Although not statistically significant, patients receiving the experimental therapy had numerically lower all-cause mortality than those receiving SoC. No relevant differences were observed in the clearance of the virus or in the rate of adverse events between the two groups. The reason why the largest and longest corticosteroid doses were used in the control group remains unclear. It is noteworthy that the present trial had a limited sample size and several other limitations. Therefore, further RCTs should be done to assess the efficacy and safety of tacrolimus to tackle the inflammatory stages of COVID-19.

## Data Availability Statement

The raw data supporting the conclusions of this article will be made available by the authors, without undue reservation.

## Ethics Statement

The studies involving human participants were reviewed and approved by Bellvitge University Hospital's Ethical Committee for Drug Research. The patients/participants provided their written informed consent to participate in this study.

## Author Contributions

XS, AA, GR-B, CT, and XC had full access to all of the data in the study and take responsibility for the integrity of the data and the accuracy of the data analysis. XS, AA, AR-M, CT, SV, PH, and XC provided input on the trial design. XS, AA, GR-B, AR-M, CT, and XC were responsible for the acquisition, analysis, and interpretation of data. XS, AA, GR-B, AR-M, CT, NP, and XC drafted the manuscript. MF-M, AI, FM, OC, JB, AM-V, SV, and PH critically revised the manuscript. CT contributed to the statistical analysis. XS and XC verified the underlying data. All authors contributed to conducting the trial, read, and approved the final manuscript.

## Conflict of Interest

The authors declare that the research was conducted in the absence of any commercial or financial relationships that could be construed as a potential conflict of interest.

## Abbreviations

AE: adverse event
ALT: alanine aminotransferase
ARDS: acute respiratory disease syndrome
HUB: Hospital Universitari de Bellvitge
CNIs: calcineurin inhibitors
CoV: Coronavirus
COVID-19: Coronavirus disease 2019
CRP: C-reactive protein
CXCL: chemokine (C-X-C motif) ligand
CXR: chest x-ray
ECMO: extracorporeal membrane oxygenation
eCRF: electronic case report form
FiO_2_: fractional inspired oxygen
G-CSF: granulocyte colony-stimulating factor
GFR: glomerular filtration rate
ICF: informed consent form
IFN: interferon
IL: interleukin
ITT: intention to treat
LDH: lactate dehydrogenase
MCP: monocyte chemoattractant protein
PaO_2_: arterial oxygen partial pressure
RCT: randomized controlled trial
RT-PCR: reverse transcription polymerase chain reaction
SAE: serious adverse event
SARS-CoV: Severe Acute Respiratory Syndrome Coronavirus
SARS-CoV-2: Severe Acute Respiratory Syndrome Coronavirus 2
SF: SpO_2_/FiO_2_ ratio
SoC: Standard of Care
SOFA score: Sequential Organ Failure Assessment Score
SpO_2_: oxygen saturation
TNF: tumor necrosis factor.

## References

[1] Lai CC Shih TP Ko WC Tang HJ Hsueh PR. Severe acute respiratory syndrome coronavirus 2 (SARS-CoV-2) and coronavirus disease-2019 (COVID-19): the epidemic and the challenges. Int J Antimicrob Agents. (2020) 55:105924. 10.1016/j.ijantimicag.2020.105924 32081636 PMC7127800

[2] Wang L Wang Y Ye D. Review of the 2019 novel coronavirus (SARS-CoV-2) based on current evidence. Int J Antimicrob Agents. (2020) 55:105948. 10.1016/j.ijantimicag.2020.105948 32201353 PMC7156162

[3] WHO. Director-General's Opening Remarks at the Media Briefing on COVID-19. (2020). Available online at: https://www.who.int/dg/speeches/detail/who-director-general-s-opening-remarks-at-the-media-briefing-on-covid-19 (accessed March 11, 2020).

[4] Hadjadj J Yatim N Barnabei L Corneau A Boussier J Smith N. Impaired type I interferon activity and inflammatory responses in severe COVID-19 patients. Science. (2020) 369:718-24. 10.1126/science.abc6027 32661059 PMC7402632

[5] Vabret N Britton GJ Gruber C Hegde S Kim J Kuksin M. Immunology of COVID-19: current state of the science. Immunity. (2020) 52:910-41. 10.1016/j.immuni.2020.05.002 32505227 PMC7200337

[6] Siddiqi HK Mehra MR. COVID-19 illness in native and immunosuppressed states: a clinical-therapeutic staging proposal. J Heart Lung Transplant. (2020) 39:405-7. 10.1016/j.healun.2020.03.012 32362390 PMC7118652

[7] Russell B Moss C George G Santaolalla A Cope A Papa S. Associations between immune-suppressive and stimulating drugs and novel COVID-19-a systematic review of current evidence. Ecancermedicalscience. (2020) 14:1022. 10.3332/ecancer.2020.1022 32256705 PMC7105343

[8] RECOVERY Collaborative Group Horby P Lim WS Emberson JR Mafham M Bell JL. Dexamethasone in hospitalized patients with Covid-19 - preliminary report. N Engl J Med. (2021) 384:693–704. 10.1056/NEJMoa2021436 32678530 PMC7383595

[9] Kalil AC Patterson TF Mehta AK Tomashek KM Wolfe CR Ghazaryan V. Baricitinib plus Remdesivir for hospitalized adults with Covid-19. N Engl J Med. (2021) 384:795-807. 10.1056/NEJMoa2031994 33306283 PMC7745180

[10] Wang Y Zhang D Du G Du R Zhao J Jin Y. Remdesivir in adults with severe COVID-19: a randomised, double-blind, placebo-controlled, multicentre trial. Lancet. (2020) 395:1569–78. 10.1016/S0140-6736(20)31022-9 32423584 PMC7190303

[11] Colson P Rolain JM Lagier JC Brouqui P Raoult D. Chloroquine and hydroxychloroquine as available weapons to fight COVID-19. Int J Antimicrob Agents. (2020) 55:105932. 10.1016/j.ijantimicag.2020.105932 32145363 PMC7135139

[12] Henderson LA Canna SW Schulert GS Volpi S Lee PY Kernan KF. On the alert for cytokine storm: immunopathology in COVID-19. Arthritis Rheumatol. (2020) 72:1059–63. 10.1002/art.41285 32293098 PMC7262347

[13] Hirano K Ichikawa T Nakao K Matsumoto A Miyaaki H Shibata H. Differential effects of calcineurin inhibitors, tacrolimus and cyclosporin a, on interferon-induced antiviral protein in human hepatocyte cells. Liver Transpl. (2008) 14:292-8. 10.1002/lt.21358 18306331

[14] Mehta P McAuley DF Brown M Sanchez E Tattersall RS Manson JJ. COVID-19: consider cytokine storm syndromes and immunosuppression. Lancet. (2020) 395:1033-4. 10.1016/S0140-6736(20)30628-0 32192578 PMC7270045

[15] Ramos-Casals M Brito-Zerón P López-Guillermo A Khamashta MA Bosch X. Adult haemophagocytic syndrome. Lancet. (2014) 383:1503-16. 10.1016/S0140-6736(13)61048-X 24290661

[16] Pfefferle S Schöpf J Kögl M Friedel CC Müller MA Carbajo-Lozoya J. The SARS-coronavirus-host interactome: identification of cyclophilins as target for pan-coronavirus inhibitors. PLoS Pathog. (2011) 7:e1002331. 10.1371/journal.ppat.1002331 22046132 PMC3203193

[17] Carbajo-Lozoya J Müller MA Kallies S Thiel V Drosten C von Brunn A. Replication of human coronaviruses SARS-CoV, HCoV-NL63 and HCoV-229E is inhibited by the drug FK506. Virus Res. (2012) 165:112-7. 10.1016/j.virusres.2012.02.002 22349148 PMC7114512

[18] Sanchez-Pernaute O Romero-Bueno FI Selva-O'Callaghan A. Why choose cyclosporin A as first-line therapy in COVID-19 pneumonia. Reumatol Clin. (2020). 10.1016/j.reuma.2020.03.001. [Epub ahead of print]. 32354685 PMC7160056

[19] Gálvez-Romero JL Palmeros-Rojas O Real-Ramírez FA Sánchez-Romero S Tome-Maxil R Ramírez-Sandoval MP. Cyclosporine A plus low-dose steroid treatment in COVID-19 improves clinical outcomes in patients with moderate to severe disease. A pilot study. J Intern Med. (2021) 289:906–20. 10.1111/joim.13223 33274479 PMC7753398

[20] Guisado-Vasco P Valderas-Ortega S Carralón-González MM Roda-Santacruz A González-Cortijo L Sotres-Fernández G. Clinical characteristics and outcomes among hospitalized adults with severe COVID-19 admitted to a tertiary medical center and receiving antiviral, antimalarials, glucocorticoids, or immunomodulation with tocilizumab or cyclosporine: a retrospective observational study (COQUIMA cohort). EClinicalMedicine. (2020) 28:100591. 10.1016/j.eclinm.2020.100591 33078138 PMC7557296

[21] Solanich X Antolí A Padullés N Fanlo-Maresma M Iriarte A Mitjavila F. Pragmatic, open-label, single-center, randomized, phase II clinical trial to evaluate the efficacy and safety of methylprednisolone pulses and tacrolimus in patients with severe pneumonia secondary to COVID-19: the TACROVID trial protocol. Contemp Clin Trials Commun. (2021) 21:100716. 10.1016/j.conctc.2021.100716 33495742 PMC7817439

[22] Beigel JH Tomashek KM Dodd LE Mehta AK Zingman BS Kalil AC. Remdesivir for the treatment of Covid-19 - final report. N Engl J Med. (2020) 383:1813-26. 10.1056/NEJMoa2007764 32445440 PMC7262788

[23] Monaco CG Zaottini F Schiaffino S Villa A Della Pepa G Carbonaro LA. Chest x-ray severity score in COVID-19 patients on emergency department admission: a two-centre study. Eur Radiol Exp. (2020) 4:68. 10.1186/s41747-020-00195-w 33319321 PMC7735892

[24] Solanich X Padullés N Niubó J Videla S Antolí A Rocamora-Blanch G. Inhibition of SARS-CoV-2 replication using calcineurin inhibitors: are concentrations required clinically achievable? J Intern Med. (2021) 289:926-7. 10.1111/joim.13264 33634528 PMC8013211

[25] Belli LS Fondevila C Cortesi PA Conti S Karam V Adam R. Protective role of tacrolimus, deleterious role of age and comorbidities in liver transplant recipients with Covid-19: results from the ELITA/ELTR multi-center European study. Gastroenterology. (2021) 160:1151-63.e3. 10.1053/j.gastro.2020.11.045 33307029 PMC7724463

[26] Yao TC Huang YW Chang SM Tsai SY Wu AC Tsai HJ. Association between oral corticosteroid bursts and severe adverse events: a nationwide population-based cohort study. Ann Intern Med. (2020) 173:325-30. 10.7326/M20-0432 32628532

