# Supplementary material for: Methylprednisolone Pulses Plus Tacrolimus in Addition to Standard of Care vs. Standard of Care Alone in Patients With Severe COVID-19. A Randomized Controlled Trial
Source: Front Med (Lausanne). 2021 Jun 14;8:691712. doi: 10.3389/fmed.2021.691712 (PMC8236585; doi:10.3389/fmed.2021.691712)
Supplement: Supplementary file 1 [file Data_Sheet_1.DOCX]

**TITLE:**

Methylprednisolone plus tacrolimus in patients with severe COVID-19. A randomized controlled trial

**COLLABORATORS / TACROVID STUDY GROUP**

| **Name** | **Department** |
| --- | --- |
| 1. Montero | Department of Internal Medicine, BUH-IDIBELL |
| M. Rubio-Rivas | Department of Internal Medicine, BUH-IDIBELL |
| JM. Mora-Lujan | Department of Internal Medicine, BUH-IDIBELL |
| R. Moreno | Department of Internal Medicine, BUH-IDIBELL |
| V. Esteve | Department of Internal Medicine, BUH-IDIBELL |
| C. Verge | Department of Internal Medicine, BUH-IDIBELL |
| K. Lopez-Aldabe | Department of Internal Medicine, BUH-IDIBELL |
| R. Torres | Department of Internal Medicine, BUH-IDIBELL |
| F. Escrihuela | Department of Internal Medicine, BUH-IDIBELL |
| M. Ras | Department of Internal Medicine, BUH-IDIBELL |
| A. Bergas | Department of Internal Medicine, BUH-IDIBELL |
| B. Villanueva | Department of Internal Medicine, BUH-IDIBELL |
| M. Tuells | Department of Internal Medicine, BUH-IDIBELL |
| N. Homs | Department of Internal Medicine, BUH-IDIBELL |
| L. Hidalgo | Department of Internal Medicine, BUH-IDIBELL |
| M. Molina-Molina | Department of Respiratory Medicine, BUH-IDIBELL |
| J. Ribas | Department of Respiratory Medicine, BUH-IDIBELL |
| M. Diez-Ferrer | Department of Respiratory Medicine, BUH-IDIBELL |
| Y. Ruiz-Albert | Department of Respiratory Medicine, BUH-IDIBELL |
| E. Cuevas | Department of Respiratory Medicine, BUH-IDIBELL |
| J. Bordas | Department of Respiratory Medicine, BUH-IDIBELL |
| J. Sabater-Riera | Intensive Care Department, BUH-IDIBELL |
| XL. Pérez-Fernández | Intensive Care Department, BUH-IDIBELL |
| M. Bonfill | Department of Dermatology, BUH-IDIBELL |
| S. Lakis | Department of Psychiatry, BUH-IDIBELL |
| L. Farran | Department of Rheumatology, BUH-IDIBELL |
| F. Climent | Department of Pathology, BUH-IDIBELL Biobank |
| N. Becerril | Department of Gastroenterology, BUH-IDIBELL |
| E. Baena | Department of General and Digestive Surgery, BUH-IDIBELL |
| L. Calatayud | Department of Microbiology, BUH-IDIBELL |
| J. Peñafiel | Department of Biostatistics, IDIBELL |
| N. Pallares | Department of Biostatistics, IDIBELL |

Bellvitge University Hospital (BUH), Bellvitge Biomedical Research Institute (IDIBELL),

L'Hospitalet de Llobregat, Barcelona, Spain.
