# Supplementary material for: Methylprednisolone Pulses Plus Tacrolimus in Addition to Standard of Care vs. Standard of Care Alone in Patients With Severe COVID-19. A Randomized Controlled Trial
Source: Front Med (Lausanne). 2021 Jun 14;8:691712. doi: 10.3389/fmed.2021.691712 (PMC8236585; doi:10.3389/fmed.2021.691712)
Supplement: Supplementary file 2 [file Data_Sheet_2.PDF]

PROTOCOLO DE ENSAYO CLÍNICO

ENSAYO CLÍNICO DE FASE II, PRAGMÁTICO, CON ASIGNACIÓN ALEATORIA, CONTROLADO, ABIERTO Y UNICÉNTRICO PARA EVALUAR PULSOS DE METILPREDNISOLONA Y TACROLIMUS EN PACIENTES HOSPITALIZADOS CON NEUMONIA GRAVE SECUNDARIA A COVID-19 (TACROVID)

PRAGMATIC, CONTROLLED, OPEN, SINGLE CENTER, RANDOMIZED, PHASE II CLINICAL TRIAL TO EVALUATE METHYLPREDNISOLONE PULSES AND TACROLIMUS IN HOSPITALIZED PATIENTS WITH SEVERE PNEUMONIA SECONDARY TO COVID-19 (TACROVID)

Producto experimental: Tratamiento inmunosupresor

Promotor:
Dr. Xavier SOLANICH
Servicio de Medicina Interna
Hospital Universitario de Bellvitge
Carrer de la Feixa Llarga, s/n,
08907-L'Hospitalet de Llobregat, Barcelona
Tel.: +34. 93 2602324

Código de Protocolo: TACRO-BELL-COVID.
Código EudraCT: 2020-001445-39.
Fase de desarrollo: II

Bellvitge
Hospital Universitari

Investigador Coordinador:
Dr. Xavier SOLANICH
Servicio de Medicina Interna
Hospital Universitario de Bellvitge
Carrer de la Feixa Llarga, s/n,
08907-L'Hospitalet de Llobregat, Barcelona
Tel.: +34. 93 2602324

Monitor Médico:
Dr. Arnau ANTOLÍ
Servicio de Medicina Interna
Hospital Universitario de Bellvitge
Carrer de la Feixa Llarga, s/n,
08907-L'Hospitalet de Llobregat, Barcelona
Tel.: +34. 93 2602324

Proyecto de investigación en salud
IP Dr. Xavier Solanich

La información contenida en el presente documento es estrictamente confidencial y sólo puede ser utilizada para su revisión por parte de los Investigadores, los Comités de Ética de la Investigación y las Autoridades competentes. Queda prohibida la reproducción, transmisión o copia, en cualquier soporte, total o parcial, sin la autorización previa del Promotor o un representante autorizado.

Versión 5.0 del 28 de Abril de 2020

FIRMAS

Investigador Coordinador:

He leído y estoy de acuerdo con el protocolo titulado *Ensayo clínico de Fase II, pragmático, con asignación aleatoria, controlado, abierto y unicéntrico para evaluar pulsos de metilprednisolona y tacrolimus en pacientes hospitalizados con neumonía grave secundaria a COVID-19 (TACRO-BELL-COVID)*

Estoy informado de mis responsabilidades como *Investigador Principal* bajo las normas de Buena Práctica Clínica (BPC), la legislación local y del protocolo del estudio. Me comprometo a realizar el estudio de acuerdo con estas normas, y a dirigir y ayudar adecuadamente al equipo bajo mi cargo que realizará el estudio.

Promotor

__________________________________ ____________________

Firma: Dr. Xavier Solanich Fecha

Organización de Investigación por Contrato (CRO): UICEC-Idibell

__________________________________ ____________________

Firma: Dra. Pilar Hereu Boher Fecha

1. RESUMEN

**Nombre del promotor:**<br>Dr. Xavier SOLANICH<br>**Nombre del Producto Terminado:**<br>- Urbason®, Solu-Moderín®<br>- Advagraf®, Modigraf®<br>**Nombre del Principio Activo:**<br>Metilprednisolona / Tacrolimus	**Para Uso Exclusivo de las Autoridades Sanitarias**

TÍTULO DEL ESTUDIO: Ensayo clínico de Fase II, pragmático, con asignación aleatoria, controlado, abierto y unicéntrico para evaluar pulsos de metilprednisolona y tacrolimus en pacientes hospitalizados con neumonía grave secundaria a COVID-19 (TACRO-BELL-COVID)

FASE DE DESARROLLO: II (ensayo clínico con tratamientos inmunosupresores comercializados fuera de ficha técnica).

JUSTIFICACIÓN GENERAL:
El tratamiento de la enfermedad por COVID-19 se basa en las terapias de soporte vital. No existe aún evidencia científica procedente de ensayos clínicos sobre la eficacia ni seguridad de fármacos para tratar la enfermedad. La urgencia asistencial hace que, a pesar de la falta de evidencia, se utilicen ya algunos fármacos en la práctica diaria. Fundamentalmente, se están realizando ensayos con para detener la replicación del virus mediante la combinación de inhibidores de la proteasa (Lopinavir / ritonavir) con o sin interferón β, hidroxicloroquina, etc.

La principal causa de mortalidad es la insuficiencia respiratoria secundaria al síndrome de dificultad respiratoria aguda (Wang D, JAMA 2020; Wu C, JAMA Intern Med; Ruan Q, Intensive Care Med 2020) desencadenada por la infección viral. Intervienen en esta respuesta tanto el sistema inmune innato como el adaptativo. Este proceso conduce a la activación y diferenciación de las células T, seguida de la liberación de IFNs de tipo 1 y citocinas proinflamatorias (IL-1, IL-6, IL-8, IL-21, TNF-β y MCP-1). Así pues, las interacciones virus-célula conducen a una fuerte producción de mediadores inmunes (Le G, J Med Virol 2020).

Probablemente muchos pacientes con COVID-19 grave tienen un proceso inflamatorio deletéreo que podría mejorar con la administración de terapias inmunosupresoras. Los corticoides no se recomiendan de forma generalizada y pueden agravar las lesiones pulmonares asociadas a COVID-19 (Russell CD, Lancet 2020), aunque podrían ser beneficiosos en los pacientes con mayor inflamación (Zhou F, Lancet. 2020). El bloqueo del receptor de IL-6 con Tocilizumab ha demostrado su eficacia para tratar pacientes con neumonía por COVID-19 y IL-6 elevada en un ensayo realizado en China (ChiCTR2000029765). De hecho, tocilizumab está siendo utilizado ya en múltiples protocolos asistenciales y probablemente durante las próximas semanas se publicarán nuevos resultados. EUSA Pharma ha iniciado un estudio con siltuximab para tratar pacientes con SDRA, que actúa también específicamente sobre IL-6 (SISCO Study). El principal inconveniente de estos fármacos es que bloquean sólo una citocina de la respuesta inmune y podría no ser suficiente para inhibir la gran respuesta inflamatoria que presentan estos pacientes. Otro inconveniente importante de tocilizumab es su escasez actual en los centros hospitalarios y su elevado coste. Existen una decena de estudios enfocados en el síndrome de dificultad respiratoria aguda, con aproximaciones que tienen un time to market muy elevado, dada su alta complejidad regulatoria, ética y experimental como el caso del ensayo de fase II ChiCTR2000030116, GDCT0380217 que basa su premisa en la utilización de células mesenquimales.

Nuestra hipótesis de trabajo es que la neumonía grave por SARS-CoV-2 (COVID-19) es secundaria a un proceso inflamatorio deletéreo que podría mejorar tras la administración de pulsos de metilprednisolona y tacrolimus.

Tacrolimus (también conocido como FK506) se está usando para evitar el rechazo en pacientes trasplantados y también para tratar múltiples enfermedades autoinmunes sistémicas (Azizi JR, J inmunológicas 2013). Los efectos de tacrolimus se deben a su unión a una proteína citosólica (FKBP12). El complejo FKBP12-tacrolimus se une de forma específica y competitiva a la calcineurina, y la inhibe. Tacrolimus inhibe la formación de linfocitos citotóxicos, inhibe la activación de los linfocitos T y la proliferación de los linfocitos B dependientes de los linfocitos T cooperadores. Tacrolimus inhibe vías de transducción de señales en los linfocitos T, lo que impide la transcripción de múltiples genes relacionados con citocinas proinflamatorias, así como interferones de tipo 1 (Hirano K, Liver Transplant 2008).

**Nombre del promotor:**<br>Dr. Xavier SOLANICH<br>**Nombre del Producto Terminado:**<br>- Urbason®, Solu-Moderín®<br>- Advagraf®, Modigraf®<br>**Nombre del Principio Activo:**<br>Metilprednisolona / Tacrolimus	**Para Uso Exclusivo de las Autoridades Sanitarias**

Nos basamos también en que la enfermedad por COVID-19 grave presenta un perfil clínico y de citocinas muy parecido a enfermedades como la limfohistiocitosi hemofagocítica secundaria (HLHs) (Mehta P. Lancet. 2020) o la clinically amyopathic dermatomyositis (CADM) asociada a anti-melanoma differentiation-associated gene 5 (MDA-5).

La HLHs es un síndrome hiperinflamatorio que se caracterizada por una tormenta de citocinas fulminante con fracaso multiorgánico. En adultos, el HLHs puede estar desencadenado por infecciones víricas. Las principales características del HLHs incluyen fiebre, hiperferritinemia, citopenias, etc. La afectación pulmonar (incluida el síndrome de distrés respiratorio agudo) se produce en hasta el 50% de los pacientes. La enfermedad por COVID-19 grave presenta un perfil de citocinas muy parecido al HLHs, caracterizado por un aumento de la IL-2, IL-7, granulocytecolony stimulating factor, interferon-γ inducible protein 10, monocyte chemoattractant protein 1, macrophage inflammatory protein 1-α, y tumour necrosis factor-α (Mehta P. Lancet. 2020). Los corticoides y los inhibidores de calcineurina, como tacrolimus, son el principal tratamiento de los HLHs (Ramos-Casals M, Lancet 2014).

La afectación pulmonar por COVID-19 tiene similitudes importantes también con la CADM asociada anti-MDA5. En esta enfermedad se producen, por motivos desconocidos, anticuerpos frente al receptor de la inmunidad innata MDA-5 que se encarga de la detección de virus. Hay una hiperactivación de este receptor y como consecuencia una gran cantidad de interferones de tipo 1 y citocinas proinflamatorias. En esta enfermedad es característica una enfermedad pulmonar intersticial rápidamente progresiva similar a la que sufren los pacientes con COVID-19. No hay un tratamiento estándar para esta enfermedad pero los agentes anticalcineurínicos, como tacrolimus, tienen un papel central en el tratamiento de esta entidad (Nara M, Mod Rheumatol 2014).

En este ensayo se cuantificarán múltiples citocinas y su variación durante la evolución de la enfermedad. Por lo tanto, del presente estudio se podrían generar nuevas hipótesis para bloquear distintas dianas terapéuticas en pacientes con COVID-19. No se ha descrito el papel que puede tener la IL-18, siendo esta una citocina muy relevante en los HLHs, a pesar de disponer de un fármaco que bloquea esta citocina (Tadikinib). Este fármaco se ha utilizado en pacientes con mutaciones en NLRC4-MAS en diferentes ensayos. También la citocina CXCL9 es un buen biomarcador de HLHs.

Llevaremos a cabo una prueba de concepto mediante un ensayo clínico, randomizado, controlado, abierto y unicéntrico para evaluar si pulsos de metilprednisolona y tacrolimus pueden ser útiles para tratar el distrés respiratorio agudo grave desencadenado por COVID-19, ofreciendo así una nueva opción terapéutica.

Si este primer ensayo mostrara resultados favorables de eficacia y seguridad, se corroborarían mediante un nuevo ensayo clínico, asignación aleatoria, controlado, doble ciego y multicéntrico con un número adecuado de pacientes. Estamos solicitando fondos públicos a través de convocatorias competitivas y, además hemos identificado dos posibles farmacéuticas que podrían estar interesadas en colaborar en este reprofilling (Astellas pharma y Teva pharmaceuticals) al comercializar los fármacos del estudio.

Dada la situación de emergencia sanitaria que representa el COVID-19 en este momento, este primer estudio podría darnos evidencia suficiente para empezar a tratar a los pacientes con mayor insuficiencia respiratoria e inflamación. Además, los corticoides y el tacrólimus son fármacos con un coste bajo y que se pueden fabricar a gran escala, por lo que se podríamos tratar a un gran número de pacientes.

OBJETIVOS:

Se define estabilidad clínica si se cumplen los siguientes criterios durante 48 horas consecutivas:

- Afebril: temperatura corpórea ≤ 37,5ºC sin uso de antitérmicos durante 48 horas
- PaO_2/FiO_2 >400 y/o $SatO_2/FiO_2$ >300
- Frecuencia respiratoria ≤ 24 rpm

Nombre del promotor:
Dr. Xavier SOLANICH
Nombre del Producto Terminado:
- Urbason®, Solu-Moderín®
- Advagraf®, Modigraf®

Nombre del Principio Activo:
Metilprednisolona / Tacrolimus

Para Uso Exclusivo de las Autoridades Sanitarias

Objetivo principal:
- Estudiar el tiempo (días) hasta alcanzar la estabilidad clínica tras iniciar la aleatorización en pacientes hospitalizados con neumonía grave secundaria a COVID-19, y elevación de parámetros inflamatorios.

Objetivos secundarios:

Objetivos Clínicos:
- Estudiar el tiempo hasta alcanzar un estado afebril durante 48 horas.
- Estudiar el tiempo hasta alcanzar PaO2/FiO2 >400 y/o SatO2/FiO2 >300
- Estudiar el tiempo hasta alcanzar una FR ≤ 24 rpm durante 48 horas.
- Estudiar el tiempo hasta la normalización de dímero D (<250 ug/L)
- Estudiar el tiempo hasta la normalización de la PCR (<5mg/L).
- Estudiar el tiempo hasta la normalización de la ferritina (<400ug/L).
- Cuantificar el estado clínico diariamente según la escala ordinal de la OMS.
- Cuantificar la duración (días) del tratamiento con tacrolimus.
- Cuantificar la duración (días) de la estancia hospitalaria.
- Porcentaje de pacientes que requieren dispositivos de soporte ventilatorio (VMNI y/o VM y/o GNAF)
- Cuantificar la duración (días) que es necesario mantener soporte ventilatorio.
- Describir las secuelas radiológicas y funcionales pulmonares atribuidas a COVID-19 a los 56 días del inicio del ensayo.
- Incidencia de mortalidad por COVID-19 a los 28 y 56 días del inicio del tratamiento del ensayo.
- Incidencia de mortalidad por cualquier causa a los 28 y 56 días del inicio del tratamiento del ensayo.
- Incidencia de recaídas de neumonía por COVID-19 a los 28 y 56 días del inicio del tratamiento del ensayo.
- Estudiar el impacto del tratamiento inmunosupresor en la dinámica viral mediante PCR cuantitativa.
- Analizar el perfil de citocinas ampliado antes del inicio del tratamiento y cada 7 días durante el ingreso.

Objetivos de Seguridad:
- Describir la incidencia de acontecimientos adversos según su gravedad y relación con el tratamiento del ensayo.

DISEÑO DEL ESTUDIO: Ensayo clínico de Fase II, pragmático, con asignación aleatoria, controlado, abierto y unicéntrico para evaluar la eficacia de los pulsos de metilprednisolona y tacrolimus en pacientes hospitalizados con neumonía grave secundaria a COVID-19, y parámetros inflamatorios elevados

Ámbito del estudio:

Este estudio se llevará a cabo en el Hospital Universitari de Bellvitge (HUB) que está ubicado en el municipio de L'Hospitalet de Llobregat y es uno de los 5 hospitales de tercer nivel acreditados de Cataluña lo que le permite atender a pacientes de alta complejidad. El HUB actúa como hospital de primer, segundo y tercer nivel. Como primer nivel, el HUB es el hospital general básico para 343.172 habitantes. Además como segundo nivel es referencia para especialidades de complejidad media y alta de una población aproximada de 1,3 millones de habitantes. Finalmente, en las especialidades de alta complejidad (tercer nivel), es hospital de referencia para todo el Eje Sur de Cataluña, aproximadamente 2 millones de habitantes (regiones sanitarias de Metropolitana Sur, Camp de Tarragona y Terres de l'Ebre). A fecha de 23 de Marzo de 2020 tenemos ingresados más de 300 pacientes con insuficiencia respiratoria y/o neumonía secundaria a COVID-19, y la cantidad de ingresos aumenta de forma rápida como en el resto de centros sanitarios españoles. Así pues, los pacientes atendidos en nuestro centro son suficientes para realizar el presente estudio.

En el ámbito de la investigación del HUB forma parte del Institut d'Investigació Biomèdica de Bellvitge (IDIBELL). Se encuentra en el corazón del Biopol de Hospitalet, un clúster que aprovecha la concentración en un solo espacio de hospitales, universidad, centros de investigación y empresas para promover la transferencia de conocimiento y la generación de valor añadido en el ámbito de las ciencias de la salud.

Grupos

**Nombre del promotor:**<br>Dr. Xavier SOLANICH<br>**Nombre del Producto Terminado:**<br>- Urbason®, Solu-Moderín®<br>- Advagraf®, Modigraf®<br>**Nombre del Principio Activo:**<br>Metilprednisolona / Tacrolimus	**Para Uso Exclusivo de las Autoridades Sanitarias**

Los pacientes incluidos en el estudio serán asignados de manera aleatoria (1:1) al

- **Grupo experimental:** pulsos de metilprednisolona 120mg/día durante 3 días consecutivos junto con tacrolimus* a la dosis necesaria para conseguir unos niveles plasmáticos de entre 8-10 ng/ml. Se suspenderá el tratamiento en el momento que se consiga la estabilidad clínica. Además estos pacientes podrán recibir todos los tratamientos que se consideren necesarios para su manejo clínico.

  ^ No se administraran los bolus si los 7 días previos a la aleatorización el paciente ha recibido ≥ 3 bolus de metilpredisolona (o dexametasona ≥ 20mg/día durante ≥ 3 dias). Si ha recibido menos de 3 bolus se completaran hasta un número total de 3.

  * Tacrolimus (Modigraf o Advagraf): Dosis inicial recomendada 0.05mg/kg cada 12 horas y ajustar según niveles. Si toma hidroxicloroquina 0.1mg/kg i ajustar según niveles igual. Si toma Lopinavir dosis inicial de 0.2mg cada 48 horas. En caso de Insuficiencia renal y/o insuficiencia hepática administrar las dosis descritas anteriormente sin modificaciones. Si requiere tratamiento endovenoso o tiene dudas del tratamiento contactar con farmacia (Dra Núria Padulles).

- **Grupo control:** el régimen de tratamiento estándar consistirá en administrar el tratamiento antiviral elegido por el médico responsable del paciente, además de todos los tratamientos que se consideren necesarios para su manejo clínico.

Visitas:

- **Visita basal (día 0, inclusión del paciente, inicio del tratamiento):** en esta visita se realizará una evaluación clínica y se confirmará que el paciente cumple todos los criterios de inclusión y ninguno de exclusión. Se informará al paciente sobre el estudio y se le entregará la hoja de información y el consentimiento informado para su firma. Se procederá a la asignación del tratamiento y se iniciará el mismo. Se registrarán datos demográficos, clínicos y analíticos, así como la medicación concomitante.

- **Durante el ingreso hospitalario (número de días variable según el período de ingreso):** se recogerá diariamente de la historia clínica todas las variables relacionadas con los criterios de estabilidad clínica (temperatura, satO2, FiO2, FR y tensión arterial). Se realizará un ECG i una analítica cada 48 horas con los parámetros que se especifican en el apartado de laboratorio.

- **Visita al día 28 ±3 del inicio del tratamiento:** Se evaluará si los pacientes han realizado el tratamiento según el protocolo, si cumplen los criterios de estabilidad clínica, si han presentado una recaída o empeoramiento de la enfermedad, así como la presencia de acontecimientos adversos. Esta visita será presencial..

- **Visita al día 56 ±3 del inicio del tratamiento (fin de estudio):** Se evaluará si los pacientes han realizado el tratamiento según el protocolo, si cumplen los criterios de estabilidad clínica, si han presentado una recaída o empeoramiento de la enfermedad, así como la presencia de acontecimientos adversos. Esta visita será presencial..

Base de datos y monitorización

Al ser un estudio exploratorio se evaluarán 42 pacientes en el grupo experimental y 42 pacientes tratados según el tratamiento estándar. Para realizar el ensayo utilizaríamos la plataforma informática RedCap que permite randomizar y recoger los datos de los pacientes. Esta base de datos se realizará con la colaboración del equipo del Dr. Cristian Tebe, jefe de la **Unidad de Estadística del IDIBELL.** El estadístico que realizará los análisis será ciego para el tratamiento que reciban los pacientes (intervención vs tratamiento convencional). Esta unidad se encargará de la gestión y análisis de los datos de forma dinámica durante el estudio por si se detectaran diferencia importantes entre los grupos de forma precoz. Además, la **Unidad de Investigación Clínica en Ensayos Clínicos del IDIBELL** se encargará de la monitorización y fármacovigilancia del ensayo.

Datos demográficos y clínicos:

En la visita basal se recogerán las siguientes variables: filiación (código anonimizado, edad, sexo), enfermedades o condiciones debilitantes presentes en el índice de Charlson (https://www.mdcalc.com/charlson-comorbidity-index-cci),

**Nombre del promotor:**<br>Dr. Xavier SOLANICH<br>**Nombre del Producto Terminado:**<br>- Urbason®, Solu-Moderín®<br>- Advagraf®, Modigraf®<br>**Nombre del Principio Activo:**<br>Metilprednisolona / Tacrolimus	**Para Uso Exclusivo de las Autoridades Sanitarias**

constantes vitales (temperatura, satO2 basal, frecuencia respiratoria y tensión arterial), índices para evaluar la gravedad de la neumonía [PSI (http://www.samiuc.es/pneumonia-severity-index-psi/) y CURB-65 (http://www.semergencantabria.org/calc/cpcalc2.htm)], tratamientos concomitantes.

En las visitas sucesivas se recogerán constantes vitales (temperatura, satO2, FiO2, frecuencia respiratoria y tensión arterial), efectos secundarios atribuidos a la medicación del ensayo o a otros tratamientos, fecha del alta hospitalaria, mortalidad atribuida a la medicación del ensayo o a otros tratamientos.

Exploraciones complementarias:

ECG y analíticas de rutinaria cada 48 horas:

El HUB dispone de las instalaciones y aparataje necesario para procesar i analizar las muestras de los pacientes con COVID-19. Se ha creado un preconfigurado en el SAP del HUB con todas las variables del estudio. Se realizará una analítica cada 48 horas con hemograma [recuento de glóbulos blancos (WBC), recuento de linfocitos (LYM), recuento de neutrófilos (NEU)] mediante analizador Sysmex XN2000, bioquímica [Aspartate aminotransferasa (AST), Alanina aminotransferasa (ALT), bilirrubina (BIL), gamma-glutamil transpeptidasa (GGT), fosfatasa alcalina (FA), Lactato Deshidrogenasa (LDH), Ferritina, Glucosa (Glu), Urea, Creatinina (Cr), Calcio (Ca), albúmina (ALB), creatinina cinasa (CK), C-reactive protein (CRP), NT–proBNP, troponinas (TN), procalcitonina (PCT) y IL-6] que se medirán con un analizador se medirán con un analizador Cobas 6000/8000 (Roche Diagnostics) que posee módulos de espectrofotometría y de inmunoquímica con detección electroquimioluminiscente. La coagulación [el D-Dímero (DD), tiempo de trombina (TT), tiempo de protrombina (PT), Fibrinógeno (FIB), tiempo de tromboplastina parcial activada (APTT)] se determinará mediante el analizador de ACLTOP 550 (Werfen). Los niveles de tacrolimus se medirán mediante cromatografía líquida de alta y rápida eficacia (UHPLC) acoplada a la espectrometría de masas en tándem (MS/MS)

Estudio de perfil ampliado de citosina:

Se dispone de **Biobanco** del HUB que cumple con todos los requisitos de la legislación vigente. Se encargará del procesamiento y almacenamiento (suero congelado) de muestras de pacientes con COVID-19. Se analizaran las muestras de los 84 pacientes antes de iniciar el tratamiento del ensayo y 7 días después del inicio de su administración. Tras obtener todas las muestras, se enviaran al Centro de Diagnóstico Biomédico del Laboratorio de Immunologia del Hospital Clínic de Barcelona para realizar una técnica de **cuantificación multiple de citocinas por Luminex** en los sueros de los pacientes infectados con COVID19. Analizaremos la concentración de las siguientes citocinas: IL-1alpha, IL-1beta, IL-1RA, IL-2, IL-4, IL-6, IL-8, IL-7, IL-10, IL-18, TNF -alpha, IFN-alpha, IFN-beta, IFN-gamma, CXCL10 / IP10, CXCL9 / MIG, MCP-1 / CCL2, MIP-1alpha, G-CSF e IL-2R / CD25. Además se determinaran parámetros virológicos (PCR cuantitativa) para evaluar el impacto del tratamiento inmunosupresor en la dinámica viral.

Farmacia

El **servicio de farmacia** de Bellvitge se encargaría de que la dosis de tacrolimus sea la correcta según las especificaciones del protocolo. . El grupo de tratamiento recibirá la tacrolimus según la dosis descrita en apartado "grupos". Se administrará 1 hora antes de comer para obtener la máxima absorción. Las concentraciones en sangre se determinarán con métodos de inmunoensayo. Las concentraciones en sangre de tacrolimus se analizarán 24 horas después de la última dosis de tacrolimus, justo antes de la siguiente administración. Esta determinación se realizará cada 2 días mientras el paciente reciba el fármaco para mantener unos niveles plasmáticos entre 8 y 10 ng/ml. El servicio de farmacia del HUB se encargará de ajustar la dosis del fármaco.

El tacrolimus se metaboliza a través del CYP3A4 hepático y de la pared intestinal. El uso concomitante de sustancias conocidas por inhibir o inducir CYP3A4 puede afectar el metabolismo de tacrolimus y, por tanto, elevar o disminuir su concentración sanguínea. Fármacos que se están utilizando para tratar la infección por COVID-19, como por ejemplo los inhibidores de la proteasa para el VIH (ritonavir) inhiben el CYP3A4. Por lo tanto, es de esperar un aumento de las concentraciones de tacrolimus en sangre. Para evitar complicaciones, se dará una dosis inicial baja (0.2mg cada 48 horas) que se ajustará según los niveles plasmáticos. Habrá que revisar también otros fármacos que puedan influir en el metabolismo de tacrólimus. Una de las complicaciones que más preocupa a los investigadores es la prolongación del intervalo QT (trastorno también descrito con lopinavir / ritonavir). Para evitar arritmias derivadas de este trastorno, se evaluará el intervalo QT durante el estudio mediante un ECG cada 48 horas y se retirará el fármaco del estudio si

**Nombre del promotor:**<br>Dr. Xavier SOLANICH<br>**Nombre del Producto Terminado:**<br>- Urbason®, Solu-Moderín®<br>- Advagraf®, Modigraf®<br>**Nombre del Principio Activo:**<br>Metilprednisolona / Tacrolimus	**Para Uso Exclusivo de las Autoridades Sanitarias**

es superior a 550mseg. Mediante los controles de constantes vitales diarios y las analíticas rutinarias se evaluarán otros potenciales efectos adversos del fármaco. Se ha descrito un mayor riesgo de infecciones, incluidas infecciones oportunistas, pero los investigadores no esperamos que los pacientes desarrollen este tipo de complicaciones ya que el fármaco se administrará durante un corto período de tiempo.

POBLACIÓN DEL ESTUDIO:

Los sujetos del estudio serán reclutados de forma prospectiva e incluidos en el estudio para su posterior asignación aleatoria, si cumplen los criterios de inclusión y no existe motivo de exclusión, tal y como se indican a continuación:

Criterios de inclusión:

Pacientes ingresados en el HUB con infección por COVID-19 confirmada mediante fluorescent RT-PCR i que cumplan todos los criterios siguientes:

1- Infiltrados radiológicos de nueva aparición (bien por radiografía simple de tórax, tomografía axial computarizada o ecografía de tórax) atribuidos a COVID-19,

2- Insuficiencia respiratoria ($PaO_2/FiO_2 < 300$ o $satO_2/FiO_2 < 220$) atribuida a 1,

3- PCR > 100 mg/L y/o D-Dimero > 1000 µg/L y/o Ferritina > 1000 ug/L atribuida a 1,

4- Edad ≥ 18 años.

5- El sujeto, su representante legal o familiar más cercano (en caso de incapacidad del sujeto por gravedad de la situación clínica) otorgan el consentimiento informado

Criterios de exclusión:

- Muerte inminente (expectativa de vida ≤ a 24h).
- Filtrado glomerular ≤ 30 ml/min /1,73 m2 (estimado según la ecuación CKD-EPI)
- Leucopenia ≤ 4000 células/µL
- Infecciones concomitante potencialmente graves.
- Contraindicación para el uso de tacrolimus según la ficha técnica del producto.
- Reacciones adversas conocidas al tratamiento
- Haber participado en un ensayo clínico los últimos 3 meses.

TRATAMIENTO EXPERIMENTAL: Terapia inmunosupresora:

De forma empírica se está utilizando:

- Metilprednisolona (Urbason®, Solu-Moderín®)
- Tacrolimus (Advagraf®, Modigraf®)

CRITERIOS DE VALORACIÓN:

Criterio principal (para el objetivo principal de eficacia):

- Tiempo (días) hasta alcanzar la estabilidad clínica después de iniciar el tratamiento del ensayo, en pacientes hospitalizados con neumonía grave secundaria a COVID-19 y parámetros inflamatorios elevados.

Se definirá **éxito del tratamiento** si los pacientes cumplen los criterios de estabilidad clínica durante 48 horas.

Se define **fracaso del tratamiento** si

- paciente que no cumple los criterios de estabilidad clínica a los 56 días de haber iniciado el tratamiento.
- paciente que presenta una reacción adversa de grado 3 o 4 atribuida al tratamiento del ensayo, o

**Nombre del promotor:**<br>Dr. Xavier SOLANICH<br>**Nombre del Producto Terminado:**<br>- Urbason®, Solu-Moderín®<br>- Advagraf®, Modigraf®<br>**Nombre del Principio Activo:**<br>Metilprednisolona / Tacrolimus	**Para Uso Exclusivo de las Autoridades Sanitarias**

- paciente que fallece tras haber sido incluido en el ensayo clínico

Criterios secundarios (para los objetivos secundarios):

Se define estado clínico del paciente de acuerdo con la siguiente escala ordinal de la OMS (reference https://clinicaltrials.gov/ct2/show/NCT04280705):

7) muerte
6) Hospitalizado, con ventilación mecánica invasiva o ECMO
5) Hospitalizado, con ventilación no invasiva o dispositivos de oxígeno de alto flujo
4) Hospitalizado, que requiere oxígeno suplementario de bajo flujo
3) Hospitalizado, que no requiere oxígeno suplementario - requiere atención médica continua (COVID-19 relacionado o no)
2) Hospitalizado, que no requiere oxígeno suplementario, ya no requiere atención médica continua
1) No hospitalizado

Clínicos y analíticos:

- Tiempo (‘días’) hasta alcanzar un estado afebril durante 48 horas.
- Tiempo (‘días’) hasta alcanzar una PaO2/FiO2 >400 y/o SatO2/FiO2 >300 durante 48 horas.
- Tiempo (‘días’) hasta alcanzar una FR ≤ 24 rpm durante 48 horas
- Tiempo (‘días’) hasta la normalización del dímero D (<250 ug/L),
- Tiempo (‘días’) hasta la normalización de IL-6 (<5mg/L).
- Tiempo (‘días’) hasta la normalización de Ferritina (<400ug/L)
- Cambio (porcentaje) de la cantidad viral (PCR) antes del inicio del fármaco respecto al día 7.
- Número de pacientes que requiere dispositivos de soporte ventilatorio no invasivo (VMNI, GNAF…)
- Número de pacientes que requiere dispositivos de soporte ventilatorio invasivo (VM)
- Tiempo (‘días’) en unidad de cuidados intensivos.
- Tiempo (‘días’) en unidad de cuidados semi-intensivos.
- Número de días de estancia hospitalaria (desde el día del inicio del tratamiento del ensayo hasta el alta hospitalaria)
- Descripción del estado clínico según la OMS durante el periodo hasta el alta hospitalaria
- Número de pacientes que alcanzan un estado clínico ≤ 2 tras 10 días del inicio del tratamiento o al alta, lo que suceda antes.
- Número de pacientes que alcanzan la estabilidad clínica a los 10 días del inicio del tratamiento o al alta, lo que suceda antes.
- Valor medio de cada uno de los valores analíticos (dímero D, IL-6, ferritina) a los 10 días o al alta, lo que suceda antes.
- Número de días con el tratamiento del ensayo.
- Cambio de la carga viral cuantitativa mediante RT-PCR entre las muestras antes del inicio del fármaco y las realizadas semanalmente durante la hospitalización.
- Cuantificar las citocinas estudiadas en el perfil de citocinas ampliado antes del inicio del tratamiento, y semanalmente durante la hospitalización.
- Eficacia a largo plazo (a los 28 y 56 días del inicio del tratamiento del ensayo) se evaluara midiendo si se mantiene la estabilidad clínica y la incidencia de recaídas de neumonía por COVID-19.
- Describir las alteraciones radiológicas (radiografía simple, TAC tórax pruebas funcionales) a los 56±3 días del inicio del tratamiento del ensayo,

Nombre del promotor:
Dr. Xavier SOLANICH
Nombre del Producto Terminado:
- Urbason®, Solu-Moderín®
- Advagraf®, Modigraf®

Nombre del Principio Activo:
Metilprednisolona / Tacrolimus

Para Uso Exclusivo de las Autoridades Sanitarias

Seguridad:

- Incidencia de acontecimientos adversos según su gravedad y relación con el tratamiento del ensayo.

Mortalidad:

- Incidencia de mortalidad por COVID-19 a los 28 y 56 días del inicio del tratamiento del ensayo.
- Incidencia de mortalidad por cualquier causa a los 28 y 56 días del inicio del tratamiento del ensayo.

DURACIÓN DEL ENSAYO CLÍNICO:

Se prevé comenzar el ensayo clínico en marzo / abril de 2020. Estamos realizando ya la base de datos informatizada que podría estar a punto antes de finalizar marzo.

Teniendo en cuenta la gran cantidad de pacientes con COVID-19 que cumplen los criterios del estudio que estamos ingresando en nuestro centro, en menos de 2 semanas deberíamos haber reclutado y randomizado a los 84 pacientes. A partir de las 4 semanas de haber iniciado el reclutamiento se podría disponer de los primeros resultados y ser publicados rápidamente. Los resultados del seguimiento a largo plazo (30 días tras el alta hospitalaria) podrían estar publicados a partir de los 3 meses del inicio de la randomización.

Si este primer ensayo mostrara resultados favorables de eficacia y seguridad, se corroborarían mediante un nuevo ensayo clínico, asignación aleatoria, controlado, doble ciego y multicéntrico con un número mayor de pacientes. Estamos solicitando fondos públicos a través de convocatorias competitivas y, además hemos identificado dos posibles farmacéuticas que podrían estar interesadas en colaborar en este reprofilling (Astellas pharma y Teva pharmaceuticals) al comercializar los fármacos del estudio.

DETERMINACIÓN DEL TAMAÑO DE LA MUESTRA:

El tiempo mediano hasta la estabilidad clínica en grupo control se espera que sea de 16 días. Si el hazard ratio de estabilidad clínica de los pacientes de control en relación a los pacientes del grupo experimental es 0,52, habrá que incluir 42 pacientes en cada grupo para poder rechazar la hipótesis nula de igualdad con una potencia del 80%. La probabilidad de error Tipo I asociada con esta prueba de hipótesis es 0,05, y en cálculo incluye un 5% de pérdidas.

CONSIDERACIONES ESTADÍSTICAS:

Todos los datos recogidos en el estudio se resumirán empleando los métodos estadísticos apropiados. Dichos resúmenes se estratificarán por los grupos a estudio.

Se realizará un análisis descriptivo de las variables del estudio. Las variables continuas serán descritas como media y Desviación Estándar (DE) o como mediana y rango; y las variables categóricas como frecuencias absolutas y porcentajes.

El análisis principal se realizará cuando todos los pacientes hayan alcanzado la estabilidad clínica, fracaso clínico o bien, lleven 56 días ingresados ante lo cual serán considerados fracasos también. La comparación entre el tiempo hasta la estabilidad clínica entre los grupos de estudio se realizará mediante el test de log-rank. Para cuantificar la magnitud de la asociación se estimará el hazard ratio con un modelo de riesgos proporcionales de Cox y se acompañará de los intervalos de confianza al 95%. La variable principal se analizará además en la población por protocolo.

A la mitad del reclutamiento, 21 pacientes por brazo, se realizará un análisis intermedio de eficacia y seguridad. Para ello se aplicará una corrección del error de tipo I siguiendo Lan–DeMets (O'Brien–Fleming) en la evaluación de la eficacia.

Como análisis adicional, se construirá un modelo de riesgos proporcionales de Cox ajustado por factores de confusión

**Nombre del promotor:**<br>Dr. Xavier SOLANICH<br>**Nombre del Producto Terminado:**<br>- Urbason®, Solu-Moderín®<br>- Advagraf®, Modigraf®<br>**Nombre del Principio Activo:**<br>Metilprednisolona / Tacrolimus	**Para Uso Exclusivo de las Autoridades Sanitarias**

clinicalmente relevantes como la edad, sexo, comorbilidades mediante el índice de Charlson, índices para valorar la gravedad de la neumonía (PSI y CURB-65), índices de disfunción orgánica (SOFA) y parámetros inflamatorios. Además, está planificado un análisis de subgrupos, para investigar si el efecto del tratamiento varía en función de la gravedad de COVID-19, en los pacientes con un mayor grado de comorbilidades y por grupos etarios.

Para la evaluación de las variables secundarias calcularemos las estimaciones no ajustadas y ajustadas del tamaño del efecto y los correspondientes intervalos de confianza del 95% utilizando regresión lineal, logística o de riesgos proporcionales de Cox.

Los acontecimientos adversos del estudio serán descritos de acuerdo a la gravedad y la relación con el tratamiento del ensayo, y se compararán entre grupos de tratamiento. Por motivos de seguridad se realizará una monitorización al día de la mortalidad.

El estadístico que finalmente realizará los análisis será ciego para el tratamiento recibido por los pacientes (régimen corto o largo de tratamiento antiviral). Se utilizará R versión 3.6.2 o superior para Windows (R Foundation for Statistical Computing, http://www.r-project.org) para el tratamiento y análisis de datos.

CONSIDERACIONES ÉTICAS:

El estudio está siendo evaluado de urgencia por el Comité de Ética de la Investigación con medicamentos del HUB y por la Agencia Española de Medicamentos y Productos Sanitarios, de acuerdo a la legislación vigente, el Real Decreto 1090/2015 de 4 de diciembre y el Reglamento Europeo 536/2014 de 16 de abril, por los que se regulan los ensayos clínicos con medicamentos.

Tanto el Promotor como el centro son responsables respectivamente del tratamiento de los datos del paciente y se comprometen al cumplimiento del Reglamento (UE) 2016/679 del Parlamento europeo y del Consejo de 27 de abril de 2016 de Protección de Datos (RGPD), así como al resto de leyes y normativa vigente y aplicable (Ley Orgánica de Protección de Datos Personales y Garantía de los Derechos Digitales 3/2018 del 05 de Diciembre). Los datos recogidos para el estudio estarán identificados mediante un código, de manera que no incluya información que pueda identificarle (ni nombre ni apellidos, ni iniciales ni dirección, nº seguridad social, etc), y sólo su médico del estudio/colaboradores podrá relacionar dichos datos con el paciente y con su historia clínica. Por lo tanto, la identidad no será revelada a ninguna otra persona salvo a las autoridades sanitarias, cuando así lo requieran o en casos de urgencia médica. Si el participante quiere saber más al respecto, puede contactar al Delegado de Protección de Datos del Promotor (dataprotection@idibell.cat).

La **Unidad de Estadística del IDIBELL** se encargará de la gestión y análisis de los datos de forma dinámica durante el estudio por si se detectaran diferencia importantes entre los grupos de forma precoz. Además, la **Unidad de Investigación Clínica en Ensayos Clínicos del IDIBELL** se encargará de la monitorización y fármacovigilancia del ensayo.

2. ÍNDICE

1. RESUMEN 3
2. ÍNDICE 12
3. LISTADO DE ABREVIATURAS 15
4. PROCEDIMIENTOS ÉTICOS Y REGLAMENTARIOS DEL ESTUDIO 16
4.1 CONSIDERACIONES ÉTICAS 16
4.2 HOJA DE INFORMACIÓN AL PACIENTE Y CONSENTIMIENTO INFORMADO 16
4.3 PROTECCIÓN DE DATOS DE LOS PACIENTES 17
4.4 COMITÉ DE ÉTICA DE LA INVESTIGACIÓN CON MEDICAMENTOS 18
4.5 SEGURO ESPECÍFICO DE ENSAYOS CLÍNICOS 18
4.6 SISTEMA DE CALIDAD, AUDITORÍA E INSPECCIÓN 18
4.6.1 SISTEMA DE CALIDAD 18
4.6.2 AUDITORÍA 19
4.6.3 INSPECCIÓN 19
4.7 MODIFICACIONES AL PROTOCOLO 19
4.8 CONTRATOS 19
5. INVESTIGADORES Y ESTRUCTURA ADMINISTRATIVA DEL ESTUDIO 20
6. INTRODUCCIÓN 22
6.1 COVID-19 22
6.2 MEDICAMENTO EN INVESTIGACIÓN 25
7. OBJETIVOS DEL ESTUDIO 26
7.1 OBJETIVO PRINCIPAL 26
7.2 OBJETIVOS SECUNDARIOS 26
7.3 OBJETIVOS DE SEGURIDAD 26
8. PLAN DE INVESTIGACIÓN 27
8.1 DISEÑO DEL ENSAYO CLÍNICO 27
8.2 POBLACIÓN DEL ESTUDIO Y PROCEDIMIENTO DE RECLUTAMIENTO 27
8.2.1 CRITERIOS DE SELECCIÓN 27
8.2.1.1 Criterios de inclusión 27
8.2.1.2 Criterios de exclusión 28
8.3 ASIGNACIÓN ALEATORIA Y ENMASCARAMIENTO 28
8.4 CRITERIOS DE VALORACIÓN 28
8.4.1 CRITERIOS DE VALORACIÓN DE LA EFICACIA 28
8.4.1.1 Criterio principal (para el objetivo principal) 28
8.4.1.2 Criterios secundarios (para los objetivos secundarios) 29
8.4.1.3 Criterios de valoración de la seguridad 30
8.5 PERIODOS Y DURACIÓN DEL ESTUDIO 30
8.6 RETIRADA DE SUJETOS 30
8.7 CUIDADO DE LOS PACIENTES TRAS FINALIZAR EL ESTUDIO 31
9. DESCRIPCIÓN DEL TRATAMIENTO 32
9.1 DOSIS, INTERVALO, VÍA, FORMA DE ADMINISTRACIÓN Y DURACIÓN DE LOS TRATAMIENTOS DE ENSAYO 32
9.2 PRECAUCIONES CON LOS MEDICAMENTOS DE ESTUDIO. 32

9.3 GESTIÓN DE LA MEDICACIÓN DEL ESTUDIO. 33

10. PARÁMETROS Y PRUEBAS DE EVALUACIÓN 34
10.1 CLÍNICOS 34
10.2 EXPLORACIONES COMPLEMENTARIAS 34

11. PROCEDIMIENTOS Y CALENDARIOS 36
11.1 NÚMERO E INTERVALO DE VISITAS DEL ESTUDIO 36

12. DOCUMENTACIÓN DE LOS DATOS DEL ESTUDIO 39
12.1. CUADERNO DE RECOGIDA DE DATOS 39
12.2. ARCHIVO DEL INVESTIGADOR 39
12.3. CONFIDENCIALIDAD DE LOS RESULTADOS DEL ENSAYO CLÍNICO 39
12.4. POLÍTICA DE PUBLICACIÓN 40
12.5. INFORME FINAL 40

13. SEGURIDAD DEL MEDICAMENTO DE ESTE ESTUDIO 41
13.1 DEFINICIONES 41
13.1.1. ACONTECIMIENTO ADVERSO / REACCIÓN ADVERSA 41
13.1.2. ACONTECIMIENTO ADVERSO GRAVE Y REACCIÓN ADVERSA GRAVE 41
13.1.3. REACCIÓN ADVERSA INESPERADA 41
13.1.4. REACCIÓN ADVERSA GRAVE E INESPERADA 41
13.1.5. CAUSALIDAD CON EL MEDICAMENTO EN INVESTIGACIÓN 41
13.1.6. INFORMACIÓN DE SEGURIDAD DE REFERENCIA 42
13.1.7. VALORACIÓN DE LOS ACONTECIMIENTOS ADVERSOS 42
13.2 SEGUIMIENTO DE LOS PACIENTES CON ACONTECIMIENTOS ADVERSOS 43
13.3. NOTIFICACIÓN DE LOS ACONTECIMIENTOS ADVERSOS 43
13.4. REACCIONES ADVERSAS GRAVES E INESPERADAS 44
13.5. ACONTECIMIENTOS ADVERSAS DE ESPECIAL INTERÉS 44
13.6. APERTURA DEL CIEGO Y DESENMASCARAMIENTO 44
13.7. NOTIFICACIÓN EXPEDITIVA DE OTRA INFORMACION DE SEGURIDAD RELEVANTE 44
13.8. EMBARAZO 44
13.9. INFORME A LOS INVESTIGADORES 45

14. CONSIDERACIONES ESTADÍSTICAS 46
14.1 DETERMINACIÓN DEL TAMAÑO DE LA MUESTRA 46
14.2. GRUPOS DE ANÁLISIS 46
14.2.1. GRUPO DE ANÁLISIS DE SEGURIDAD 46
14.2.2. GRUPO DE ANÁLISIS POR INTENCIÓN DE TRATAR (ITT) 46
14.2.3. GRUPO DE ANÁLISIS POR PROTOCOLO (PP) 46
14.3. MÉTODOS ESTADÍSTICOS 47
14.3.1. DATOS DEMOGRÁFICOS Y BASALES 47
14.3.2. RETIRADAS Y ABANDONOS 47
14.3.2.1. Análisis principal de eficacia 47
14.3.2.2. Análisis secundarios de eficacia 47
14.3.3. SEGURIDAD 48
14.3.4. ANÁLISIS INTERMEDIOS 48

15. REFERENCIAS BIBLIOGRÁFICAS 49

16. APÉNDICES 51
16.1 LISTADO DE INVESTIGADORES PRINCIPALES DE LOS CENTROS: 51
16.2 APÉNDICE 2: FICHA TÉCNICA 52

16.3 APÉNDICE 3: HOJA INFORMACIÓN PACIENTE 53

3. LISTADO DE ABREVIATURAS

**AA**	Acontecimiento adverso
**AAG**	Acontecimiento adverso grave
**AEMPS**	Agencia Española del Medicamento y Productos Sanitarios
**BPC**	Buena práctica clínica
**CEIm**	Comité de Ética de Investigación con medicamentos
**CRD-e**	Cuaderno de recogida de datos electrónico
**DE**	Desviación estándar
**EoT**	End of Treatment
**FT**	Ficha técnica del Producto en investigación
**ICH**	Conferencia Internacional de Armonización *(International Conference on Harmonisation*)
**ISR**	Información de seguridad de referencia
**ITT**	Intención de tratar *(Intention to treat analysis)*
**LLT**	Términos del nivel más bajo de MedDRA (*Lowest Level Term*)
**LOCF**	Técnica de arrastre de la última observación disponible (*Last Observation Carried Forward*)
**MedDRA**	*Medical Dictionary for Regulatory Activities*
**FT**	Ficha técnica del Producto en investigación
**PP**	Por protocolo
**PaO_2**	Presión arterial de Oxígeno
**PCR**	Proteína C reactiva
**PROs**	Resultados percibidos por los pacientes (*Patient-Reported Outcomes*)
**PSI**	Pneumonía severity index
**RA**	Reacción Adversa
**RAG**	Reacción Adversa Grave
**RAGIs**	Reacciones adversas graves e inesperadas (en inglés SUSARs: *suspected unexpected serious adverse reactions*)
**RAM**	Reacciones adversas a medicamentos
**SoC**	“Standard of Care”
**TA**	Tensión arterial
**ToC**	Test of Cure
**UICEC**	Unidad de Investigación Clínica y Ensayos Clínicos
**UCI**	Unidad Cuidados Intensivos

4. PROCEDIMIENTOS ÉTICOS Y REGLAMENTARIOS DEL ESTUDIO

4.1 CONSIDERACIONES ÉTICAS

Este estudio se realizará de acuerdo con los principios éticos basados en la última versión de la Declaración de Helsinki (acordada por la 64ª Asamblea General de la Asociación Médica Mundial, en Fortaleza, Brasil, en octubre de 2013), la Buena Práctica Clínica (BPC) y las normativas aplicables.

La BPC es un estándar para todos los aspectos que conciernen a los ensayos clínicos (diseño, realización, desarrollo, monitorización, auditoría, registro, análisis y comunicación) que permitan asegurar que los datos y resultados transmitidos son creíbles y exactos, y que quedan protegidos los derechos, integridad y confidencialidad de los pacientes.

El Investigador se responsabiliza de garantizar que el ensayo clínico se realice siguiendo el protocolo, las directrices establecidas por la Conferencia Internacional de Armonización (ICH: International Conference on Harmonisation) sobre la BPC y los requerimientos legales locales.

El estudio sólo podrá comenzar una vez que el CEIm y la AEMPS hayan dado su aprobación fechada y firmada del protocolo, así como de los modelos de Hoja de Información al Paciente y Consentimiento Informado.

Cada persona implicada en la realización del estudio debe estar cualificada por su educación, formación y experiencia para realizar sus tareas específicas.

Ni los investigadores ni el personal del estudio coaccionarán o ejercerán presión alguna sobre los pacientes para que participen o continúen en el estudio. El Investigador no podrá incluirse a sí mismo en el estudio, o a sus familiares ni a miembros de su equipo clínico o a los familiares de éstos.

4.2 HOJA DE INFORMACIÓN AL PACIENTE Y CONSENTIMIENTO INFORMADO

Antes de realizar cualquier procedimiento relacionado con el estudio, El investigador se responsabilizará de entregar a cada paciente (o representante legal, si procede) la *Hoja de Información al Paciente* específicamente preparada y aprobada para el estudio, la cual deberá ser leída por el paciente.

Se utilizará un lenguaje e idioma que el paciente pueda comprender, para informarle sobre el Medicamento en Investigación, los objetivos del estudio, y las ventajas y desventajas de su participación en el estudio.

Se dejará a los pacientes el tiempo suficiente para que puedan considerar su participación y se contestarán todas las preguntas que formulen.

El investigador informará a los pacientes de que su participación en el estudio es voluntaria y que son libre de retirarse voluntariamente del estudio en cualquier momento y por cualquier motivo, sin que ninguna de sus decisiones vaya a afectar al trato que reciben de sus médicos y que no van a tener ninguna penalización o pérdida de beneficios a los que tenga derecho. En cualquier caso, el Investigador debe intentar descartar que la razón del abandono voluntario sea un acontecimiento adverso.

El investigador se responsabilizará de obtener el consentimiento informado por escrito de cada uno de los pacientes participantes antes de proceder a cualquier procedimiento médico propio del estudio. La conformidad para participar en el ensayo clínico será expresada al firmar y fechar el documento de Consentimiento Informado específicamente preparado y aprobado para

el estudio. El investigador que dirigió el proceso de obtención del Consentimiento Informado también deberá firmar y fechar en el documento de consentimiento informado.

El Consentimiento informado se firmará por duplicado, una copia original se entregará al paciente o su representante legal y el investigador guardará la otra copia original.

El Gobierno de España decretó el Estado de Alarma para la gestión de la situación de crisis sanitaria ocasionada por COVID-19, mediante el RD 463/2020, de 14 de marzo, donde se limita la libre circulación de las personas. Recalcando la excepcionalidad del momento, y para aquellos casos en que el consentimiento por escrito no se pueda obtener por parte del paciente, el consentimiento verbal por parte de los familiares y / o personas vinculadas al paciente, tras haberlos informado convenientemente, constituye un acto claro de confirmación para proceder a una exploración complementaria, intervención quirúrgica o tratamiento. Cabe resaltar que la información dada, y el posterior consentimiento verbal, deberá quedar bien documentado para su posterior acreditación a la historia clínica del paciente

En el caso del sujeto cuyo consentimiento haya sido otorgado por su representante legal o familiar más cercano, el consentimiento informado por escrito deberá obtenerse de él / ella una vez que recupere la capacidad de consentimiento.

Si surgiera nueva información durante el transcurso del estudio que pudiera afectar al proceso del consentimiento informado, se realizará una revisión de la Hoja de Información al Paciente. Antes de poder ser utilizada, la versión revisada se enviará para su aprobación por el Comité Ético.

Se informará al paciente tan pronto como sea posible si hay nueva información disponible que pudiera afectar a la decisión del paciente a continuar en el estudio. La comunicación de esta información deberá quedar registrada. El paciente, o su representante legal, recibirán una copia de cualquier actualización del Consentimiento Informado o cualquier otra información escrita pertinente.

4.3 PROTECCIÓN DE DATOS DE LOS PACIENTES

Toda la información (personal y clínica) recogida de los pacientes se tratará con arreglo al REGLAMENTO (UE) 2016/679 DEL PARLAMENTO EUROPEO Y DEL CONSEJO de 27 de abril de 2016 relativo a la protección de las personas físicas en lo que respecta al tratamiento de datos personales y a la libre circulación de estos datos, así como el resto de leyes y normativa vigente y aplicable como la Ley Orgánica 3/2018, de 5 de diciembre, de Protección de Datos Personales y garantía de los derechos digitales.

Durante el procedimiento para obtener el Consentimiento Informado, el Investigador solicitará por escrito el permiso del paciente para tener acceso directo a sus datos.

Con este permiso, se podrán examinar, analizar, verificar y reproducir los datos de los pacientes para la evaluación del ensayo clínico.

No obstante, los datos se manejarán de forma anónima, de manera que el paciente al que corresponden no sea identificable. Los datos de los pacientes serán disociados. Se les asignarán números consecutivos según se vayan incluyendo en el estudio, y estos números de identificación (o códigos) se utilizarán en el CRD-e; no debe constar el nombre completo del paciente en los CRD-e. El investigador mantendrá actualizada una lista de identificación de los pacientes con la correspondencia entre nombre, número de historia clínica y el número o código de identificación del paciente para el ensayo clínico.

El monitor del estudio podrá tener acceso a la identidad y datos del paciente en relación con los procedimientos de monitorización del estudio.

Cualquier persona con acceso directo a los datos (Autoridades Reguladoras, Monitores del Ensayo y auditores) tomará todas las precauciones posibles para mantener la confidencialidad de las identidades de los pacientes.

Es responsabilidad del Investigador obtener el consentimiento informado del paciente en el estudio y del Monitor del Ensayo verificar que cada paciente ha otorgado su consentimiento por escrito para permitir este acceso directo.

El Investigador se asegurará de que los documentos que son entregados al Promotor no contienen ni el nombre ni ningún dato identificable del paciente.

4.4 COMITÉ DE ÉTICA DE LA INVESTIGACIÓN CON MEDICAMENTOS

El protocolo de este estudio clínico ha sido revisado y aprobado por el CEIm del Hospital Universitario de Bellvitge (L'Hospitalet del Llobregat, Barcelona).

4.5 SEGURO ESPECÍFICO DE ENSAYOS CLÍNICOS

El tratamiento para COVID-19 utilizado en este ensayo clínico es el tratamiento que se está utilizando en la práctica clínica. De hecho, todos los tratamientos que se están utilizando como antivirales en la práctica clínica de forma empírica (y fuera de ficha técnica) llevan varios años comercializados (apéndice 2).

De acuerdo con lo establecido en la legislación vigente (Real Decreto 1090/2015), este es un **ensayo clínico de bajo nivel de intervención:**

1) El ensayo se refiere a medicamentos autorizados en España y que se están utilizando en la práctica clínica para el manejo de la infección por COVID-19.
2) Aunque ninguno de los tratamientos utilizados en la práctica clínica: hidroxicloroquina (Dolquine®) / cloroquina (Resochin®) / Lopinavir- Ritonavir (Kaletra®) / Interferon (Beta-1-alpha Betaferon®) / Metilprednisolona (Urbason ® , Solu-Moderín ® , Metilprednisolona Normon ®), tocilizumab (Roactemra®), tiene la indicación de la enfermedad objeto de estudio (COVID-19), en la práctica clínica en estos momentos de crisis por el COVID-19 se están utilizado en estos pacientes y en la posología propuesta en este ensayo clínico.
3) Los procedimientos complementarios de diagnóstico o seguimiento no entrañan un riesgo o carga adicional para la seguridad de los sujetos, dado que se sigue los procedimientos de la práctica clínica habitual.

Por todo ello, y de acuerdo al capítulo III, artículo 9 del Real Decreto 1090/2015: "Los daños y perjuicios sobre el sujeto de estudio que pudieran resultar como consecuencia de un ensayo clínico de bajo nivel de intervención no precisarán estar cubiertos por un contrato de seguro o garantía financiera, si los mismos estuvieran cubiertos por el seguro de responsabilidad civil profesional individual o colectivo o garantía financiera equivalente del centro sanitario donde se lleve a cabo el ensayo clínico"; no se ha contratado un seguro *ad hoc* para este ensayo clínico.

4.6 SISTEMA DE CALIDAD, AUDITORÍA E INSPECCIÓN

4.6.1 SISTEMA DE CALIDAD

El Promotor es responsable de mantener los sistemas de garantías y control de calidad durante la ejecución del estudio, mediante Procedimientos Normalizados de Trabajo.

Todas las partes relacionadas con el estudio aceptan el acceso directo a los datos fuente y a los documentos e informes relacionados con el estudio, para ser monitorizados y auditados por el Promotor, y para su inspección por las Autoridades Reguladoras.

La documentación del ensayo clínico deberá ser suficiente para la reconstrucción del transcurso de los acontecimientos (trazabilidad).

4.6.2 AUDITORÍA

Una auditoría consiste en el examen sistemático e independiente de las actividades y documentos relacionados con el estudio para determinar si dichas actividades relacionadas con el estudio fueron realizadas, y si los datos fueron registrados, analizados y comunicados, según lo descrito en el protocolo, los Procedimientos Normalizados de Trabajo del Promotor, la BPC y la normativa de aplicación.

El Investigador permitirá que una persona designada por el Promotor audite las instalaciones y la documentación en los momentos acordados. Los auditores deberán ser independientes del ensayo clínico y de su realización.

4.6.3 INSPECCIÓN

La inspección es el acto por el cual las Autoridades Reguladoras realizan una revisión oficial de los documentos, instalaciones, registros y otros recursos que consideren relacionadas con el ensayo clínico y que pueden localizarse en el Centro del estudio o en las instalaciones del Promotor. El Investigador deberá cooperar con cualquier inspección.

4.7 MODIFICACIONES AL PROTOCOLO

Cualquier cambio en el protocolo durante el estudio se registrará en forma de modificación. Éstas estarán firmadas por el Promotor y el Investigador Principal si procede.

Dependiendo de los contenidos de la modificación y de los requerimientos legales locales, la modificación será presentada para su aprobación por el CEIm correspondiente y a las Autoridades Competentes.

No se hará efectiva ninguna desviación o cambio del protocolo que sea relevante sin previa revisión y emisión de aprobación por parte del CEIm pertinente y por las Autoridades Competentes, excepto en el caso de que sea necesario eliminar un riesgo inminente para los pacientes, o cuando el(los) cambio(s) comprendan sólo aspectos logísticos o administrativos del ensayo clínico.

Si una modificación afectase sustancialmente al diseño del ensayo clínico, a los riesgos potenciales de los pacientes o al tratamiento de los pacientes, se revisará la *Hoja de Información al Paciente* y se presentará al CEIm correspondiente y a las Autoridades Competentes, para su revisión y aprobación.

Cuando un paciente que se encuentre realizando los procedimientos del ensayo clínico se vea afectado por una modificación, se solicitará de nuevo el *Consentimiento Informado* del paciente, utilizando la nueva versión de la *Hoja de Información al Paciente*. Si no se ha finalizado el periodo de inclusión, la nueva versión de la *Hoja de Información al Paciente* se utilizará para obtener el consentimiento de los pacientes que se sigan incluyendo en el estudio.

4.8 CONTRATOS

La participación de los investigadores en este ensayo clínico es voluntaria y desinteresada, es decir, no percibirán cantidad económica alguna por participar en este ensayo clínico.

En el contrato con el centro se reflejará los acuerdos sobre la delegación y distribución de tareas y obligaciones y, si procediera, sobre asuntos financieros.

5. INVESTIGADORES Y ESTRUCTURA ADMINISTRATIVA DEL ESTUDIO

Promotor:

Dr. Xavier SOLANICH Moreno
Servicio de Medicina Interna
Hospital Universitario de Bellvitge
Carrer de la Feixa Llarga, s/n,
08907-L'Hospitalet de Llobregat, Barcelona
Tel.: +34. 93 2602324
Correo electrónico: xsolanich@gmail.com

Investigador Coordinador:

Dr. Xavier SOLANICH Moreno
Servicio de Medicina Interna
Hospital Universitario de Bellvitge
Carrer de la Feixa Llarga, s/n,
08907-L'Hospitalet de Llobregat, Barcelona
Tel.: +34. 93 2602324
Correo electrónico: xsolanich@gmail.com

Monitor Médico:

Dr. Arnau ANTOLÍ Gil
Hospital Universitario de Bellvitge
Carrer de la Feixa Llarga, s/n,
08907-L'Hospitalet de Llobregat, Barcelona
Tel.: +34. 93 2602324
Correo electrónico: aantolig@bellvitgehospital.cat

CEIm

Comité de Ética de la Investigación con Medicamentos (CEIm) del Hospital Universitario de Bellvitge
Carrer de la Feixa Llarga, s/n,
08907-L'Hospitalet de Llobregat, Barcelona
Tel.: +34. 93 260 28 40
Correo electrónico: presidenciaCEIC@bellvitgehospital,cat

Unidad de Soporte a la Investigación Clínica:

USRC-HUB/IDIBELL
Hospital Universitario de Bellvitge
Carrer de la Feixa Llarga, s/n,
08907-L'Hospitalet de Llobregat, Barcelona
Tel.: +34. 93 260 71 14
Correo electrónico: svidela@bellvitgehospital.cat

**CRO – Monitorización /farmacovigilancia**	UICEC – IDIBELL<br>Hospital Universitario de Bellvitge<br>Carrer de la Feixa Llarga, s/n,<br>08907-L'Hospitalet de Llobregat, Barcelona<br>Tel.: +34. 93 260 71 07<br>Correo electrónico: ucicecidibell@bellvitgehospital.cat
**Soporte estadístico**	UBiDi (Unidad de Bioestadística) – IDIBELL<br>Hospital Universitario de Bellvitge<br>Carrer de la Feixa Llarga, s/n,<br>08907-L'Hospitalet de Llobregat, Barcelona<br>Tel.: +34. 93 260 50 00<br>Correo electrónico: ctebe@idibell.cat

Nota: los investigadores participantes en el estudio que aparece en el Apéndice 1, realizarán la actividad investigadora de forma totalmente voluntaria y no percibirán remuneración.

6. INTRODUCCIÓN

6.1 COVID-19

A finales de diciembre de 2019, un brote de una enfermedad emergente (COVID-19) debido a un coronavirus de nueva identificación (llamado SARS-CoV-2) comenzó en Wuhan, China y se extendió por todo el mundo [1,2]. La OMS declaró la epidemia de COVID-19 como una pandemia el 12 de marzo de 2020 [3].

Según un reciente estudio llevado a cabo en China, aproximadamente el 80% de los pacientes presentan una enfermedad leve y la tasa de mortalidad general es aproximadamente del 2.3%, pudiendo alcanzar hasta el 8.0% en pacientes de 70 a 79 años y hasta el 15% en las personas mayores a 80 años [4]. Sin embargo, es muy probablemente que haya un número importante de portadores asintomáticos en la población y, por lo tanto, la tasa de mortalidad probablemente está sobreestimada.

En nuestro entorno, nos enfrentamos a la ola COVID-19 con más de 26000 casos (positivos a COVID-19: al menos 1.612 personas están en UCI y 2.125 ya han recibido el alta) a fecha del 22 de marzo de 2020. Por lo tanto, existe una necesidad urgente de un tratamiento efectivo para tratar a pacientes sintomáticos, y así en consecuencia disminuir la duración de transmisión del virus en la comunidad.

Desafortunadamente, para la enfermedad COVID-19 no hay terapias específicas probadas disponibles, aparte del tratamiento de soporte. Hoy en día, no hay evidencia científica de ensayos clínicos con respecto a la eficacia o seguridad de diferentes medicamentos para tratar pacientes con COVID-19. La administración de cualquier medicamento no probado como "último recurso" supone que el beneficio será más probable que el daño, pero no hay forma de saber si los pacientes se beneficiaron o se vieron perjudicados si no se los compara con un grupo de control concurrente [5]. Es importante destacar que la elección del tratamiento antiviral depende en gran medida del médico a cargo del paciente.

Entre los fármacos candidatos para tratar COVID-19, el reposicionamiento de fármacos antiguos para su uso como tratamiento antiviral o inmunosupresor puede ser una estrategia plausible dado que se conoce el perfil de seguridad, los efectos secundarios, la posología y las interacciones farmacológicas [6,7]. Actualmente, hay más de 600 ensayos registrados en www.clinicaltrials.gov para el tratamiento de pacientes con COVID-19.

A pesar de la falta de evidencia, la urgencia de la atención lleva al hecho de que un gran número de pacientes están recibiendo terapias de uso compasivo y fuera de ficha técnica como cloroquina, hidroxicloroquina, azitromicina, lopinavir-ritonavir, favipiravir, remdesivir, ribavirina, interferón, esteroides, inmunoglobulinas policlonales, inhibidores anti-IL-6 o anti-IL-1, inhibidores de Jak, etc., basados en sus propiedades antivirales o inmunomoduladoras in vitro.

Un artículo reciente reportó un efecto inhibidor de remdesivir (un nuevo fármaco antiviral) y de cloroquina (un fármaco antiguo antipalúdico) sobre el crecimiento de SARS-CoV-2 in vitro [8] y un ensayo clínico temprano realizado en pacientes chinos con COVID-19, mostró que la cloroquina tuvo un efecto significativo, tanto en términos de resultados clínicos como de eliminación viral, en comparación con los grupos de control [9]. Los expertos chinos recomiendan que los pacientes diagnosticados como casos leves, moderados y graves de neumonía por COVID-19 y sin contraindicaciones para la cloroquina, sean tratados con 500 mg de cloroquina dos veces al día durante diez días [10].

Se ha demostrado que la hidroxicloroquina (un análogo de la cloroquina) tiene una actividad antiSARS-CoV *in vitro* [11] y recientemente *in vivo* [12]. El perfil de seguridad clínica de hidroxicloroquina es mejor que el de la cloroquina (sobretodo en su uso a largo plazo)

permitiendo una dosis diaria más alta [13] y con un número menor de interacciones farmacológicas [14].

Otro grupo de fármacos utilizados con antivirales para SARS-CoV-2, son los fármacos antivirales contra el VIH como Lopinavir/Ritonavir (Kaletra®). Si bien, el pasado 18 de marzo fue publicado un ensayo clínico en donde no se observó una eficacia superior de Lopinavir/Ritonavir frente a Standard of care [15].

Los interferones son proteínas producidas por el organismo que le ayudan a combatir contra los ataques al sistema inmunitario, tales como las infecciones víricas. El interferón beta-1alfa (Betaferon®) ha sido propuesto como co-fármaco sobre todo para casos de enfermedades graves.

Algunos pacientes con COVID-19 evolucionan a una lesión pulmonar grave debido a un importante proceso inflamatorio desencadenado por la infección viral [16-18]. Es posible que algunos medicamentos inmunosupresores puedan ser dañinos y pongan a los pacientes en riesgo de una mayor morbilidad / mortalidad asociada con la infección por COVID-19, pero, paradójicamente, podrían ser útiles otros para tratar la inflamación excesiva asociada con una infección viral persistente grave [19].

Algunos artículos sugieren una mejoría de la afectación pulmonar grave de COVID-19 después de la administración de esteroides [20]. Pero el uso de esteroides intravenosos se ha asociado con la eliminación retardada de coronavirus en sangre y pulmones con MERS-CoV [21] y SARS-CoV [22], y los esteroides se asociaron con un riesgo significativamente mayor de mortalidad y eventos adversos en pacientes con influenza [23-25].

Además, un pequeño ensayo realizado en China (ChiCTR2000029765) mostró la efectividad del bloqueo del receptor de IL-6 con Tocilizumab en el tratamiento de pacientes con COVID-19 con neumonía e IL-6 alta. Además, los inhibidores de IL-6 pueden causar una inmunosupresión aún más profunda que los esteroides, aumentando el riesgo de sepsis, neumonía bacteriana, perforación gastrointestinal y hepatotoxicidad [5]. Otro inconveniente de Tocilizumab es su escasez recurrente en algunos hospitales de todo el mundo y su alto costo para los sistemas nacionales de salud.

A pesar de no conocer el equilibrio entre el riesgo y el beneficio de los fármacos antivirales, esteroides y los inhibidores de L-6, ya se están utilizando ampliamente en varios protocolos de atención médica.

La siguiente figura representa el algoritmo de manejo de pacientes infectados por COVI-19 en el hospital Universitario de Bellvitge a fechas 20 de marzo de 2020.

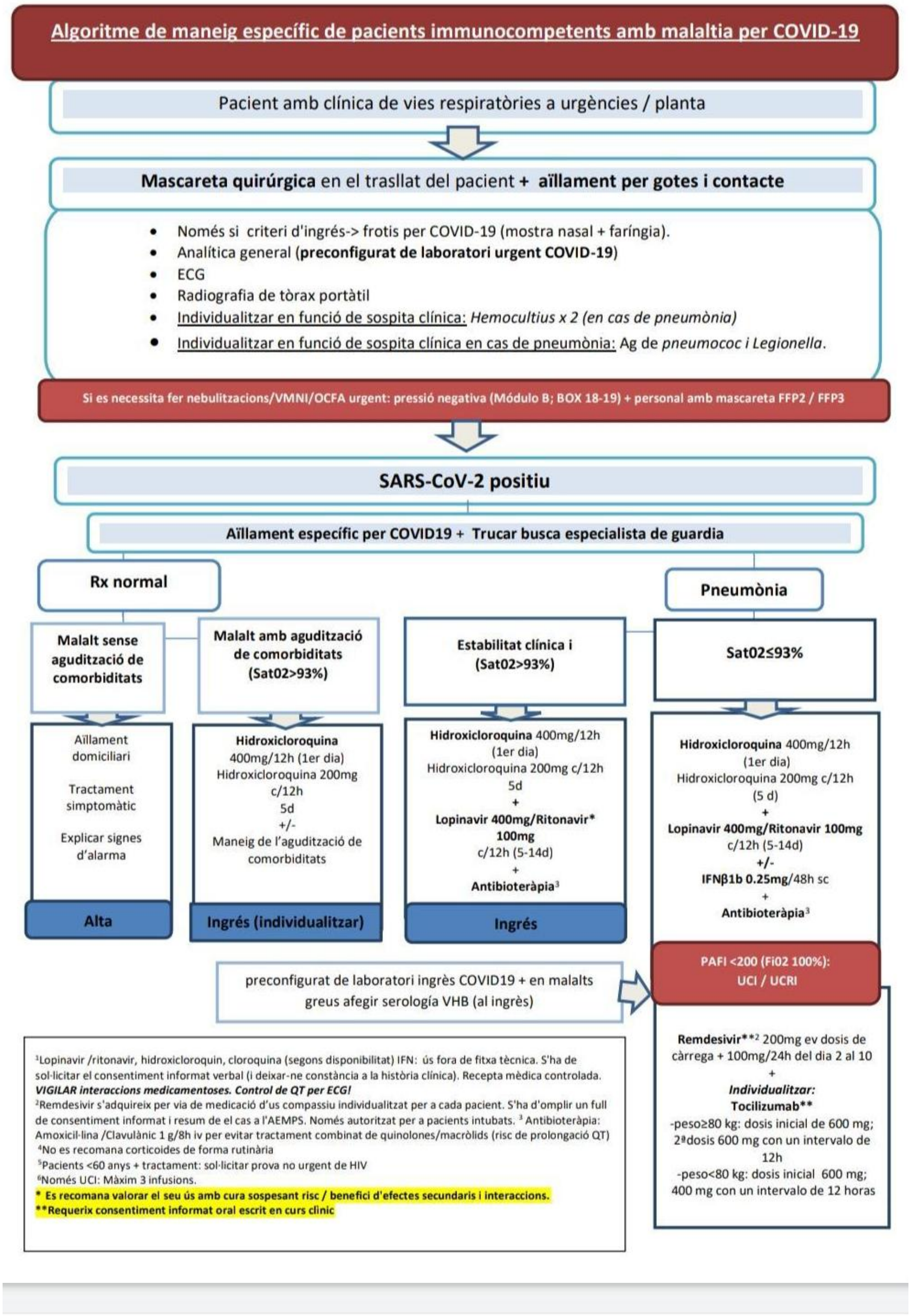

Versió 3.1 20/03/2020

Nuestra hipótesis de trabajo es que la afectación pulmonar grave por SARS-CoV-2 (COVID-19) es secundaria a un proceso inflamatorio excesivo que podría mejorar después de los pulsos de metilprednisolona y la administración de tacrolimus. Dada la rápida expansión de COVID-19 y la emergencia de salud actual en todo el mundo, realizaremos una prueba de concepto en un ensayo clínico aleatorio abierto y uncentrico para evaluar la seguridad y la eficacia de la

metilprednisolona y el tacrolimus junto al tratamiento médico estándar (SoC) versus SoC solo en pacientes con lesión pulmonar grave por COVID-19.

Los glucocorticoides, como la prednisona, son un pilar en el tratamiento de varios trastornos inmunomediados, con múltiples mecanismos de acción que involucran los brazos de inmunidad humoral y celular. Tacrolimus tiene un mecanismo de acción específico, que es la inhibición de la calcineurina en los linfocitos, que conduce a la supresión de citocinas proinflamatorias, incluidos los interferones tipo 1 [26].

La enfermedad grave de COVID-19 presenta un perfil clínico y de citocinas muy similar a enfermedades como la linfohistiocitosis hemofagocítica secundaria [27] o la dermatomiositis clínicamente amiopática (CADM) asociada a anti-melanoma differentiation-associated gene 5 (MDA-5), donde los anticalcineurínicos como el tacrolimus tienen un papel central en su tratamiento [28].

Además, la replicación del coronavirus (CoV) depende de las inmunofilinas, que en cultivos celulares pueden ser inhibidas por Tacrolimus, a concentraciones bajas, no citotóxicas [29,30]. Algunas series de casos sugieren que el tacrolimus puede ejercer un efecto protector en pacientes trasplantados de órganos sólidos afectados de MERS-CoV [19,31].

Por lo tanto, el tacrolimus tiene un efecto inmunosupresor, pero también podría bloquear la replicación viral, y por lo tanto podria tener efectos beneficiosos sobre COVID-19.

6.2 MEDICAMENTO EN INVESTIGACIÓN

Los tratamientos utilizados empíricamente en la práctica clínica: **hidroxicloroquina (**ejemplo, **Dolquine®) / cloroquina (**ejemplo, **Resochin®) / Lopinavir- Ritonavir (**ejemplo, **Kaletra®) / Interferon Beta-1-alpha (**ejemplo, **Betaferon®) / Metilprednisolona** (ejemplo, Urbason ®), **Tocilizumab** (ejemplo, Roactemra®), **no** son medicamentos nuevos, de hecho, están autorizados en España.

En este ensayo clínico se investigará los posibles beneficios de **pulsos de metilprednisolona junto con Tacrolimus** en pacientes hospitalizados con neumonía grave secundaria a COVID-19, y parámetros inflamatorios elevados

Hipótesis:

1. Nuestra hipótesis de trabajo es que la combinación de pulsos de metilprednisolona junto con Tacrolimus en pacientes hospitalizados con neumonía grave secundaria a COVID-19 y parámetros inflamatorios elevados es segura y eficaz para acortar el tiempo hasta estabilidad clínica.

La finalidad del presente proyecto es llevar a cabo un ensayo clínico pragmático, prospectivo, unicéntrico y con asignación aleatoria, para evaluar nuestra hipótesis de trabajo.

7. OBJETIVOS DEL ESTUDIO

Este estudio permitirá la evaluación de la **eficacia** y la **seguridad** de la combinación de pulsos de Metilprednisolona y Tacrolimus para el tratamiento para la neumonía causada por SARS-CoV-2 (COVID-19).

7.1 OBJETIVO PRINCIPAL

Estudiar el tiempo (días) hasta alcanzar la estabilidad clínica tras iniciar la aleatorización en pacientes hospitalizados con neumonía grave secundaria a COVID-19, y elevación de parámetros inflamatorios.

7.2 OBJETIVOS SECUNDARIOS

Objetivos Clínicos:

- Estudiar el tiempo hasta alcanzar un estado afebril durante 48 horas.
- Estudiar el tiempo hasta alcanzar PaO2/FiO2 >400 y/o SatO2/FiO2 >300
- Estudiar el tiempo hasta alcanzar una FR ≤ 24 rpm durante 48 horas.
- Estudiar el tiempo hasta la normalización de dímero D (<250 ug/L)
- Estudiar el tiempo hasta la normalización de la PCR (<5mg/L).
- Estudiar el tiempo hasta la normalización de la ferritina (<400ug/L).
- Cuantificar el estado clínico diariamente según la escala ordinal de la OMS.
- Cuantificar la duración (días) del tratamiento con tacrolimus.
- Cuantificar la duración (días) de la estancia hospitalaria.
- Porcentaje de pacientes que requieren dispositivos de soporte ventilatorio (VMNI y/o VM y/o GNAF)
- Cuantificar la duración (días) que es necesario mantener soporte ventilatorio.
- Describir las alteraciones radiológicas y funcionales pulmonares a los día 56 desde el inicio del tratamiento del ensayo.
- Incidencia de mortalidad por COVID-19 a los 28 y 56 días del inicio del tratamiento del ensayo.
- Incidencia de mortalidad por cualquier causa a los 28 y 56 días del inicio del tratamiento del ensayo.
- Incidencia de recaídas de neumonía por COVID-19 a los 28 y 56 días del inicio del tratamiento del ensayo.
- Estudiar el impacto del tratamiento inmunosupresor en la dinámica viral mediante PCR cuantitativa.
- Analizar el perfil de citocinas ampliado antes del inicio del tratamiento y cada 7 días durante el ingreso.

7.3 OBJETIVOS DE SEGURIDAD

Describir la incidencia de acontecimientos adversos según su gravedad y relación con el tratamiento del ensayo.

8. PLAN DE INVESTIGACIÓN

8.1 DISEÑO DEL ENSAYO CLÍNICO

Ensayo clínico de Fase II, pragmático, con asignación aleatoria, controlado, abierto y unicéntrico para evaluar la eficacia de los pulsos de metilprednisolona y tacrolimus en pacientes hospitalizados con neumonía grave secundaria a COVID-19, y parámetros inflamatorios elevados.

Se realizará un seguimiento a los pacientes hasta aproximadamente 56 ±3 días después de la randomización.

En este ensayo clínico participará:

1. Hospital Universitario de Bellvitge

8.2 POBLACIÓN DEL ESTUDIO Y PROCEDIMIENTO DE RECLUTAMIENTO

Este estudio se llevará a cabo en el Hospital Universitari de Bellvitge que está ubicado en el municipio de L'Hospitalet de Llobregat y es uno de los 5 hospitales de tercer nivel acreditados de Cataluña lo que le permite atender a pacientes de alta complejidad. El HUB actúa como hospital de primer, segundo y tercer nivel. Como primer nivel, el HUB es el hospital general básico para 343.172 habitantes. Además como segundo nivel es referencia para especialidades de complejidad media y alta de una población aproximada de 1,3 millones de habitantes. Finalmente, en las especialidades de alta complejidad (tercer nivel), es hospital de referencia para todo el Eje Sur de Cataluña, aproximadamente 2 millones de habitantes (regiones sanitarias de Metropolitana Sur, Camp de Tarragona y Terres de l'Ebre). A fecha de 23 de Marzo de 2020 tenemos ingresados más de 300 pacientes con insuficiencia respiratoria y/o neumonía secundaria a COVID-19, y la cantidad de ingresos aumenta de forma rápida como en el resto de centros sanitarios españoles. Así pues, los pacientes atendidos en nuestro centro son suficientes para realizar el presente estudio.

En el ámbito de la investigación del HUB forma parte del Institut d'Investigació Biomèdica de Bellvitge (IDIBELL). Se encuentra en el corazón del Biopol de Hospitalet, un clúster que aprovecha la concentración en un solo espacio de hospitales, universidad, centros de investigación y empresas para promover la transferencia de conocimiento y la generación de valor añadido en el ámbito de las ciencias de la salud.

Los Investigadores reclutarán a los pacientes en orden cronológico, cuando cumplan los criterios de selección especificados en el protocolo del estudio (Sección 7.5.1), hasta completar el tamaño muestral requerido. Véase en la Sección 14.1 el cálculo del tamaño de la muestra.

8.2.1 CRITERIOS DE SELECCIÓN

Los pacientes que se incluyan en el estudio **DEBEN** cumplir todos los criterios de inclusión y **NO DEBEN** presentar ninguno de los criterios de exclusión.

8.2.1.1 CRITERIOS DE INCLUSIÓN

- Edad ≥ 18 años
- Infección por COVID-19 confirmada mediante fluorescent RT-PCR

- Infiltrados radiológicos de nueva aparición (bien por radiografía simple de tórax, tomografía axial computarizada o ecografía de tórax) atribuidos a COVID-19,
- Insuficiencia respiratoria (PaO2/FiO2 < 300 o satO2/FiO2 < 220) atribuida a COVID-19,
- PCR > 100 mg/L y/o D-Dimero > 1000 µg/L y/o Ferritina > 1000 ug/L atribuida a COVID-19,
- El sujeto, su representante legal o familiar más cercano (en caso de incapacidad del sujeto por gravedad de la situación clínica) otorgan el consentimiento informado

  Si el representante legal o un familiar más cercano están en cuarentena debido a la emergencia por COVID-19, el consentimiento informado se proporcionará oralmente mediante una llamada telefónica y se documentará en la historia clínica del sujeto.
  En el caso del sujeto cuyo consentimiento haya sido otorgado por su representante legal o familiar más cercano, o bien el paciente haya otorgado su consentimiento de manera oral, el consentimiento informado por escrito deberá obtenerse de él / ella una vez que recupere la capacidad de consentimiento.

8.2.1.2 Criterios de exclusión

- Muerte inminente (expectativa de vida ≤ a 24h).
- Filtrado glomerular ≤ 30 ml/min /1,73 m2 (estimado según la ecuación CKD-EPI)
- Leucopenia ≤ 4000 células/µL
- Infecciones concomitante potencialmente graves.
- Contraindicación para el uso de tacrolimus según la ficha técnica del producto.
- Reacciones adversas conocidas al tratamiento
- Haber participado en un ensayo clínico los últimos 3 meses.

8.3 Asignación aleatoria y enmascaramiento

La lista de asignación aleatoria será generada por ordenador en bloques de 10.

El proceso de asignación aleatoria será por vía electrónica mediante el propio CRD-e. Los pacientes serán asignados a los grupos de estudio en el momento de ser incluidos en el estudio.

Dado que se trata de un ensayo clínico abierto, no se enmascarará la medicación objeto de estudio.

8.4 Criterios de valoración

8.4.1 Criterios de valoración de la eficacia

8.4.1.1 Criterio principal (para el objetivo principal)

Tiempo (días) hasta alcanzar la estabilidad clínica después de iniciar el tratamiento del ensayo, en pacientes hospitalizados con neumonía grave secundaria a COVID-19 y parámetros inflamatorios elevados.

Se definirá **éxito del tratamiento** si los pacientes cumplen los criterios de estabilidad clínica durante 48 horas.

Se define **fracaso del tratamiento:**

- paciente que no cumple los criterios de estabilidad clínica a los 56 días de haber iniciado el tratamiento, o
- paciente que presenta una reacción adversa de grado 3 o 4 atribuida al tratamiento del ensayo, o
- paciente que fallece tras haber sido incluido en el ensayo clínico.

Si el paciente es considerado "fracaso terapéutico", el paciente finaliza el tratamiento del ensayo clínico en ese momento, y pasaría a ser tratado según el criterio del médico a cargo del paciente.

8.4.1.2 Criterios secundarios (para los objetivos secundarios)

Clínicos:

- Tiempo ('días') hasta alcanzar un estado afebril durante 48 horas.
- Tiempo ('días') hasta alcanzar una PaO2/FiO2 >400 y/o SatO2/FiO2 >300 durante 48 horas.
- Tiempo ('días') hasta alcanzar una FR ≤ 24 rpm durante 48 horas
- Tiempo ('días') hasta la normalización del dímero D (<250 ug/L),
- Tiempo ('días') hasta la normalización de IL-6 (<5mg/L).
- Tiempo ('días') hasta la normalización de Ferritina (<400ug/L)
- Cambio (porcentaje) de la carga viral (PCR) antes del inicio del fármaco respecto al día 7.
- Número de pacientes que requieren dispositivos de soporte ventilatorio no invasivo (VMNI, GNAF…)
- Número de pacientes que requiere dispositivos de soporte ventilatorio invasivo (VM)
- Tiempo ('días') en unidad de cuidados intensivos.
- Tiempo ('días') en unidad de cuidados semi-intensivos.
- Número de días de estancia hospitalaria (desde el día del inicio del tratamiento del ensayo hasta el alta hospitalaria)
- Descripción del estadio clínico según la OMS durante el periodo hasta el alta hospitalaria.
- Número de pacientes que alcanzan un estadio clínico ≤2 tras 10 días del inicio del tratamiento o al alta, lo que suceda antes.
- Número de pacientes que alcanzan la estabilidad clínica a los 10 días del inicio del tratamiento o al alta, lo que suceda antes.
- Valor medio de cada uno de los valores analíticos (dimero D, IL-6, ferritina) a los 10 días o al alta, lo que suceda antes.
- Número de días con el tratamiento del ensayo.
- Cambio de la carga viral cuantitativa mediante real-time RT-PCR entre las muestras antes del inicio del fármaco y las realizadas semanales durante la hospitalización.
- Cuantificar las citocinas estudiadas en el perfil de citocinas ampliado antes del inicio del tratamiento, y semanalmente durante la hospitalización.
- Eficacia a largo plazo (a los 28 y 56 días del inicio del tratamiento del ensayo) se evaluara midiendo si se mantiene la estabilidad clínica y la incidencia de recaídas de neumonía por COVID-19.

- Describir las alteraciones radiológicas (radiografía simple, TAC tórax, pruebas funcionales) a los 56±3 días del inicio del tratamiento del ensayo.

Mortalidad:

- Incidencia de mortalidad por COVID-19 a los 28 y 56 días del inicio del tratamiento del ensayo.
- Incidencia de mortalidad por cualquier causa a los 28 y 56 días del inicio del tratamiento del ensayo.

8.4.1.3 CRITERIOS DE VALORACIÓN DE LA SEGURIDAD

- Incidencia de acontecimientos adversos según su gravedad y relación con el tratamiento.

8.5 PERIODOS Y DURACIÓN DEL ESTUDIO

Inicio del estudio: El estudio comenzará con la inclusión del primer paciente ("First Patient –First Visit").

Periodo de reclutamiento de pacientes: El periodo de inclusión de pacientes finalizará con la inclusión del último paciente, de acuerdo con el número de pacientes indicados en el cálculo del tamaño de la muestra. Se estima que el periodo de reclutamiento sea aproximadamente de 3 mes.

Periodo de participación de cada paciente: Cada paciente finalizará su participación en el estudio después de habérsele realizado todas las pruebas a evaluar en el estudio, o cuando sea retirado prematuramente (independientemente del motivo: propia voluntad, etc.). La participación de cada paciente en el estudio durará un máximo de 56±3 días desde la randomización.

Finalización del estudio: El estudio finalizará cuando finalice la evaluación (visita a los 56±3 días desde la randomización) del último paciente incluido en el estudio ("Last Patient-Last Visit").

Fecha estimada de inicio del estudio: El estudio se iniciará el 1 de Abril de 2020.

Fecha estimada de finalización del estudio: Se estima que el periodo de reclutamiento durará 3 meses, o el tiempo que se tarde en reclutar el tamaño de muestra necesario que lo marcará el análisis secuencial, necesario para evaluar los resultados con suficiente potencia estadística. Más 56±3 días desde la randomización del último paciente incluido. Por lo tanto, aproximadamente, la fase clínica del estudio finalizará en agosto 2020.

Después de la finalización de la fase clínica, se requieren un mes para la depuración de los datos y la realización de los análisis estadísticos, y de nueve meses para el informe final.

Por tanto, **la duración total del estudio sería hasta abril de 2021**.

8.6 RETIRADA DE SUJETOS

La participación en el estudio es voluntaria y los sujetos pueden retirarse en cualquier momento sin tener que dar explicaciones y sin que ello suponga un menoscabo de la atención sanitaria que reciban en el futuro.

Por su parte, el investigador deberá retirar a un sujeto del estudio:

- Acontecimiento adverso grave e inesperado.
- Criterio clínico.
- Solicitud de retirada por parte del paciente.
- Violación del protocolo.

- Pérdida de seguimiento.
- Embarazo durante el estudio.

No se consideran criterios de retirada las complicaciones relacionadas con de neumonía por SARS-CoV-2 en pacientes que se encuentren sin ventilación mecánica.

Cuando un sujeto se retire del estudio, el investigador registrará en la Historia Clínica el motivo o los motivos de la retirada en los documentos originales y en la página correspondiente del cuaderno de recogida de datos electrónico (CRD-e). Siempre que sea posible, los sujetos que se retiren del estudio prematuramente se someterán a todas las valoraciones de la visita de EoT. Si la participación de un sujeto se suspende por un acontecimiento adverso grave (AAG), el acontecimiento se seguirá hasta su resolución o estabilización. Es obligatorio obtener los datos de seguimiento de los pacientes retirados por AAG. En cualquier caso, se hará lo posible por llevar a cabo los procedimientos de seguridad y seguimiento que se especifican en el protocolo.

A todos los pacientes retirados del estudio se les completará la visita ToC (visita a los 56±3 días desde la randomización). Los pacientes retirados no serán reemplazados.

8.7 CUIDADO DE LOS PACIENTES TRAS FINALIZAR EL ESTUDIO

Los pacientes que **finalicen su participación** en este estudio, de acuerdo con la evolución de su neumonía por SARS-CoV-2 en la última visita del estudio (ToC), si necesitan continuar en tratamiento a criterio de su médico, entonces, continuarán con las pruebas médicas y tratamientos que les prescriba su médico de acuerdo a su práctica clínica habitual.

9. DESCRIPCIÓN DEL TRATAMIENTO

9.1 DOSIS, INTERVALO, VÍA, FORMA DE ADMINISTRACIÓN Y DURACIÓN DE LOS TRATAMIENTOS DE ENSAYO

Los pacientes incluidos en el estudio serán asignados de manera aleatoria (1:1) a uno de los 2 grupos:

Grupo experimental: pulsos de metilprednisolona 120mg/día durante 3 días consecutivos junto con tacrolimus* a la dosis necesaria para conseguir unos niveles plasmáticos de entre 8-10 ng/ml. Se suspenderá el tratamiento en el momento que se consiga la estabilidad clínica. Además estos pacientes podrán recibir todos los tratamientos que se consideren necesarios para su manejo clínico.

^ No se administraran los bolus si los 7 días previos a la aleatorización el paciente ha recibido ≥ 3 bolus de metilpredisolona (o dexametasona ≥ 20mg/día durante ≥ 3 dias). Si ha recibido menos de 3 bolus se completaran hasta un número total de 3

* Tacrolimus (Modigraf o Advagraf): Dosis inicial recomendada 0.05mg/kg cada 12 horas y ajustar según niveles. Si toma hidroxicloroquina 0.1mg/kg i ajustar según niveles igual. Si toma Lopinavir dosis inicial de 0.2mg cada 48 horas. En caso de Insuficiencia renal y/o insuficiencia hepática administrar las dosis descritas anteriormente sin modificaciones. Si requiere tratamiento endovenoso o tiene dudas del tratamiento contactar con farmacia (Dra Núria Padulles).

Grupo control: el régimen de tratamiento estándar consistirá en administrar el tratamiento antiviral elegido por el médico responsable del paciente, además de todos los tratamientos que se consideren necesarios para su manejo clínico.

9.2 PRECAUCIONES CON LOS MEDICAMENTOS DE ESTUDIO.

Según especificado en la ficha técnica:

<u>Tratamiento concomitante</u>: Se permitirá el uso de cualquier fármaco si se considera necesario para su manejo clínico. Toda medicación concomitante quedará reflejada en la historia clínica (detallando el producto, dosis, vía, días de administración, motivo del tratamiento). En el CRD-e se recogerá la medicación: hidroxicloroquina, cloroquina, lopinavir-ritonavir, interferon beta 1b, antibióticos, corticoesteroides, otros inmunosupresores.

<u>Criterios de retirada del tratamiento de estudio:</u>

Tacrolimus (Advagraf®, Modigraf®) se suspenderá según lo especificado en la ficha técnica (apéndice 2) y/o si se producen los siguientes supuestos:

-Infección grave o potencialmente grave.

- Necesidad de ventilación mecánica invasiva y/o ECMO.
- Efecto adversos graves relacionados con la medicación (HTA refractaria, descenso superior al 50% del FG respecto al basal, QTc> 550 mseg o taquicardia ventricular).
- Otros acontecimientos adversos graves relacionados con la medicación.
- Criterio clínico.
- Solicitud de retirada por parte del paciente.
- Violación del protocolo.
- Pérdida de seguimiento.

- Embarazo durante el estudio.

Metilprednisolona (Urbason®, Solu-Moderín®) se suspenderá según lo especificado en la ficha técnica (apéndice 2) y/o si se producen los siguientes supuestos:

-Infección grave o potencialmente grave.

-Otros acontecimientos adversos graves relacionados con la medicación.

-Criterio clínico.

- Solicitud de retirada por parte del paciente.

-Violación del protocolo.

-Pérdida de seguimiento.

El servicio de farmacia de Bellvitge se encargaría de que la dosis de tacrolimus sea la correcta según las especificaciones del protocolo.. El grupo de tratamiento recibirá la tacrolimus según la dosis descrita en apartado 8.1. Se administrará 1 hora antes de comer para obtener la máxima absorción. Las concentraciones en sangre se determinarán con métodos de inmunoensayo. Las concentraciones en sangre de tacrolimus se analizarán 24 horas después de la última dosis de tacrolimus, justo antes de la siguiente administración. Esta determinación se realizará cada 2 días mientras el paciente reciba el fármaco para mantener unos niveles plasmáticos entre 8 y 10 ng/ml. El servicio de farmacia del HUB se encargará de ajustar la dosis del fármaco.

El tacrolimus se metaboliza a través del CYP3A4 hepático y de la pared intestinal. El uso concomitante de sustancias conocidas por inhibir o inducir CYP3A4 puede afectar el metabolismo de tacrolimus y, por tanto, elevar o disminuir su concentración sanguínea. Fármacos que se están utilizando para tratar la infección por COVID-19, como por ejemplo los inhibidores de la proteasa para el VIH (ritonavir) inhiben el CYP3A4. Por lo tanto, es de esperar un aumento de las concentraciones de tacrolimus en sangre. Para evitar complicaciones, se dará una dosis inicial baja (0.2mg cada 48 horas) que se ajustará según los niveles plasmáticos. Habrá que revisar también otros fármacos que puedan influir en el metabolismo de tacrólimus. Una de las complicaciones que más preocupa a los investigadores es la prolongación del intervalo QT (trastorno también descrito con lopinavir / ritonavir). Para evitar arritmias derivadas de este trastorno, se evaluará el intervalo QT durante el estudio mediante un ECG cada 48 horas y se retirará el fármaco del estudio si es superior a 550mseg. Mediante los controles de constantes vitales diarios y las analíticas rutinarias se evaluarán otros potenciales efectos adversos del fármaco. Se ha descrito un mayor riesgo de infecciones, incluidas infecciones oportunistas, pero los investigadores no esperamos que los pacientes desarrollen este tipo de complicaciones ya que el fármaco se administrará durante un corto período de tiempo.

Medidas para valorar el cumplimiento: La administración de los medicamentos en investigación serán recogidos en la historia clínica y en el CRD-e.

9.3 GESTIÓN DE LA MEDICACIÓN DEL ESTUDIO.

La medicación en investigación (Metilprednisolona / Tacrolimus) será aportada por el hospital de acuerdo con la práctica clínica habitual.

10. PARÁMETROS Y PRUEBAS DE EVALUACIÓN

10.1 CLÍNICOS

Es responsabilidad del Investigador principal de cada centro la valoración clínica del paciente.

Diagnóstico de neumonía por SARS-CoV-2: Paciente hospitalizado que presenta infiltrados radiológicos de nueva aparición (bien por radiografía simple de tórax, tomografía axial computarizada o ecografía de tórax) y con detección (PCR) de SARS-CoV-2 positiva.

Estabilidad clínica: la estabilidad clínica se define cuando el paciente presenta todos los siguientes criterios durante 48 horas consecutivas:

- Afebril: temperatura corpórea ≤ 37,5ºC sin uso de antitérmicos durante 48 horas
- PaO2/FiO2 >400 y/o SatO2/FiO2 >300
- Frecuencia respiratoria ≤ 24 rpm

Estado clínico: se define de acuerdo con la siguiente escala ordinal de la OMS (reference https://clinicaltrials.gov/ct2/show/NCT04280705):
7) muerte
6) Hospitalizado, con ventilación mecánica invasiva o ECMO
5) Hospitalizado, con ventilación no invasiva o dispositivos de oxígeno de alto flujo
4) Hospitalizado, que requiere oxígeno suplementario de bajo flujo
3) Hospitalizado, que no requiere oxígeno suplementario - requiere atención médica continua (COVID-19 relacionado o no)
2) Hospitalizado, que no requiere oxígeno suplementario, ya no requiere atención médica continua
1) No hospitalizado

Se recogerán datos de filiación (código anonimizado, edad, sexo), enfermedades o condiciones debilitantes presentes en el índice de Charlson (https://www.mdcalc.com/charlson-comorbidity-index-cci), constantes vitales (temperatura, satO2 basal, frecuencia respiratoria y tensión arterial), índices para evaluar la gravedad de la neumonía [PSI (http://www.samiuc.es/pneumonia-severity-index-psi/) y CURB-65 (http://www.semergencantabria.org/calc/cpcalc2.htm)], tratamientos concomitantes, efectos secundarios atribuidos a la medicación del ensayo o a otros tratamientos, fecha del alta hospitalaria, mortalidad atribuida a la medicación del ensayo o a otros tratamientos.

10.2 EXPLORACIONES COMPLEMENTARIAS

Se recomienda la realización de un ECG 3 veces por semana.

Se realizará una de rutinaria 3 veces por semana. El HUB dispone de las instalaciones y aparataje necesario para procesar i analizar las muestras de los pacientes con COVID-19. Se ha creado un preconfigurado en el SAP del HUB con todas las variables del estudio. Se realizará una analítica cada 48 horas con hemograma [recuento de glóbulos blancos (WBC), recuento de linfocitos (LYM), recuento de neutrófilos (NEU)] mediante analizador Sysmex XN2000, bioquímica [Aspartate aminotransferasa (AST), Alanina aminotransferasa (ALT), bilirrubina (BIL), gamma-glutamil transpeptidasa (GGT), fosfatasa alcalina (FA), Lactato Deshidrogenasa (LDH), Ferritina, Glucosa (Glu), Urea, Creatinina (Cr), Calcio (Ca), albúmina (ALB), creatinina cinasa (CK), C-reactive protein

(CRP), NT–proBNP, troponinas (TN), procalcitonina (PCT) y IL-6] que se medirán con un analizador se medirán con un analizador Cobas 6000/8000 (Roche Diagnostics) que posee módulos de espectrofotometría y de inmunoquímica con detección electroquimioluminiscente. La coagulación [el D-Dímero (DD), tiempo de protrombina (PT), Fibrinógeno (FIB), tiempo de tromboplastina parcial activada (APTT)] se determinará mediante el analizador de ACLTOP 550 (Werfen).

Los niveles de tacrolimus se medirán mediante cromatografía líquida de alta y rápida eficacia (UHPLC) acoplada a la espectrometría de masas en tándem (MS/MS)

Se dispone de Biobanco del HUB que cumple con todos los requisitos de la legislación vigente. Se encargará del procesamiento y almacenamiento (suero congelado) de muestras de pacientes con COVID-19. Se analizaran las muestras de los 84 pacientes antes de iniciar el tratamiento del ensayo y 7 días después del inicio de su administración. Tras obtener todas las muestras, se enviaran al Centro de Diagnóstico Biomédico del Laboratorio de Immunologia del Hospital Clínic de Barcelona para realizar una técnica de cuantificación multiple de citocinas por Luminex en los sueros de los pacientes infectados con COVID19. Analizaremos la concentración de las siguientes citocinas: IL-1alpha, IL-1beta, IL-1RA, IL-2, IL-4, IL-6, IL-8, IL-7, IL-10, IL-18, TNF -alpha, IFN-alpha, IFN-beta, IFN-gamma, CXCL10 / IP10, CXCL9 / MIG, MCP-1 / CCL2, MIP-1alpha, G-CSF e IL-2R / CD25.

Además se determinaran parámetros virológicos (PCR cuantitativa) para evaluar el impacto del tratamiento inmunosupresor en la dinámica viral. Los estudios microbiológicos se realizarán en el Servicio de Microbiología del Hospital de Bellvitge

11. PROCEDIMIENTOS Y CALENDARIOS

11.1 NÚMERO E INTERVALO DE VISITAS DEL ESTUDIO

Hasta que el paciente (o su representante legal o familiar más cercano, si procede) no hayan dado su consentimiento verbal, no se le incluirá en el estudio (Visita basal: día 0, inclusión del paciente, inicio del tratamiento) ni tampoco se le realizará ninguna prueba específica para el estudio.

Se seguirá a los pacientes hasta los 56±3 del inicio del tratamiento (fin de estudio).

Selección de los pacientes: los pacientes serán seleccionados en el ingreso en planta. Los miembros del equipo investigador valoraran la inclusión del paciente en el estudio si el médico tratante lo solicita.

Calendario de visitas:

- **Visita basal (día 0, inclusión del paciente, inicio del tratamiento):** en esta visita se realizará una evaluación clínica y se confirmará que el paciente cumple todos los criterios de inclusión y ninguno de exclusión. Se informará al paciente sobre el estudio y se le entregará la hoja de información y el consentimiento informado para su firma. Se procederá a la asignación del tratamiento y se iniciará el mismo. Se registrarán datos demográficos, clínicos y analíticos, así como la medicación concomitante.

- **Durante el ingreso hospitalario (número de días variable según el período de ingreso):** se recogerá diariamente de la historia clínica todas las variables relacionadas con los criterios de estabilidad clínica (temperatura, satO2, FiO2, FR y tensión arterial). Se realizará un ECG i una analítica cada 48 horas con los parámetros que se especifican en el apartado de laboratorio.

- **Visita al alta del ingreso hospitalario (día del alta hospitalaria):** se registrará el día de alta hospitalaria: los días de ingreso, días de ventilación mecánica, días con tratamiento antiviral, las complicaciones relacionadas o no con COVID-19, así como la presencia de acontecimientos adversos.

  Se enviará al paciente a su domicilio y se le facilitará las medidas a seguir en su domicilio en régimen de confinamiento que debe cumplir durante 14 días. Se le informará que hacia el día 28 y 58 de haber iniciado el tratamiento del ensayo será visitado para hacer un control de la evolución de la enfermedad.

- **Visita al día 28 ±3 del inicio del tratamiento:** Se evaluará si los pacientes han realizado el tratamiento según el protocolo, si cumplen los criterios de estabilidad clínica, si han presentado una recaída o empeoramiento de la enfermedad, así como la presencia de acontecimientos adversos. Esta visita será presencial..

- **Visita al día 56 ±3 del inicio del tratamiento (fin de estudio):** Se evaluará si los pacientes han realizado el tratamiento según el protocolo, si cumplen los criterios de estabilidad clínica, si han presentado una recaída o empeoramiento de la enfermedad, así como la presencia de acontecimientos adversos. Esta visita será presencial..

La duración del proyecto será de 1 año; alrededor de cuatro meses para el trabajo de campo con el reclutamiento e inclusión de los pacientes más posteriormente el periodo de análisis de resultados y de elaboración de los trabajos resultantes (incluido informe final).

1. Solicitud de autorización al CEIm del centro promotor (Hospital de Bellvitge).

2. Reclutamiento de pacientes: Abril 2020 – Junio 2020.
Visita diaria a las plantas del hospital por miembros clínicos del equipo investigador para estimular la inclusión de pacientes.
Obtención del consentimiento informado antes de la inclusión de cada paciente.
Asignación aleatoria de los pacientes y administración del tratamiento asignado (experimental o control).

3. Recogida de datos: Abril 2020 – Agosto 2020.
Introducción de los datos de cada paciente en la base de datos realizada específicamente para este proyecto. Se llevará a cabo además el proceso de depuración de datos para aumentar la calidad de los mismos y disminuir posibles errores.

4. Finalizado el trabajo de campo se realizarán los análisis estadísticos pertinentes. A todos los investigadores se les presentarán los resultados para su discusión, generación de ideas y propuestas: Setiembre 2020.

5. Presentación de los resultados en el 41º Congreso de la Sociedad Española de Medicina Interna (SEMI), elaboración de los artículos con los resultados del estudio, elaboración de las memorias anuales y la memoria final del proyecto: Octubre 2020-Abril 2021.

En la siguiente tabla se detallan los procedimientos que se realizarán en cada visita.

	**Visita Basal**	**Durante el ingreso hospitalario**	**Visita al alta del ingreso hospitalario**	**Visita al día 28 ±3 del inicio del tratamiento (End of treatment, EoT)**	**Visita al dia 56±3 del inicio del tratamiento (ToC, fin de estudio)**
**Día**	Día 0, inclusión del paciente, inicio del tratamiento	Día +1, +2…. desde el inicio del tratamiento	Día del alta hospitalaria	Día 28 ±3 del inicio del tratamiento	Día 56 ±3 del inicio del tratamiento
**Criterios de inclusión / exclusión**	✓				
**Firma consentimiento informado**	✓				
**Asignación aleatoria**	✓				
**Datos demográficos, Comorbilidades**	✓				
**Datos específicos de la enfermedad COVID**	✓				
**Constantes (tº, sat.O2, FR, TA, FC)**	✓	✓	✓	✓	✓
**Escala ordinal (OMS)**	✓	✓	✓	✓	✓
**Exploración física**	✓	✓	✓	✓	✓
**Analítica general: hemograma, ionograma, función renal y hepática, coagulación**	✓	✓	✓	✓	✓
**Niveles tacrolimus**		✓	✓		
**Citocinas**	✓	✓		✓	✓
**Carga viral (sangre)**	✓	✓		✓	✓
**PCR orofaringea SARS-CoV-2**	✓			✓	✓
**Estabilidad clínica**	✓	✓	✓	✓	✓
**radiografía simple o tomografía axial computarizada o ecografía de tórax**	✓	✓		✓	✓
**Medicación a estudio**	✓	✓	✓	✓	✓
**Registro AA**	✓	✓	✓	✓	✓
**Registro medicación concomitante**	✓	✓	✓	✓	✓

Durante el estudio, se realizarán analíticas y otras pruebas complementarias según criterios clínicos del equipo responsable.

12. DOCUMENTACIÓN DE LOS DATOS DEL ESTUDIO

12.1. CUADERNO DE RECOGIDA DE DATOS

Los datos serán recogidos por los investigadores de cada centro en una base de datos específicamente elaborada para el estudio (en la web REDCap).

Se recogerán de forma exhaustiva datos que incluirán la fecha y hora de la asignación aleatoria (y administración de la primera dosis del tratamiento experimental), datos demográficos, historia clínica, complicaciones relevantes, datos clínicos del proceso agudo, agente etiológico, cualquier medicamento administrado, así como los resultados de los análisis bioquímicos.

En ambos grupos, los pacientes serán controlados cada día durante la hospitalización y se calculará y registrará el tiempo hasta la estabilidad clínica en ambos grupos de tratamiento. La estabilidad clínica se definirá y calculará desde el momento del ingreso y de acuerdo con los criterios descritos previamente.

Las variables secundarias (secondary end points) incluirán el tiempos hasta normalización de variables clínicas (temperatura, saturación de oxigeno, frecuencia respiratoria), el tiempo hasta normalización de variables analíticas (dímero D, PCR, ferritina), evolución del escala ordinal de la OMS, evolución de la afectación pulmonar, evolución de carga viral, evolución del perfil de citocinas, estancia hospitalaria, requerimiento de suporto ventilatorio invasivo y no invasivo, los eventos adversos, las recaídas de neumonía por COVID-19, y la mortalidad.

Se recogerán en el CRD-e los AA cuando se considere que existe una posible relación de causalidad con el medicamento en investigación, cuando sean graves o cuando sean de especial interés.

La monitorización del estudio será llevada a cabo por miembros de la UICEC del IDIBELL.

12.2. ARCHIVO DEL INVESTIGADOR

El Investigador mantendrá los archivos de los documentos esenciales del estudio, tal como se indica en la BPC, en las recomendaciones de la ICH y en las normativas locales.

El Archivo del Investigador estará a disposición en las visitas de monitorización, de auditoría y de inspección.

El Promotor informará por escrito al Investigador/Centro sobre la necesidad de la conservación de los archivos durante un periodo mínimo de 25 años, y también les notificará del momento desde que el mantenimiento de los archivos relacionados con el estudio ya no es necesario.

12.3. CONFIDENCIALIDAD DE LOS RESULTADOS DEL ENSAYO CLÍNICO

Los resultados de este ensayo clínico son confidenciales y no podrán ser transmitidos a terceros de ninguna forma o manera sin permiso por escrito del Promotor. Todas las personas involucradas en el ensayo clínico están ligadas por esta cláusula de confidencialidad de acuerdo al REGLAMENTO (UE) 2016/679 DEL PARLAMENTO EUROPEO Y DEL CONSEJO de 27 de abril de 2016 relativo a la protección de las personas físicas en lo que respecta al tratamiento de datos personales y a la libre circulación de estos datos, así como el resto de leyes y normativa vigente y aplicable como la Ley Orgánica 3/2018, de 5 de diciembre, de Protección de Datos Personales y garantía de los derechos digitales. Por tanto, los datos del paciente se pseudoanonimizarán.

12.4. Política de publicación

El Promotor se compromete a publicar los resultados del estudio, tanto si fueran positivos como si fueran negativos.

Además del envío a una revista médica, la presentación en congresos y otros foros científicos también se consideran publicaciones.

Los borradores de los manuscritos para publicaciones se prepararán en cooperación entre el Promotor y los Investigadores. Sólo serán posibles las publicaciones conjuntas si ambas partes están de acuerdo. Las decisiones editoriales se tomarán conjuntamente por el Promotor y los Investigadores.

Los Investigadores participantes no podrán publicar separadamente los resultados que hayan obtenido de este ensayo clínico.

El Promotor se reserva el derecho de revisar cualquier manuscrito relacionado con el ensayo clínico antes de que sea presentado para su publicación. Ninguna de las partes tiene el derecho de prohibir la publicación a no ser que se pruebe que esta publicación afecta a posibles derechos de patente.

El Promotor se reserva el derecho a posponer cualquier publicación programada hasta la aprobación de cualquier solicitud de patente.

12.5. Informe final

Se preparará un informe final que recoja todos los datos y resultados obtenidos en el estudio.

El Promotor, dentro de un plazo de un año después de notificar la finalización del ensayo clínico, entregará el resumen del informe del ensayo clínico a las Autoridades Competentes y al CEIm.

También se entregará a los Investigadores el informe final o un resumen, según se acuerde.

13. SEGURIDAD DEL MEDICAMENTO DE ESTE ESTUDIO

Uno de los objetivos de este estudio es comprobar la seguridad de la combinación de tratamiento utilizado en este ensayo clínico.

13.1 DEFINICIONES

13.1.1. ACONTECIMIENTO ADVERSO / REACCIÓN ADVERSA

Un **acontecimiento adverso** (AA) es cualquier incidencia perjudicial para la salud en un paciente o sujeto de ensayo clínico tratado con un medicamento, aunque no tenga necesariamente relación casual con dicho tratamiento.

Una **reacción adversa** (RA) es toda reacción nociva y no intencionada a un medicamento en investigación, con independencia de la dosis administrada.

13.1.2. ACONTECIMIENTO ADVERSO GRAVE Y REACCIÓN ADVERSA GRAVE

Se consideran **AA graves** (AAG) o **RA graves** (RAG) aquellos que a cualquier dosis:

- Provoquen la muerte. Nota: la muerte es una posible evolución de la infección por SARS-CoV-2
- Pongan en riesgo la vida.
- Requieran la hospitalización del paciente o la prolongación de una hospitalización ya existente.
- Provoquen invalidez o incapacidad permanente o importante.
- Provoquen una anomalía o malformación congénita.
- Se considere como médicamente relevante.

Se dejará a criterio médico la decisión de considerar otras situaciones como graves, como pueden ser acontecimientos médicos relevantes que requieran una intervención para prevenir alguno de los desenlaces que se han señalado arriba.

13.1.3. REACCIÓN ADVERSA INESPERADA

Se considera inesperada cualquier RA cuya naturaleza, gravedad o consecuencias no se corresponde con la información de seguridad de referencia (ISR).

13.1.4. REACCIÓN ADVERSA GRAVE E INESPERADA

Se considera RAG e inesperada (RAGI) aquella cuya naturaleza, gravedad o consecuencias no se corresponde con la información recogida en las fichas técnicas de los fármacos utilizados (sección 10.2. anexo 2).

13.1.5. CAUSALIDAD CON EL MEDICAMENTO EN INVESTIGACIÓN

<u>AA Relacionado:</u> La relación temporal del AA con la medicación en estudio indica una relación causal posible y no puede ser explicado por factores tales como el estado clínico del paciente u otras intervenciones terapéuticas.
<u>AA no relacionado:</u> La relación temporal del AA con la medicación en estudio indica una relación causal improbable, o bien otros factores (medicación o condiciones concomitantes), otras intervenciones terapéuticas proporcionan una explicación satisfactoria para el AA.

13.1.6. INFORMACIÓN DE SEGURIDAD DE REFERENCIA

En este estudio, la información de seguridad de referencia de los medicamentos a estudio corresponde a las fichas técnicas de referencia (apéndice 2):

- Metilprednisolona: ficha técnica de referencia URBASON®: https://cima.aemps.es/cima/pdfs/es/ft/34023/34023_ft.pdf
- Metilprednisolona: ficha técnica de referencia SOLU-MODERIN®: https://cima.aemps.es/cima/pdfs/es/ft/53202/53202_ft.pdf
- Tacrolimus: ficha técnica de referencia ADVAGRAF→: https://www.ema.europa.eu/en/documents/product-information/advagraf-epar-product-information_es.pdf
- Tacrolimus: ficha técnica de referencia MODIGRAF→: https://www.ema.europa.eu/en/documents/product-information/modigraf-epar-product-information_es.pdf

13.1.7. VALORACIÓN DE LOS ACONTECIMIENTOS ADVERSOS

El investigador hará el seguimiento y recogerá de manera sistemática los AA desde la primera administración del medicamento en investigación hasta la visita final de seguimiento del sujeto.

Los AA se registrarán en la historia clínica del paciente dejando constancia de la relación causal con el tratamiento del ensayo. **Se recogerán en el CRD-e los AA cuando se considere que existe una posible relación de causalidad con el medicamento** en investigación, cuando **sean graves**.

Asimismo, el investigador evaluará y registrará con detalle de los AA lo siguiente:

Descripción del acontecimiento.

Fechas de inicio y final.

Gravedad.

Duración: si ha persistido por un tiempo determinado (días, horas o minutos) o continuo (si todavía está presente al final del ensayo clínico).

Medidas tomadas como:

- Ninguna: No se aplica ninguna medida.
- Medicación: Cualquier medicación iniciada para remediar el acontecimiento adverso, y/o si se realizan cambios en la dosis o vía de administración de la medicación que estuviera tomando el paciente.
- Interrupción del estudio: Cuando es necesario interrumpir la participación del paciente en el estudio debido a un AA.
- Otras: Cuando las medidas tomadas son distintas a la administración de un medicamento. Por ejemplo, fisioterapia, una intervención quirúrgica, etc.

Medidas tomadas frente a los tratamientos del estudio:

- Ninguna
- Disminución de la dosis
- Aumento de la dosis
- Interrupción temporal de la medicación
- Retirada de la medicación

Causalidad con el Medicamento en investigación.

- AA relacionado
- AA no relacionado

Desenlace:

- Recuperado
- En recuperación
- No recuperado
- Recuperado con secuelas
- Mortal.

13.2 SEGUIMIENTO DE LOS PACIENTES CON ACONTECIMIENTOS ADVERSOS

El investigador hará el seguimiento y recogerá de manera sistemática los AA desde la primera administración del medicamento en investigación hasta la visita final de seguimiento del sujeto.

A los sujetos que presenten AA, o cualquier resultado de laboratorio anormal que se considere clínicamente relevante, se les realizará el seguimiento pertinente hasta llegar a una resolución satisfactoria, hasta que se estabilice, o hasta que pueda ser explicado por otras causas y el juicio clínico indique que no son necesarias más evaluaciones.

13.3. NOTIFICACIÓN DE LOS ACONTECIMIENTOS ADVERSOS

Todos los AAGs (incluida la muerte), independientemente de su relación con los medicamentos en Investigación, deberán ser notificados lo antes posible, y **nunca más tarde de las 24 horas siguientes al conocimiento de la presentación del evento, a la persona o departamento responsable de la farmacovigilancia**

El Investigador realizará la notificación mediante el *Formulario de Notificación de AAG* enviándolo al correo electrónico del promotor o de quien asuma las tareas delegadas por el promotor.

El promotor o quien asuma las tareas de farmacovigilancia delegadas por el promotor revisará el formulario recibido y, si procede, solicitará información adicional al investigador.

El investigador proporcionará información al promotor o a quien asuma las tareas delegadas por el promotor siempre que se solicite y, en cualquier caso, cuando cambie su evaluación inicial en cuanto a gravedad o relación de causalidad. Asimismo, toda la información adicional referente al AA hasta la finalización del estudio o hasta su desenlace definitivo deberá ser comunicada sin demora, por medio de informes de seguimiento siguiendo el procedimiento de notificación descrito previamente.

Tanto el primer informe como los informes de seguimiento identificarán a los pacientes utilizando el código único de paciente y nunca por su nombre u otros datos identificables. En el informe también se incluirá el código del estudio.

El Investigador está obligado a cumplir con los requisitos legales que sean de aplicación con respecto a la comunicación de AAG.

El promotor o quien asuma las tareas delegadas por el promotor llevará un registro detallado de todos los AAG y AA de especial interés que le sean comunicados por los investigadores.

13.4. REACCIONES ADVERSAS GRAVES E INESPERADAS

El promotor o quien asuma las tareas delegadas por el promotor notificará todas las sospechas de RAGI de acuerdo con la normativa vigente sobre ensayos clínicos a la AEMPS en un plazo máximo de 15 días naturales a partir del momento en el que se tenga conocimiento de las mismas. Se notificarán al órgano competente de cada una de las CCAA donde se realiza el ensayo (si corresponde), las sospechas de RAGI ocurridas en los centros sanitarios de su Comunidad.

Cuando la RAGI haya ocasionado la muerte del paciente o puesto en peligro su vida, la notificación se realizará en un plazo máximo de 7 días naturales a partir del momento en el que se tenga conocimiento de la misma. Se complementará la información pertinente relativa a los hechos posteriores en un plazo de ocho días.

13.5. ACONTECIMIENTOS ADVERSAS DE ESPECIAL INTERÉS

El investigador registrará en el eCDR y comunicará al promotor o a quien asuma las tareas delegadas por el promotor, los AA que se consideran de especial interés tan pronto como sea posible y no más tarde de 15 días después de que tenga conocimiento de ellos.

Los AA que se consideran de especial interés son:

- Hipertensión refractaria (definida como la aparición de mal control de la tensión arterial a pesar de 3 fármacos antihipetensivos incluyendo un diurético)
- Insuficiencia renal (descenso superior al 50% del FG respecto al basal)
- Taquicardia ventricular

13.6. APERTURA DEL CIEGO Y DESENMASCARAMIENTO

No aplica.

13.7. NOTIFICACIÓN EXPEDITIVA DE OTRA INFORMACION DE SEGURIDAD RELEVANTE

El promotor o quien asuma las tareas delegadas por el promotor notificaran tan pronto como sea posible y no más tarde de 15 días después de que tenga conocimiento de ella cualquier información que pudiera suponer una modificación de la relación riesgo/beneficio del medicamento en Investigación o que tuviese relevancia suficiente como para recomendar modificaciones en la administración del medicamento en Investigación o en la realización del estudio.

13.8. EMBARAZO

En caso de producirse un embarazo durante el estudio, se ofrecerá la asistencia médica adecuada a la paciente. En este caso, se solicitará el consentimiento de la recogida de datos del mismo y de los datos de salud del bebé hasta el momento del nacimiento, con el objetivo de realizar el seguimiento del estado de la madre y el bebé y garantizando el cumplimiento de la nueva Ley Orgánica de Protección de Datos Personales y Garantía de los Derechos Digitales 3/2018 del 5 de Diciembre.

El investigador notificará la gestación al promotor o a quien asuma las tareas delegadas por el promotor en el plazo de 24 horas desde su conocimiento y se retirará a la paciente del estudio. Si el desenlace del embarazo cumple criterios de AAG o si el recién nacido presenta un acontecimiento grave se seguirán los procedimientos descritos para la notificación de AAG.

La notificación se realizará utilizando el formulario específico de notificación de embarazo, el cual se enviará por FAX o correo electrónico al mismo contacto que recibirá las notificaciones de AAG.

13.9. INFORME A LOS INVESTIGADORES

El promotor presentará a los investigadores la información de seguridad que podría impactar en la seguridad de los pacientes incluidos en el estudio tan pronto como sea posible. El investigador será informado de las modificaciones del protocolo debidas a motivos de seguridad.

14. CONSIDERACIONES ESTADÍSTICAS

14.1 DETERMINACIÓN DEL TAMAÑO DE LA MUESTRA

El tiempo mediano hasta la estabilidad clínica en grupo control se espera que sea de 16 días. Si el hazard ratio de estabilidad clínica de los pacientes de control en relación a los pacientes del grupo experimental es 0,52, habrá que incluir 42 pacientes en cada grupo para poder rechazar la hipótesis nula de igualdad con una potencia del 80%. La probabilidad de error Tipo I asociada con esta prueba de hipótesis es 0,05, y en cálculo incluye un 5% de pérdidas.

14.2. GRUPOS DE ANÁLISIS

Todos los datos recogidos para el presente estudio se incluirán en listados detallados hasta el nivel individual (por paciente). Se realizarán análisis descriptivos e inferenciales basados en los siguientes grupos de análisis:

- ITT: La población del análisis principal será por intención de tratamiento (ITT).
- En el caso que, durante el periodo de tiempo que requiera este ensayo clínico, aparecieran nuevos criterios para evaluar a un respondedor, en la reunión para preparar el plan de análisis estadístico (antes de analizar los datos), se podrá definir una ITT-2 de acuerdo con los nuevos criterios.
- También se llevará a cabo un análisis en la población por protocolo (PP) como análisis de sensibilidad.

14.2.1. GRUPO DE ANÁLISIS DE SEGURIDAD

El grupo de análisis seguridad incluye todos los sujetos incluidos en el estudio que hayan recibido al menos una dosis de tratamiento experimental. En principio, se prevé que coincida con la mitad de pacientes incluidos en el estudio. En este grupo se realizarán los análisis de seguridad.

14.2.2. GRUPO DE ANÁLISIS POR INTENCIÓN DE TRATAR (ITT)

El grupo de análisis por intención de tratar (ITT) incluye todos los sujetos del grupo de seguridad en quienes además se disponga de una evaluación de eficacia a la semana de haber iniciado el tratamiento experimental en estudio o a lo largo del estudio. En este grupo se realizarán los análisis de eficacia.

14.2.3. GRUPO DE ANÁLISIS POR PROTOCOLO (PP)

La población del análisis principal será por protocolo (PP).

En caso de detectarse que una proporción relevante de sujetos (>10%) presentara desviaciones relevantes del protocolo (por ejemplo, incumplimiento de los criterios de selección, incumplimiento o imposibilidad de recibir el tratamiento en estudio), se definiría el grupo de análisis por protocolo del que se excluiría a los sujetos con dichas desviaciones. En caso de definirse este grupo, se repetirían todos los análisis exploratorios de eficacia para él a modo de prueba de sensibilidad.

Las desviaciones del protocolo se consensuarán antes del inicio de los análisis de los datos (en la reunión de “Plan de Análisis Estadístico”). En ese momento también se decidirá si se define o no el grupo de análisis por protocolo.

14.3. MÉTODOS ESTADÍSTICOS

El estadístico que finalmente realizará los análisis será ciego para el tratamiento recibido por los pacientes (régimen corto vs régimen largo de tratamiento 'antiviral'). Se utilizará R versión 3.6.2 o superior para Windows (R Foundation for Statistical Computing, http://www.r-project.org) para el tratamiento y análisis de datos

14.3.1. DATOS DEMOGRÁFICOS Y BASALES

Todas las variables recogidas se resumirán en tablas mediante los estadísticos descriptivos adecuados (medidas de tendencia central y dispersión, y frecuencias absolutas y relativas, según proceda). Los datos basales se describirán para toda la muestra, por separado en cada grupo de tratamiento.

14.3.2. RETIRADAS Y ABANDONOS

Para la imputación de los datos ausentes se seguirán dos principios (European Medicines Agency 1998): que el estimador de los efectos del tratamiento no esté sesgado y que se evite el aumento del error de tipo I. Debido a que la historia natural de la enfermedad objeto de estudio es hacia el empeoramiento, el empleo de técnicas de imputación que respeten estos dos principios puede ser complicado. Sin embargo, puesto que el contraste de hipótesis descrito anteriormente garantiza la sensibilidad interna del estudio al esperarse mejoría en el grupo en tratamiento de régimen corto de tratamiento antiviral, la técnica de arrastre de la última observación disponible (LOCF del inglés *Last Observation Carried Forward*) constituye una aproximación conservadora.

14.3.2.1. ANÁLISIS PRINCIPAL DE EFICACIA

El análisis principal se realizará cuando todos los pacientes hayan alcanzado la estabilidad clínica, fracaso clínico o bien, lleven 56 días ingresados ante lo cual serán considerados fracasos también. La comparación entre el tiempo hasta la estabilidad clínica entre los grupos de estudio se realizará mediante el test de log-rank. Para cuantificar la magnitud de la asociación se estimará el hazard ratio con un modelo de riesgos proporcionales de Cox y se acompañará de los intervalos de confianza al 95%. La variable principal se analizará además en la población por protocolo.

La variable principal se analizará además en la población por intención de tratar. Como análisis adicional, se construirá un modelo de riesgos proporcionales de Cox ajustado por factores de confusión clinicalmente relevantes como la edad, sexo, comorbilidades mediante el índice de Charlson, índices para valorar la gravedad de la neumonía (PSI y CURB-65), índices de disfunción orgánica (SOFA) y parámetros inflamatorios. Además, está planificado un análisis de subgrupos, para investigar si el efecto del tratamiento varía en función de la gravedad de COVID-19, en los pacientes con un mayor grado de comorbilidades y por grupos etarios.

Además, tras discutirlo con el equipo investigador, están planificados los siguientes análisis de subgrupos como análisis de sensibilidad, para investigar si el efecto del tratamiento varía entre los pacientes con y sin necesidad de ventilación mecánica invasiva, entre los diferentes grados de gravedad de la neumonía por SARS-CoV-2, entre los diferentes grados de gravedad de la SOFA score, entre los distintos patrones radiológicos basales (alveolar, intersticial), y entre los distintos valores de parámetros inflamatorios basales.

14.3.2.2. ANÁLISIS SECUNDARIOS DE EFICACIA

Para la evaluación de las variables secundarias calcularemos las estimaciones no ajustadas y ajustadas del tamaño del efecto y los correspondientes intervalos de confianza del 95% utilizando regresión lineal, logística o de riesgos proporcionales de Cox.

14.3.3. Seguridad

Los análisis de seguridad se llevarán a cabo en el grupo de análisis de seguridad. Se codificarán los acontecimientos adversos registrados durante el estudio según la última versión disponible del diccionario MedDRA y se describirán mediante sus frecuencias absolutas y relativas por grupo de estudio, de acuerdo con la gravedad y la relación con el tratamiento.

14.3.4. Análisis intermedios

A la mitad del reclutamiento, 21 pacientes por brazo, se realizará un análisis intermedio de eficacia y seguridad. Para ello se aplicará una corrección del error de tipo I siguiendo Lan–DeMets (O'Brien–Fleming) en la evaluación de la eficacia.

15. REFERENCIAS BIBLIOGRÁFICAS

1.- Lai CC, Shih TP, Ko WC, Tang HJ, Hsueh PR. Severe acute respiratory syndrome coronavirus 2 (SARS-CoV-2) and coronavirus disease-2019 (COVID-19): The epidemic and the challenges. Int J Antimicrob Agents. 2020 Feb 17:105924. doi: 10.1016/j.ijantimicag.2020.105924. [Epub ahead of print]

2.- Wang LS, Wang YR, Ye DW, Liu QQ. A review of the 2019 Novel Coronavirus (COVID-19) based on current evidence". Int J Antimicrob Agents. 2020 [Epub ahead of print]

3.- WHO Director-General's opening remarks at the media briefing on COVID-19 - 11 March 2020. [https://www.who.int/dg/speeches/detail/who-director-general-s-opening-remarks-atthe-media-briefing-on-covid-19---11-march-2020]

4.- Wu Z, McGoogan JM. Characteristics of and important lessons from the coronavirus disease 2019 (COVID-19) outbreak in China: summary of a report of 72 314 cases from the Chinese Center for Disease Control and Prevention. JAMA. 2020 Feb 24. doi: 10.1001/jama.2020.2648. [Epub ahead of print]

5.- Kalil AC. Treating COVID-19-Off-Label Drug Use, Compassionate Use, and Randomized Clinical Trials During Pandemics. JAMA. 2020 Mar 24. doi: 10.1001/jama.2020.4742.

6.- Colson P, Rolain JM, Raoult D. Chloroquine for the 2019 novel coronavirus SARS-CoV2. Int J Antimicrob Agents. 2020 Feb 15:105923. doi: 10.1016/j.ijantimicag.2020.105923. [Epub ahead of print]

7.- Colson P, Rolain JM, Lagier JC, Brouqui P, Raoult D. Chloroquine and hydroxychloroquine as available weapons to fight COVID-19. Int J Antimicrob Agents. 2020 [Epub ahead of print]

8.- Wang M, Cao R, Zhang L, Yang X, Liu J, Xu M, et al. Remdesivir and chloroquine effectively inhibit the recently emerged novel coronavirus (2019-nCoV) in vitro. Cell Res. 2020;10-0282.

9.- Gao J, Tian Z, Yang X. Breakthrough: Chloroquine phosphate has shown apparent efficacy in treatment of COVID-19 associated pneumonia in clinical studies. Biosci Trends. 2020 Feb 19. doi: 10.5582/bst.2020.01047. [Epub ahead of print]

10.- Multicenter collaboration group of Department of Science and Technology of Guangdong Province and Health Commission of Guangdong Province for chloroquine in the treatment of novel coronavirus pneumonia. Expert consensus on chloroquine phosphate for the treatment of novel coronavirus pneumonia]. Zhonghua Jie He He Hu Xi Za Zhi. 2020 Mar 12;43(3):185-188. doi: 10.3760/cma.j.issn.1001-0939.2020.03.009.

11.- Biot C, Daher W, Chavain N, Fandeur T, Khalife J, Dive D, et al. Design and synthesis of hydroxyferroquine derivatives with antimalarial and antiviral activities. J Med Chem. 2006;49:2845-2849.

12.- Gautret P, Lagiera JC, Parolaa P, Hoanga VT, Meddeba L, Mailhea M, *et al*. Hydroxychloroquine and azithromycin as a treatment of COVID-19: results of an open-label non-randomized clinical trial. International Journal of Antimicrobial Agents. 2020: In Press 17 March 2020 – DOI : 10.1016/j.ijantimicag.2020.105949.

13.- Marmor MF, Kellner U, Lai TY, Melles RB, Mieler WF; American Academy ofOphthalmology. Recommendations on Screening for Chloroquine and Hydroxychloroquine Retinopathy. (2016 Revision). Ophthalmology. 2016 Jun;123(6):1386-94. doi:10.1016/j.ophtha.2016.01.058. Epub 2016 Mar 16.

14.- Yao X, Ye F, Zhang M, Cui C, Huang B, Niu P, et al. In Vitro Antiviral Activity and Projection of Optimized Dosing Design of Hydroxychloroquine for the Treatment of Severe Acute Respiratory Syndrome Coronavirus 2 (SARS-CoV-2). Clin Infect Dis. 2020 Mar 9. pii:ciaa237. doi: 10.1093/cid/ciaa237. [Epub ahead of print]

15.- Cao B, Wang Y, Wen D, Liu W, Wang Jingli, Fan G, *et al*. A Trial of Lopinavir–Ritonavir in Adults Hospitalized with Severe Covid-19. NEJM. 2020: published March 18th.

16.- Wang D, Hu B, Hu C, Zhu F, Liu X, Zhang J, Wang B, Xiang H, Cheng Z, Xiong Y, Zhao Y, Li Y, Wang X, Peng Z. Clinical Characteristics of 138 Hospitalized Patients With 2019 Novel Coronavirus-Infected Pneumonia in Wuhan, China. JAMA. 2020 Feb 7.

17.- Wu C, Chen X, Cai Y, Xia J, Zhou X, Xu S, Huang H, Zhang L, Zhou X, Du C, Zhang Y, Song J, Wang S, Chao Y, Yang Z, Xu J, Zhou X, Chen D, Xiong W, Xu L, Zhou F, Jiang J, Bai C, Zheng J, Song Y. Risk Factors Associated With Acute Respiratory Distress Syndrome and Death in Patients With Coronavirus Disease 2019 Pneumonia in Wuhan, China. JAMA Intern Med. 2020 Mar 13.

18.- Ruan Q, Yang K, Wang W, Jiang L, Song J. Clinical predictors of mortality due to COVID-19 based on an analysis of data of 150 patients from Wuhan, China.Intensive Care Med. 2020 Mar 3.

19.- Russell B, Moss C, George G, Santaolalla A, Cope A, Papa S, Van Hemelrijck M. Associations between immune-suppressive and stimulating drugs and novel COVID-19-a systematic review of current evidence. Ecancermedicalscience. 2020 Mar 27;14:1022. doi: 10.3332/ecancer.2020.1022.

20.- Zhou F, Yu T, Du R, Fan G, Liu Y, Liu Z, Xiang J, Wang Y, Song B, Gu X, Guan L, Wei Y, Li H, Wu X, Xu J, Tu S, Zhang Y, Chen H, Cao B. Clinical course and risk factors for mortality of adult inpatients with COVID-19 in Wuhan, China: a retrospective cohort study. Lancet. 2020 Mar 11. pii: S0140-6736(20)30566-3.

21.- Arabi YM, Mandourah Y, Al-Hameed F, et al; Saudi Critical Care Trial Group. Corticosteroid therapy for critically ill patients with Middle East respiratory syndrome. Am J Respir Crit Care Med. 2018;197(6):757-767. doi:10.1164/rccm.201706-1172OC

22.- Lee N, Allen Chan KC, Hui DS, et al. Effects of early corticosteroid treatment on plasma SARS-associated coronavirus RNA concentrations in adult patients.J Clin Virol. 2004;31(4):304-309. doi:10.1016/j.jcv.2004.07.006

23.- Stockman LJ, Bellamy R, Garner P. SARS: systematic review of treatment effects. PLoS Med. 2006;3(9):e343. Epub 2006/09/14. doi: 10.1371/journal.pmed.0030343. PubMed PMID: 16968120; PMCID: PMC1564166.

24.- Rodrigo C, Leonardi-Bee J, Nguyen-Van-Tam J, Lim WS. Corticosteroids as adjunctive therapy in the treatment of influenza. Cochrane Database Syst Rev. 2016;3:CD010406. Epub 2016/03/08. doi: 10.1002/14651858.CD010406.pub2. PubMed PMID: 26950335.

25.- Russell CD, Millar JE, Baillie JK. Clinical evidence does not support corticosteroid treatment for 2019-nCoV lung injury. Lancet. 2020;395(10223):473-475. doi:10.1016/S0140-6736(20)30317-2.

26.- Hirano K, Ichikawa T, Nakao K, Matsumoto A, Miyaaki H, Shibata H, Eguchi S, Takatsuki M, Ikeda M, Yamasaki H, Kato N, Kanematsu T, Ishii N, Eguchi K. Differential effects of calcineurin inhibitors, tacrolimus and cyclosporin a, on interferon-induced antiviral protein in human hepatocyte cells. Liver Transpl. 2008 Mar;14(3):292-8.

27.- Mehta P, McAuley DF, Brown M, Sanchez E, Tattersall RS, Manson JJ; HLH Across Speciality Collaboration, UK. COVID-19: consider cytokine storm syndromes and immunosuppression. Lancet. 2020 Mar 16.

28.- Ramos-Casals M, Brito-Zerón P, López-Guillermo A, Khamashta MA, Bosch X. Adult haemophagocytic syndrome. Lancet. 2014 Apr 26;383(9927):1503-1516.

29.- Carbajo-Lozoya J, Müller MA, and Kallies S, et al (2012) Replication of human coronaviruses SARS-CoV, HCoV-NL63 and HCoV-229E is inhibited by the drug FK506 Virus Res 165(1) 112–117 https://doi.org/10.1016/j.virusres.2012.02.002 PMID: 22349148

30.- Carbajo-Lozoya J, Ma-Lauer Y, and Malesevic M, et al Human coronavirus NL63 replication is cyclophilin A-dependent and inhibited by non-immunosuppressive cyclosporine A-derivatives including Alisporivir Virus Res 184 44–53 PMID: 24566223

31.- AlGhamdi M, Mushtaq F, and Awn N, et al (2015) MERS CoV infection in two renal transplant recipients: case report Am J Transplant 15(4) 1101–1104. ttps://doi.org/10.1111/ajt.13085 PMID: 25716741

16. APÉNDICES

16.1 LISTADO DE INVESTIGADORES PRINCIPALES DE LOS CENTROS:

Centro de realización del estudio	Investigador principal
Hospital Universitario de Bellvitge	Dr. Xavier Solanich Moreno<br>email: xsolanich@bellvitgehospital.cat

16.2 APÉNDICE 2: FICHA TÉCNICA

- Metilprednisolona: ficha técnica de referencia URBASON®: https://cima.aemps.es/cima/pdfs/es/ft/34023/34023_ft.pdf
- Metilprednisolona: ficha técnica de referencia SOLU-MODERIN®: https://cima.aemps.es/cima/pdfs/es/ft/53202/53202_ft.pdf
- Tacrolimus: ficha técnica de referencia ADVAGRAF→: https://www.ema.europa.eu/en/documents/product-information/advagraf-epar-product-information_es.pdf
- Tacrolimus: ficha técnica de referencia MODIGRAF→: https://www.ema.europa.eu/en/documents/product-information/modigraf-epar-product-information_es.pdf

16.3 Apéndice 3: HOJA INFORMACIÓN PACIENTE

Proyecto de investigación titulado: ***Ensayo clínico de Fase II, pragmático, con asignación aleatoria, controlado, abierto y unicéntrico para evaluar pulsos de metilprednisolona y tacrolimus en pacientes hospitalizados con neumonía grave secundaria a COVID-19 (TACROVID)***

EudraCT:

Investigador principal: Dr. Xavier Solanich Moreno

Servicio de Medicina Interna del Hospital Universitario de Bellvitge.

Nos dirigimos a usted para informarle sobre un estudio de investigación en el que se le invita a participar. El estudio ha sido aprobado por un Comité de Ética de la Investigación con medicamentos y por la Agencia Española de Medicamentos y Productos Sanitarios, de acuerdo a la legislación vigente, el Real Decreto 1090/2015 de 4 de diciembre y el Reglamento Europeo 536/2014 de 16 de abril, por los que se regulan los ensayos clínicos con medicamentos.

Nuestra intención es que usted reciba la información correcta y suficiente para que pueda decidir si acepta o no participar en este estudio. Para ello lea esta hoja informativa con atención y nosotros le aclararemos las dudas que le puedan surgir. Además, puede consultar con las personas que considere oportuno.

Debe saber que su participación en este estudio es voluntaria y que puede decidir NO participar. Si decide participar, puede cambiar su decisión y retirar el consentimiento en cualquier momento, sin que por ello se altere la relación con su médico ni se produzca perjuicio alguno en su atención sanitaria.

Introducción: El tratamiento de la enfermedad por coronavirus se basa en las terapias de soporte vital. No existen aún buenos estudios que nos permitan administrar medicamentos con eficacia ni seguridad probada para tratar esta enfermedad. La urgencia asistencial hace que, a pesar de la falta de evidencia, se utilicen ya algunos fármacos en la práctica diaria. Fundamentalmente, se están realizando ensayos para detener la multiplicación del virus mediante tratamientos antivirales.

La principal causa de muerte es la falta de oxígeno debía a la inflamación pulmonar desencadenada por la infección viral. Se están estudiando fármacos para bloquear la inflamación en los casos de mayor gravedad. Tacrolimus es un fármaco oral que bloquea varios componentes del sistema inmune. Junto con 3 dosis puntuales de corticoides endovenosos podría ser beneficioso para mejorar la inflamación que produce el coronavirus a nivel pulmonar

<u>Beneficios:</u> Su participación en este estudio contribuiría con datos para aumentar el conocimiento sobre la eficacia y seguridad de corticoides con tacrolimus en la afectación pulmonar por coronavirus, así como sobre los posibles predictores de respuesta.

<u>Procedimientos del estudio</u>: Se prevé incluir 84 participantes en total en este estudio, 42 pacientes en tratamiento convencional y otros 42 pacientes con el tratamiento convencional junto con los corticoides y tacrolimus. Así pues, siempre va a recibir el tratamiento convencional y en función del grupo al que sea asignado también recibirá el tratamiento que se está evaluando. El procedimiento de asignación a uno de los dos grupos de tratamiento se realizará al azar. Si decide participar, se procederá a una primera visita para verificar que puede cumplir con los criterios para participar en el estudio. Una vez esté incluida en el estudio se le asignará uno de los dos tipos de tratamiento.

Independientemente del tratamiento que usted realice se recogerán datos demográficos y sobre la medicación que toma. Se controlaran las constantes vitales diariamente. Además se realizará una analítica cada 2 días mientas esté ingresado.

Durante las visitas, se le solicitará notificar cualquier evento adverso que le suceda o cambios en medicación. El tratamiento del estudio finalizará tras 3 días de estabilidad clínica.

<u>Los riesgos, molestias y efectos secundarios posibles</u>,

Respecto al tratamiento con corticoides son mínimos ya que solo los va a recibir durante 3 días consecutivos. De todos modos, se podría asociar a elevaciones transitorias del azúcar en sangre o la tensión arterial. Además podría notar cierto nerviosismo en algunos momentos. Sin embargo no presenta contraindicaciones en el caso de insuficiencia renal y/o hepática, ni interacción con la toma de otros fármacos

Respecto al tacrolimus podría notar sensación de nauseas o vómitos transitorios. Un exceso de este medicamento puede producir empeoramiento de la función renal o arritmias. Para evitar que se produzcan complicaciones médicos realizaran un electrocardiograma (estudio del ritmo cardíaco) cada 48 horas y además controlaran sus niveles en sangre también cada 48 horas para evitar que se acumule este fármaco en exceso.

Los dos fármacos están aprobados por las autoridades sanitarias competentes, existe información al acceso de todo el mundo sobre los efectos secundarios de la metilprednisolona y el tacrolimus. Por favor, hable con el médico de su estudio para obtener una lista completa de los efectos secundarios comunicados con este fármaco y en cualquier caso se le entregará el prospecto del fármaco.

Cualquier nueva información referente a los fármacos utilizados en el estudio y que pueda afectar a su decisión para continuar en el estudio, le será comunicada por el médico lo antes posible y, si es necesario, se firmará un nuevo consentimiento.

Si usted decide participar, se propone realizar un seguimiento minucioso de cualquier efecto adverso descrito por el medicamento para garantizar su seguridad.

Este estudio se ajusta a la legislación vigente (Real decreto 1090/2015), considerándose de bajo nivel de intervención, por lo que queda amparado con póliza de seguros del hospital en caso de menoscabo de su salud o de lesiones que pudieran producirse en relación con su participación en el estudio, siempre que no sean consecuencia de la propia enfermedad que se estudia o de la evolución propia de su enfermedad de base. Si desea más información relativa a este apartado, consulte con el investigador principal de este estudio.

Le informamos que es posible que su participación en este ensayo clínico pueda modificar las condiciones generales y particulares (cobertura) de sus pólizas de seguros (vida, salud, accidente...). Por ello, le recomendamos que se ponga en contacto con su aseguradora para determinar si la participación en este estudio afectará a su actual póliza de seguros.

El promotor del estudio es el responsable de gestionar la financiación del mismo. Usted no tendrá que pagar por los medicamentos ni por pruebas específicas del estudio. Su participación en el estudio no le supondrá ningún gasto adicional a la práctica clínica habitual.

<u>Otra información relevante:</u>

Debe saber que puede ser excluido del estudio si el promotor o los investigadores del estudio lo consideran oportuno, ya sea por motivos de seguridad, por cualquier acontecimiento adverso que se produzca por la medicación en estudio o porque consideren que no está cumpliendo con los procedimientos establecidos. En cualquiera de los casos, usted recibirá una explicación adecuada del motivo que ha ocasionado su retirada del estudio.

En caso de finalización anticipada del ensayo por parte del promotor, se prevé informar oportunamente a los participantes acerca de los motivos.

En el presente consentimiento también podrá hacer constar si desea que los remanentes de las muestras de este estudio se conserven en el Biobanco HUB-ICO-IDIBELL, para poder utilizarlos en otros estudios de investigación, de acuerdo con la normativa vigente.

<u>Protección de datos personales:</u>

Tanto el Promotor como el centro son responsables respectivamente del tratamiento de sus datos y se comprometen al cumplimiento del Reglamento (UE) 2016/679 del Parlamento europeo y del Consejo de 27 de abril de 2016 de Protección de Datos (RGPD), así como al resto de leyes y normativa vigente y aplicable (Ley Orgánica de Protección de Datos Personales y Garantia de los Derechos Digitales 3/2018 del 05 de Diciembre).

Tanto el Centro como el Promotor son responsables respectivamente del tratamiento de sus datos y se comprometen a cumplir con la normativa de protección de datos en vigor. Los datos recogidos para el estudio estarán identificados mediante un código, de manera que no incluya información que pueda identificarle (ni nombre ni apellidos, ni iniciales ni dirección, nº seguridad social, etc), y sólo su médico del estudio/colaboradores podrá relacionar dichos datos con usted y con su historia clínica. Por lo tanto, su identidad no será revelada a ninguna otra persona salvo a las autoridades sanitarias, cuando así lo requieran o en casos de urgencia médica.

Los Comités de Ética de la Investigación, los representantes de la Autoridad Sanitaria en materia de inspección (Agencia Española de Medicamentos y Productos Sanitarios, autoridades sanitarias extranjeras) y el personal autorizado por el Promotor (monitores, auditores), únicamente podrán acceder para comprobar los datos personales, los procedimientos del estudio clínico y el cumplimiento de las normas de buena práctica clínica (siempre manteniendo la confidencialidad de la información).

Los datos se recogerán en un fichero de investigación, responsabilidad de la institución y se tratarán en el marco de su participación en este estudio. Los datos del presente estudio podrán utilizarse para futuras investigaciones. El promotor adoptará las medidas pertinentes para garantizar la protección de su privacidad y no permitirá que sus datos se crucen con otras bases de datos que pudieran permitir su identificación.

De acuerdo a lo que establece la legislación de protección de datos, usted puede ejercer los derechos de acceso, modificación, oposición y cancelación de datos, para lo cual deberá dirigirse a su médico del estudio. Además también puede limitar el tratamiento de datos que sean incorrectos, solicitar una copia o que se trasladen a un tercero (portabilidad) los datos que usted ha facilitado para el estudio. Para ejercitar sus derechos, diríjase al investigador principal del estudio o al Delegado de protección de datos de la institución (ICS), email: dataprotection@idibell.cat.

Si usted decide retirar el consentimiento para participar en este estudio, los datos que ya se hayan recogido hasta el momento no se podrán eliminar para garantizar la validez de la investigación y cumplir con los deberes legales y los requisitos de autorización de medicamentos. Además, en este estudio se consultaría su historia clínica electrónica para comprobar datos de seguimiento de seguridad al mes de su alta médica. Así mismo tiene derecho a dirigirse a la Agencia de Protección de Datos si no quedara satisfecho.

El Investigador y el Promotor están obligados a conservar los datos recogidos para el estudio al menos hasta 25 años tras su finalización. Posteriormente, su información personal solo se conservará por el centro para el cuidado de su salud y por el promotor para otros fines de investigación científica si usted hubiera otorgado su consentimiento para ello y si así lo permite la ley y requisitos éticos aplicables.

Si se realizara una transferencia de sus datos codificados fuera de la UE a las entidades de nuestro grupo, a prestadores de servicios o a investigadores científicos que colaboren con nosotros, los datos del participante quedarán protegidos con salvaguardas tales como contratos u otros mecanismos por las autoridades de protección de datos. Si el participante quiere saber más al respecto, puede contactar al/ a la Delegado de Protección de Datos del Promotor: Laura Villagrasa, dataprotection@idibell.cat.

Si necesita más información sobre este estudio puede contactar con el **investigador responsable**, Dr./a. **Xavier Solanich Moreno**
Servicio de Medicina Interna del Hospital Universitario de Bellvitge, en la 7ª planta de edificio principal.
Número telefónico de contacto, *932602324*

¿QUÉ TRATAMIENTO RECIBIRÉ CUANDO FINALICE EL ENSAYO CLÍNICO?:
Cuando acabe su participación recibirá el mejor tratamiento disponible y que su médico considere el más adecuado para su enfermedad. Por lo tanto, ni el investigador ni el promotor adquieren compromiso alguno de modificar el tratamiento recibido durante ni fuera de este estudio.

HOJA DE CONSENTIMIENTO INFORMADO DEL PACIENTE

Título del estudio: "***Ensayo clínico de Fase II, pragmático, con asignación aleatoria, controlado, abierto y unicéntrico para evaluar pulsos de metilprednisolona y tacrolimus en pacientes hospitalizados con neumonía grave secundaria a COVID-19 (TACRO-BELL-COVID).***"

Código de Estudio: HUB-

Yo, (nombre y apellidos) ____. He leído la hoja de información que se me ha entregado.

He podido hacer preguntas sobre el estudio y he recibido información suficiente sobre el estudio.

Autorizo que los excedentes de las muestras para este estudio se conserven en el Biobanco HUB-ICO-IDIBELL para su uso en futuros proyectos de investigación ☐ SI ☐ NO

He hablado con (nombre y apellidos del investigador) ____

Así mismo, comprendo que mi participación es voluntaria y comprendo que puedo retirarme del estudio:

1. Cuando quiera.

2. Sin tener que dar explicaciones.

3. Sin que esto repercuta en mis cuidados médicos.

Recibiré una copia firmada y fechada de este documento de consentimiento informado.

Presto libremente mi conformidad para participar en el estudio.

____

Firma del participante Firma del investigador

Fecha: ____/____/____ Fecha: ____/____/____

HOJA DE CONSENTIMIENTO INFORMADO DEL REPRESENTANTE LEGAL

Título del estudio: "***Ensayo clínico de Fase II, pragmático, con asignación aleatoria, controlado, abierto y unicéntrico para evaluar pulsos de metilprednisolona y tacrolimus en pacientes hospitalizados con neumonía grave secundaria a COVID-19 (TACRO-BELL-COVID).***"

Código de Estudio: HUB-

Yo, (nombre y apellidos) ____ en calidad de ____________________________________ (Relación con el participante) de _______________________________________ (Nombre y apellidos del participante)

He leído la hoja de información que se me ha entregado.

He podido hacer preguntas sobre el estudio y he recibido información suficiente sobre el estudio.

Autorizo que los excedentes de las muestras para este estudio se conserven en el Biobanco HUB-ICO-IDIBELL para su uso en futuros proyectos de investigación ☐ SI ☐ NO

He hablado con (nombre y apellidos del investigador) _______________________________

Así mismo, comprendo que la participación del paciente es voluntaria y comprendo que puedo retirarme del estudio:

1. Cuando quiera.
2. Sin tener que dar explicaciones.
3. Sin que esto repercuta en mis cuidados médicos.

Recibiré una copia firmada y fechada de este documento de consentimiento informado.

Presto libremente mi conformidad para que el paciente participe en el estudio.

____

Firma del representante legal Firma del investigador

Fecha: ____/____/____ Fecha: ____/____/____
