# Supplementary material for: Methylprednisolone Pulses Plus Tacrolimus in Addition to Standard of Care vs. Standard of Care Alone in Patients With Severe COVID-19. A Randomized Controlled Trial
Source: Front Med (Lausanne). 2021 Jun 14;8:691712. doi: 10.3389/fmed.2021.691712 (PMC8236585; doi:10.3389/fmed.2021.691712)
Supplement: Supplementary file 3 [file Data_Sheet_3.PDF]

Supplementary Materials

Table of Contents

Supplemental Methods:

Laboratory tests...……………………..…………………………………………………2
Cytokine analysis……………………..……………………………………………… 2
Treatments used to treat COVID-19…......……………………………………………… 2
Additional Study Amendment Considerations……………………………………………. 2

Supplemental Tables and Figures

Figure S1. COVID-19-related mortality in the ITT population ……………………………... 4
Figure S2. Changes in cytokine profile at randomization, and at days 14, 28 and 56 in the ITT population……………………………………..5
Figure S3. Viral load by quantitative PCR and positive percentage in the nasopharyngeal and oropharyngeal specimens (A) and blood samples (B) in the ITT population ………………. 6

Table S1. Flow-chart of the trial showing the procedures that were conducted at each visit... 7
Table S2. Evolution of analytic parameters after days 5, 10 or hospital discharge (whichever was first), 28 and 56 in the ITT population…………...…………………...……………..…8
Table S3. Changes in cytokine profile at randomization, and at days 14, 28 and 56 in the ITT population ……………………………………………………………………………………9
Table S4. Chest X-ray at baseline and at day 56 in the ITT population …………………….. 11
Table S5. SARS-CoV-2 positive test and viral load by quantitative PCR in the upper respiratory tract and blood samples in the ITT population …………..……………………... 12
Table S6. Summary of adverse events and serious adverse events in the ITT population….. 13
Table S7. 8-point ordinal scale at 10, 28, and 58 days in the ITT population ………………. 15

<u>Supplemental Methods:</u>

Laboratory tests

Hematology (hemoglobin, hematocrit, platelet count, absolute lymphocyte count, leukocyte count with differential) assessed using Sysmex® XN2000 analyzer (Sysmex Europe GmbH; Norderstedt; Germany); Basic chemistry (creatinine, albumin, ALT, total bilirubin) and inflammatory parameters (ferritin, D-dimer, CRP, LDH) measured with Cobas® 6000/8000 analyzer (Roche Diagnostics®, Risch-Rotkreuz, Switzerland), which has spectrophotometry and immunochemistry modules with electrochemiluminescent detection; and coagulation (activated partial thromboplastin time, prothrombin time and fibrinogen) using ACLTOP® 550 analyzer (Werfen®; Barcelona, Spain). Tacrolimus levels were measured using a method based on ultra-high-performance liquid chromatography coupled with tandem mass spectrometry (HPLC-MS/MS)

Cytokine analysis

Serum was collected from all 55 patients included in the study and 27 healthy controls. Cytokine concentrations were measured using a custom bead-based Luminex multiplex immunoassay (R&D Systems, USA) that measures 16 proinflammatory cytokines (CXCL9/MIG, CXCL10/IP-10, G-CSF, IFN-alpha, IFN-beta, IFN-gamma, IL-1 beta, IL-1Ra, IL-2R alpha/CD25, IL-6, IL-8, IL-10, IL-18, MCP-1/CCL2, MCP-3/CCL7 and TNF-alpha). *The normal level for each cytokine was obtained by calculating the mean plus 2 standard deviations (SD) in a cohort of 27 healthy adult patients.

	Normal level* (pg/mL)		Normal level* (pg/mL)
**CXCL9/MIG**	<876.2	**IL-2R alpha/CD25**	<732.3
**CXCL10/IP-10**	<21.5	**IL-6**	<4.4
**G-CSF**	<54.6	**IL-8**	<28.3
**IFN-alpha**	<10.9	**IL-10**	<4.0
**IFN-beta**	<12.4	**IL-18**	<778.7
**IFN-gamma**	<44.2	**MCP-1/CCL2**	<499.2
**IL-1 beta**	<15.9	**MCP-3/CCL7**	<58.1
**IL-1Ra**	<954.8	**TNF-alpha**	<8.3

Treatments used to treat COVID-19

When the TACROVID trial was designed in March 2020, data from the main COVID-19 randomized controlled trials (RCT) were still not available. During the trial period, a large number of patients received off-label and compassionate therapies. They were administered according to Hospital Universitari de Bellvitge protocol as described below. Hydroxychloroquine (400mg twice daily the first day followed by 200 mg twice daily next 5-10 days), and lopinavir-ritonavir (400/100mg twice daily for 5-10 days) at hospital admission were used. Antibiotic therapy was added if bacterial co-infections were suspected. Corticosteroids as methylprednisolone IV 125 mg daily during three consecutive days, and tocilizumab IV as a single dose (600 mg if $\geq$ 75 kg or 400 mg if $<$ 75 kg) were allowed at the discretion of the treating physician when the $p\text{aO}_2/\text{FiO}_2$ value was $\leq$ 300 mmHg and there were at least one of the prognostic criteria for progression to acute respiratory distress syndrome (ARDS) [Wu C. JAMA Intern Med. 2020 Jul]. Some patients with COVID-19 progression despite the recommended immunosuppressive regimen received longer corticosteroids doses or anakinra in accordance with treating physicians. All patients admitted to the hospital received thromboprophylaxis with enoxaparin sodium 40 mg daily or bemiparin 3500 IU daily.

Additional Study Amendment Considerations

The protocol was approved by the EC and the AEMPS on March 31, 2020, in a process of authorization adapted to the pandemic situation and based on a protocol synopsis (March 28, 2020). A substantial amendment to the original protocol was submitted to the EC and the AEMPS in accordance with Spanish legislation, and accepted on April 9, 2020. In the experimental group we

decided to reduce tacrolimus plasma levels from 10–15 to 8–10 ng/mL, and not to maintain corticosteroids per protocol beyond the three pulses of methylprednisolone if their treating physicians did not consider it appropriate. In addition, some exclusion criteria were added, such as glomerular filtration ≤ 30 mL/min/1.73m2, leukopenia ≤ 4000 cells/µL or other conditions that cause immunosuppression, and concomitant and potentially serious infections. Another additional amendment was accepted by the EC and AEMPS on May 25, 2020, including a complete clinical study protocol with new secondary key outcomes (8-point ordinal scale and radiological abnormalities) that were analyzed in several COVID-19 clinical trials. These outcomes were being prospectively reported in the trial's eCRF, but were not reported in the protocol synopsis. We did not remove any of the previous key study outcomes. Finally, early termination was accepted on June 29, 2020. No new cases were introduced after May 2, 2020 due to the decrease in incidence after lockdown. Furthermore, according to the RECOVERY trial and another RCT, it was expected that patients would receive quite different treatment from those used in the first wave of the pandemic in Spain (e.g. dexamethasone 6 mg per day for 10 days instead of the three boluses of methylprednisolone, cessation of the use of lopinavir-ritonavir, hydroxychloroquine, azithromycin, etc.).

Figure S1. **COVID-19-related mortality in the ITT population.**

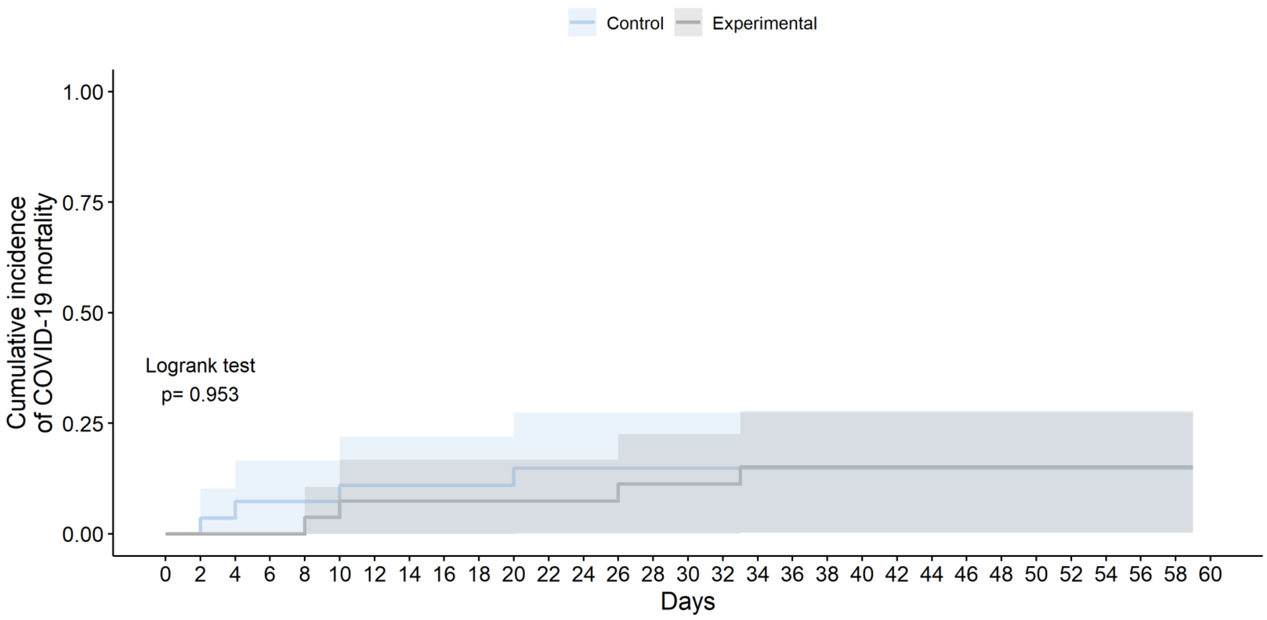

Figure S2. Changes in cytokine profile at randomization, and at days 14, 28 and 56 in the ITT population.

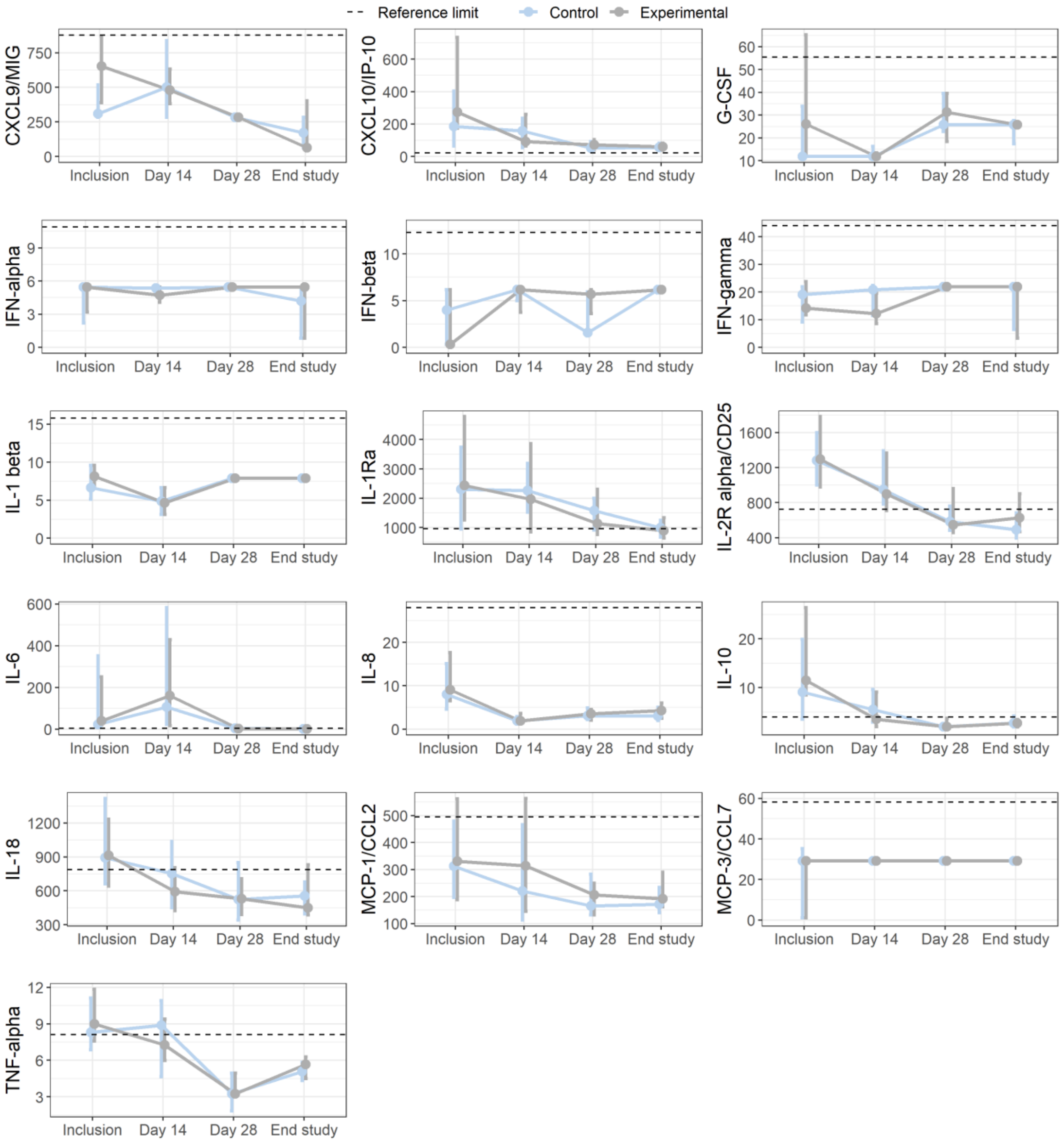

The median and the interquartile range of the expanded cytokine profile at inclusion, 14 days, 28 days, and at the end of the study. Normal level for each cytokine is shown by a horizontal dashed line. Units of measurement for each cytokine correspond to pg/ml.

Figure S3. Viral load by quantitative PCR and positive percentage in the nasopharyngeal and oropharyngeal specimens (A) and blood samples (B) in the ITT population.

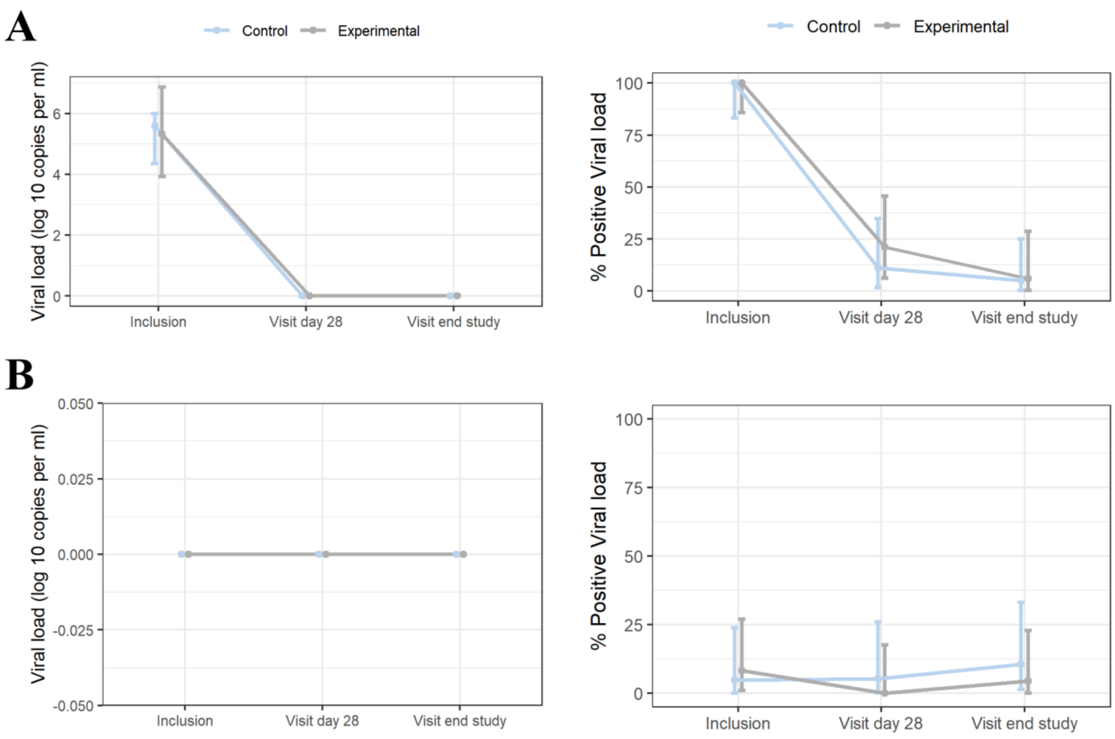

The median and interquartile range of the viral load at inclusion, 28 days, and end of the study by quantitative PCR and positive percentage in the nasopharyngeal and oropharyngeal specimens. Results less than the lower limit of quantification of the PCR assay and greater than the limit of qualitative detection are imputed with half or twofold of the limit, respectively; results of patients with viral-negative RNA are imputed with 0 $\log_{10}$ copies per mL.

Table S1. Flow-chart of the trial showing the procedures that were conducted at each visit

	Randomization visit (day 0)	Hospitalization visits (days n)	Visit 28 ± 3 days after the start of treatment	Visit 56 ± 3 days after the start of treatment
Inclusion / exclusion	x			
Informed consent	x			
Randomization	x			
Demographics/Comorbidities	x			
COVID-19 clinical data	x			
Vital signs	x	x	x	x
8-point ordinal scale	x	x	x	x
Laboratory (Hematology, Chemistry, Coagulation)	x	x	x	x
Tacrolimus blood levels		x		
Cytokine profile	x	x	x	x
Blood SARS-CoV-2 PCR	x	x	x	x
Nasopharyngeal SARS-CoV-2 PCR	x		x	x
Clinical stability	x	x	x	x
Lung tests (x-ray)	x	x[1]	x[1]	x
Study treatment	x	x	x	x
Concomitant medication	x	x	x	x
AE registration	x	x	x	x

(1) according to the attending physician. Abbreviations: PCR = polymerase chain reaction; AE = adverse event.

Table S2. **Evolution of analytic parameters after days 5, 10 or hospital discharge (whichever was first), 28 and 56 in the ITT population**

	Experimental (N=27)	Control (N=28)	p-value
**Time to normalization of analytic parameters, days**			
Lymphopenia (≤ 1.3 x10E9/L)	5 (3.75–11.2)	5 (3.75–9.75)	0.906^
CRP (≤ 5 mg/L)	7 (4.50–9.00)	4.5 (3.00–8.00)	0.345^
Ferritin (≤ 400 μg/L)	28.0 (25.5–30.5)	27.0 (26.0–47.09	0.519^
LDH (≤ 293 U/L)	9.00 (4.50–19.5)	4.00 (2.50–12.5)	0.333^
IL-6 (≤ 6.9ng/L)	37.0 (27.0–55.0)	54.0 (25.0–56.0)	0.934^
D-dimer (<250 μg/L)	0.00 (0.00–1.50)	23.0 (11.5–31.5)	0.264^
**Value of analytic parameters at day 5**			
Lymphopenia (≥ 1.3 x10E9/L)	1.52 (0.94–1.80)	1.20 (0.74–1.51)	0.088*
CRP (≤ 5 mg/L)	12.8 (6.98–22.4)	9.20 (2.30–17.80)	0.243*
Ferritin (≤ 400 μg/L)	1377 (878–1750)	1087 (754–1537)	0.351*
LDH (≤ 293 U/L)	397 (119)	387 (204)	0.829*
IL-6 (≤ 6.9ng/L)	1167 (166–1664)	366 (119–989)	0.441*
D-dimer (<250 μg/L)	695 (263–2590)	649 (392–1440)	0.982*
**Value of analytic parameters at day 10 or discharge**			
Lymphopenia (≥ 1.3 x10E9/L)	1.50 (0.72–2.19)	1.47 (0.88–1.74)	0.774*
CRP (≤ 5 mg/L)	3.70 (1.90–7.30)	3.20 (1.60–5.60)	0.620*
Ferritin (≤ 400 μg/L)	1271 (904–1744)	1112 (688–1350)	0.121*
LDH (≤ 293 U/L)	348 (154)	325 (128)	0.578*
IL-6 (≤ 6.9ng/L)	207 (44.5–648)	51.4 (48.9–118)	0.558*
D-dimer (<250 μg/L)	665 (253–852)	455 (250–907)	0.731*
**Value of analytic parameters at day 28**			
Lymphopenia (≥ 1.3 x10E9/L)	1.57 (1.06–1.88)	1.67 (1.41–2.00)	0.307*
CRP (≤ 5 mg/L)	1.60 (0.65–26.8)	3.5 (1.05–9.30)	0.820*
Ferritin (≤ 400 μg/L)	452 (267–650)	350 (279–500)	0.511*
LDH (≤ 293 U/L)	245 (65.9)	247 (57.7)	0.915*
IL-6 (≤ 6.9ng/L)	7.50 (4.80–16.2)	14.4 (7.30–28.9)	0.220*
D-dimer (<250 μg/L)	250 (250–375)	250 (250–275)	0.326*
**Value of analytic parameters at day 56**			
Lymphopenia (≥ 1.3 x10E9/L)	1.75 (1.41–2.18)	1.92 (1.62–2.22)	0.360*
CRP (≤ 5 mg/L)	2.35 (0.63–3.70)	1.45 (0.70–3.60)	0.724*
Ferritin (≤ 400 μg/L)	275 (144–426)	170 (133–290)	0.205*
LDH (≤ 293 U/L)	208 (47.9)	211 (47.5)	0.814*
IL-6 (≤ 6.9ng/L)	3.65 (2.20–4.82)	3.25 (2.50–4.60)	0.925*
D-dimer (<250 μg/L)	250 (250–376)	250 (250–260)	0.462*

* Normally distributed continuous data are reported as the mean (SD) and compared using T-test. Non-normally distributed continuous data are reported as the median (interquartile range) and compared using the Wilcoxon test. ^Asymptotic Log-Rank test. Abbreviations: CRP = C-reactive protein; IL-6 = interleukin-6; LDH = lactate dehydrogenase.

Table S3. **Changes in cytokine profile at randomization, and at days 14, 28 and 56 in the ITT population.**

	Experimental (N=27)	Control (N=28)	p-value
CXCL9 (<879 pg/ml)			
at day 0	652 (390–871)	310 (284–517)	0.067
at day 14	479 (382–632)	502 (284–839)	0.521
at day 28	283.5 (283.5–283.5)	283.5 (283.5–283.5)	0.637
at day 56	64.43 (64.43–403.33)	171.79 (64.43–283.5)	0.640
CXCL10 (<21.6 pg/ml)			
at day 0	274 (174–734)	186 (63.9–403)	0.161
at day 14	92.3 (63.9–260)	157 (54.2–236)	0.692
at day 28	71.59 (40.02–104.29)	51.09 (38.91–91.59)	0.651
at day 56	59.27 (38.64–81.02)	55.8 (37.38–81.73)	0.855
G-CSF (<54.6 pg/ml)			
at day 0	26.1 (11.9–65.2)	11.9 [11.9–34.0)	0.175
at day 14	11.9 (11.9–13.5)	11.9 (11.9–16.2)	0.950
at day 28	31.28 (18.42–39.57)	25.79 (22.81–39.57)	0.894
at day 56	25.79 (25.79–25.79)	25.79 (17.37–27.69)	0.421
IFN-alpha (<10.9 pg/ml)			
at day 0	5.45 (3.21–5.45)	5.45 (2.21–5.45)	0.583
at day 14	4.71 (4.08–5.45)	5.35 (4.71–5.45)	0.417
at day 28	5.45 (5.45–5.45)	5.45 (5.45–5.45)	0.317
at day 56	5.45 (0.82–5.45)	4.19 (0.82–5.45)	0.395
IFN-beta (<12.3 pg/ml)			
at day 0	0.31 (0.31–6.15)	3.97 (0.31–6.15)	0.882
at day 14	6.15 (3.73–6.15)	6.15 (5.00–6.15)	0.776
at day 28	5.66 (3.61–6.15)	1.56 (1.56–5.91)	0.150
at day 56	6.15 (6.15–6.15)	6.15 (6.15–6.15)	0.306
IFN-gamma (<44 pg/ml)			
at day 0	14.2 (11.7–23.8)	19.1 (9.16–22.0)	0.656
at day 14	12.2 (8.56–19.6)	20.9 (11.5–22.4)	0.030
at day 28	21.95 (21.95–21.95)	21.95 (21.95–21.95)	..
at day 56	21.95 (3.28–21.95)	21.95 (6.49–21.95)	0.925
IL-1 beta (<15.8 pg/ml)			
at day 0	8.13 (6.64–9.60)	6.64 (5.13–9.60)	0.243
at day 14	4.65 (3.11–6.64)	4.87 (3.11–6.64)	0.756
at day 28	7.9 (7.9–7.9)	7.9 (7.9–7.9)	..
at day 56	7.9 (7.9–7.9)	7·9 (7.9–7.9)	0.544
IL-1RA (<960 pg/ml)			
at day 0	2440 (1252–4785)	2305 (944–3745)	0.708
at day 14	1967 (844–3864)	2257 (1519–3192)	0.356
at day 28	1135.55 (761.72–2304.73)	1580.28 (908.7–1997.31)	0.672
at day 56	887.17 (631.3–1333.96)	991.4 (672.39–1247.06)	0.865
IL-2R alpha (<725 pg/ml)			
at day 0	1297 (977–1782)	1281 (1001–1598)	0.783
at day 14	896 (710–1366)	950 (782–1392)	0.843
at day 28	547.85 (457.49–958.5)	583.14 (483.04–759.36)	0.930
at day 56	626.88 (467.08–902)	490.96 (393.43–681.37)	0.175

IL-6 (<4.2 pg/ml)			
at day 0	40.2 (20.8–251)	23.9 (12.3–352)	0.372
at day 14	161 (17.3–430)	108 (22.9–583)	0.965
at day 28	2.43 (1.85–6.86)	4.82 (2.4–9.75)	0.313
at day 56	1.17 (0.56–2.38)	1.78 (1.02–2.1)	0.640
IL-8 (<28.3 pg/ml)			
at day 0	9.02 (6.54–17.6)	8.03 (4.56–15.1)	0.396
at day 14	1.90 (1.90–3.65)	1.90 (1.90–2.86)	0.761
at day 28	3.56 (2.03–4.5)	3.11 (2.2–4.87)	0.838
at day 56	4.29 (2.48–5.97)	2.99 (1.96–5.06)	0.251
IL-10 (<4 pg/ml)			
at day 0	11.5 (8.48–26.4)	9.09 (3.51–19.9)	0.217
at day 14	3.50 (1.97–9.08)	5.54 (2.92–9.59)	0.397
at day 28	2 (1.5–3.74)	2 (1.89–2)	0.550
at day 56	2.7 (1.91–3.47)	2.7 (1.91–4.22)	0.733
IL-18 (<788 pg/ml)			
at day 0	914 (640–1234)	893 (661–1418)	0.749
at day 14	593 (423–805)	753 (450–1037)	0.482
at day 28	529.22 (387.84–706.75)	524.82 (341.14–850.75)	0.804
at day 56	450.23 (385.79–829.66)	554.65 (395.05–667.64)	0.938
MCP-1 (<499.2 pg/ml)			
at day 0	331 (189–562)	313 (196–479)	0.683
at day 14	315 (145–564)	221 (113–467)	0.429
at day 28	207 (132–249)	166 (132–283)	0.930
at day 56	192 (162–290)	171 (141–234)	0.397
MCP-3 (<55.1 pg/ml)			
at day 0	29.1 (1.14–29.1)	29.1 (1.14–35.3)	0.486
at day 14	29.1 (29.1–29.1)	29.1 (29.1–29.1)	0.668
at day 28	29.1 (29.1–29.1)	29.1 (29.1–29.1)	..
at day 56	29.1 (29.1–29.1)	29.1 (29.1–29.1)	..
TNF-alpha (<8.1 pg/ml)			
at day 0	8.99 (7.58–11.8)	8.29 (6.87–11.1)	0.550
at day 14	7.26 (5.99–9.38)	8.89 (4.65–10.9)	0.636
at day 28	3.25 (2.97–4.95)	3.25 (1.85–4.95)	0.297
at day 56	5.67 (4.49–6.27)	5.08 (4.34–5.82)	0.304

Data are reported as the median (interquartile range) and compared using the Wilcoxon test. Results less than the lower limit of the custom bead-based Luminex multiplex immunoassay were imputed with half of the normal value. Abbreviations: CXCL = chemokine (C-X-C motif) ligand; G-CSF = granulocyte colony-stimulating factor; IFN = interferon; IL = interleukin; MCP = monocyte chemoattractant protein; R = receptor; RA = receptor antagonist; TNF = tumor necrosis factor.

Table S4. Chest X-ray at baseline and at day 56 in the ITT population.

	Experimental (N= 27)	Control (N=28)	*p*-value
**INCLUSION**			
Upper zones	2.0 (0.5–4.0)	2.0 (0.0–4.0)	0.966
Right	1 (0–2)	1 (0–2)	0.972
Left	1 (0–2)	1 (0–2)	0.915
Middle zones	5.0 (4.0–5.5)	4.0 (3.0–5.0)	0.131
Right	2 (2–3)	2 (1–2.25)	0.042
Left	2 (2–3)	2 (1–3)	0.583
Lower zones	6.0 (5.0–6.0)	6.0 (5.0–6.0)	0.611
Right	3 (2.5–3)	3 (2–3)	0.632
Left	3 (3–3)	3 (3–3)	1.000
**Total**	12.0 (10.0–15.0)	11.5 (9–14.5)	0.526
**DAY +56**			
Upper zones	0 (0–0)	0.0 (0.0–1.0)	0.322
Right	0 (0–0)	0 (0–0)	0.408
Left	0 (0–0)	0 (0–1)	0.177
Middle zones	1.0 (0.0–2.0)	1.5 (0.0–2.0)	0.466
Right	0.5 (0–1)	1 (0–1)	0.494
Left	0 (0–1)	0.5 (0–1)	0.428
Lower zones	2.0 (1.0–2.7)	2.0 (1.0–3.0)	0.361
Right	1 (1–1.75)	1 (1–2)	0.688
Left	1 (0–1)	1 (0–2)	0.278
**Total**	3 (1–4.75)	4 (2–5)	0.304

Pulmonary parenchyma involvement using chest x-ray pulmonary severity score. Data are reported as the median (interquartile range) and compared using the Wilcoxon test.

Table S5. **SARS-CoV-2 positive test and viral load by quantitative PCR in the upper respiratory tract and blood samples in the ITT population.**

	Experimental		Control	
**Upper respiratory tract samples**	**Positive, n (%)**	**Viral load, median (IQR)**	**Positive, n (%)**	**Viral load, median (IQR)**
at day 0	24/24 (100%)	244954 (8566–7458132)	20/20 (100%)	388329 (22551–980683)
at day 28	4/19 (21.05%)	0 (0–0)	2/18 (11.11%)	0 (0-0)
at day 56	1/17 (5.88%)	0 (0–0)	1/20 (5.0%)	0 (0–0)
**Blood samples**				
At day 0	2/24 (8.3%)	0 (0–0)	1/21 (4.7%)	0 (0–0)
at day 28	0/19 (0%)	0 (0–0)	1/19 (5.3%)	0 (0–0)
at day 56	1/22 (4.5%)	0 (0–0)	2/19 (10.5%)	0 (0–0)

Data are reported as number of adverse events (%) and median (interquartile range). Results of patients with viral-negative RNA are imputed with 0 $\log_{10}$ copies per mL.

Table S6. **Summary of adverse events and serious adverse events in the ITT population**

	Experimental (N=27)		Control (N=28)	
	Non-Serious	Serious	Non-Serious	Serious
Number of AE per patient	1 [1–3]	0 [0–1]	2 [1–3]	0 [0–1]
Number of AE	42	20	42	13
Number of patients reporting AE	23(85.2%)	9(33.3%)	23(82.1%)	10(35.7%)
**SOC/LLT***				
**Infections and infestations**	**16(38%)**	**7(35%)**	**10(24%)**	**3(23%)**
Bacterial prostatitis	0(0%)	0(0%)	0(0%)	1(8%)
Bacteraemia	1(2%)	0(0%)	3(7%)	0(0%)
Candida infection	0(0%)	1(5%)	0(0%)	0(0%)
Catheter-related infection	1(2%)	1(5%)	1(2%)	0(0%)
Cystitis	1(2%)	0(0%)	1(2%)	0(0%)
Cytomegalovirus infection	0(0%)	2(10%)	0(0%)	0(0%)
Lower respiratory tract infection (excluding pneumonia)	4(9%)	0(0%)	1(2%)	0(0%)
Oral candidiasis	2(5%)	0(0%)	0(0%)	0(0%)
Pneumonia, necrotizing	0(0%)	1(5%)	0(0%)	0(0%)
Pneumonia, *Pseudomonas aeruginosa*	0(0%)	0(0%)	0(0%)	1(8%)
Pseudomonal bacteraemia	0(0%)	1(5%)	0(0%)	0(0%)
Staphylococcus aureus septicaemia	0(0%)	1(5%)	0(0%)	1(8%)
Upper respiratory tract infection	7(17%)	0(0%)	3(7%)	0(0%)
Vulval infection	0(0%)	0(0%)	1(2%)	0(0%)
**Respiratory, thoracic and mediastinal disorders**	**3(7%)**	**7(35%)**	**0(0%)**	**6(46%)**
Acute respiratory distress syndrome **	0(0%)	4(20%)	0(0%)	4(31%)
Effusion, pleural	1(2%)	0(0%)	0(0%)	0(0%)
Hemoptysis	0(0%)	1(5%)	0(0%)	0(0%)
Pneumothorax	1(2%)	1(5%)	0(0%)	1(8%)
Pneumonia, interstitial	1(2%)	0(0%)	0(0%)	0(0%)
Pulmonary embolism	0(0%)	1(5%)	0(0%)	1(8%)
**Vascular disorders**	**5(12%)**	**2(10%)**	**3(7%)**	**2(15%)**
Aneurysm of abdominal aorta	0(0%)	1(5%)	0(0%)	0(0%)
Hematoma	0(0%)	0(0%)	2(5%)	0(0%)
Hypertension	4(10%)	0(0%)	1(2%)	0(0%)
Thrombosis of leg, deep venous	1(2%)	1(5%)	0(0%)	2(15%)
**Cardiac disorders**	**2(5%)**	**0(0%)**	**5(12%)**	**2(15%)**
Atrial fibrillation	1(2%)	0(0%)	4(10%)	0(0%)
Cardiac tamponade	0(0%)	0(0%)	0(0%)	1(8%)
Chest pain - cardiac	0(0%)	0(0%)	1(2%)	0(0%)
Hemopericardium	0(0%)	0(0%)	0(0%)	1(8%)
Paroxysmal tachycardia (supraventricular)	1(2%)	0(0%)	0(0%)	0(0%)
**Gastrointestinal disorders**	**3(7%)**	**0(0%)**	**1(2%)**	**0(0%)**
Diarrhea	2(5%)	0(0%)	0(0%)	0(0%)
Gastroesophageal reflux	1(2%)	0(0%)	0(0%)	0(0%)
Oral ulceration	0(0%)	0(0%)	1(2%)	0(0%)
**Metabolism and nutrition disorders**	**7(17%)**	**0(0%)**	**12(29%)**	**0(0%)**
Steroid diabetes	0(0%)	0(0%)	1(2%)	0(0%)
Hyperglycemia	6(14%)	0(0%)	10(24%)	0(0%)
Hospital acquired hyponatremia	1(2%)	0(0%)	1(2%)	0(0%)

**Blood and lymphatic system disorders**	**1(2%)**	**0(0%)**	**3(7%)**	**0(0%)**
Neutropenia	1(2%)	0(0%)	0(0%)	0(0%)
Thrombocytopenia	0(0%)	0(0%)	3(7%)	0(0%)
**Hepatobiliary disorders**	**0(0%)**	**0(0%)**	**2(5%)**	**0(0%)**
Cholestasis	0(0%)	0(0%)	1(2%)	0(0%)
Hepatitis acute	0(0%)	0(0%)	1(2%)	0(0%)
**Nervous system disorders**	**1(2%)**	**2(10%)**	**1(2%)**	**0(0%)**
Hemorrhagic stroke	0(0%)	1(5%)	0(0%)	0(0%)
Confusional state	1(2%)	1(5%)	1(2%)	0(0%)
**General disorders and administration site conditions**	**1(2%)**	**0(0%)**	**0(0%)**	**0(0%)**
Edema of lower extremities	1(2%)	0(0%)	0(0%)	0(0%)
**Injury, poisoning and procedural complications**	**0(0%)**	**0(0%)**	**1(2%)**	**0(0%)**
Post procedural hematuria	0(0%)	0(0%)	1(2%)	0(0%)
**Investigations**	**2(5%)**	**0(0%)**	**1(2%)**	**0(0%)**
Strongyloides IgG antibody positive	2(5%)	0(0%)	1(2%)	0(0%)
**Musculoskeletal and connective tissue disorders**	**1(2%)**	**0(0%)**	**0(0%)**	**0(0%)**
Pain in lumbar spine	1(2%)	0(0%)	0(0%)	0(0%)
**Renal and urinary disorders**	**0(0%)**	**2(10%)**	**2(5%)**	**0(0%)**
Failure kidney acute	0(0%)	2(10%)	2(5%)	0(0%)
**Skin and subcutaneous tissue disorders**	**0(0%)**	**0(0%)**	**1(2%)**	**0(0%)**
Skin rash	0(0%)	0(0%)	1(2%)	0(0%)

Adverse events were coded with the Medical Dictionary for Regulatory Activities (MedDRA)© version-23.0. Abbreviations: SOC: *System Organ Classes – LLT: Lowest Level Term. Data are shown as number of adverse events (%) and include all events reported after randomization. Some patients had more than one adverse event; **COVID-19 acute respiratory distress syndrome.

Table S7. **8-point ordinal scale at 10, 28 and 58 days in the ITT population.**

	Experimental (N=27)	Control (N=28)
**8-point ordinal scale at day 5**		
1–3. Discharge or hospital admission not requiring supplemental oxygen and no longer requiring ongoing medical care	0(0%)	0(0%)
4. Hospital admission, not requiring supplemental oxygen but requiring ongoing medical care	2(7.41%)	1(3.57%)
5. Hospital admission, requiring any supplemental oxygen	16(59.3%)	14(50.0%)
6. Hospital admission, requiring non-invasive ventilation or use of high-flow oxygen devices	5(18.5%)	7(25.0%)
7. Hospital admission, requiring invasive mechanical ventilation or ECMO	4(14.8%)	3(10.7%)
8. Death	0(0%)	3(10.7%)
**8-point ordinal scale at day 10**		
1–3. Discharge or hospital admission not requiring supplemental oxygen and no longer requiring ongoing medical care	9(33.0%)	7(25.0%)
4. Hospital admission, not requiring supplemental oxygen but requiring ongoing medical care	1(3.70%)	1(3.57%)
5. Hospital admission, requiring any supplemental oxygen	8(29.6%)	7(25.0%)
6. Hospital admission, requiring non-invasive ventilation or use of high-flow oxygen devices	3(11.1%)	6(21.4%)
7. Hospital admission, requiring invasive mechanical ventilation or ECMO	4(14.8%)	2(7.14%)
8. Death	2(7.41%)	5(17.9%)
**8-point ordinal scale at day 28**		
1–3. Discharge or hospital admission not requiring supplemental oxygen and no longer requiring ongoing medical care	18(66.7%)	18(64.3%)
4. Hospital admission, not requiring supplemental oxygen but requiring ongoing medical care	0(0%)	1(3.57%)
5. Hospital admission, requiring any supplemental oxygen	1(3.70%)	2(7.14%)
6. Hospital admission, requiring non-invasive ventilation or use of high-flow oxygen devices	2(7.41%)	0(0%)
7. Hospital admission, requiring invasive mechanical ventilation or ECMO	2(7.41%)	1(3.57%)
8. Death	4(14.8%)	6(21.4%)
**8-point ordinal scale at day 56**		
1–3. Discharge or hospital admission not requiring supplemental oxygen and no longer requiring ongoing medical care	18(66.7%)	21(75.0%)
4. Hospital admission, not requiring supplemental oxygen but requiring ongoing medical care	1(3.7%)	0(0%)
5. Hospital admission, requiring any supplemental oxygen	2(7.41%)	1(3.6%)
6. Hospital admission, requiring non-invasive ventilation or use of high-flow oxygen devices	0(0%)	0(0%)
7. Hospital admission, requiring invasive mechanical ventilation or ECMO	1(3.7%)	0(0%)
8. Death	5(18.5%)	6(21.4%)

Data are reported as number of adverse events (%). 8-point ordinal scale: 1 = Not hospitalized, no limitations on activities; 2 = Not hospitalized, limitation on activities and/or requiring home oxygen; 3 = Hospitalized, not requiring supplemental oxygen – no longer requires ongoing medical care; 4 = Hospitalized, not requiring supplemental oxygen – requiring ongoing medical care; 5 = Hospitalized, requiring supplemental oxygen; 6 = Hospitalized, on non-invasive ventilation or high-flow oxygen devices; 7 = Hospitalized, on mechanical ventilation or extracorporeal membrane oxygenation (ECMO); 8 = Death.
